# Site-Selective Protein Modification via Peptide-Directed Proximity Catalysis

**DOI:** 10.1021/acsomega.5c07883

**Published:** 2025-12-18

**Authors:** Laetitia Raynal, Joe Nabarro, Lisa M. Miller, Adam A. Dowle, Sophie L. Moul, Phathutshedzo Masithi, Steven D. Johnson, Martin A. Fascione, Christopher D. Spicer

**Affiliations:** † Department of Chemistry, 8748 University of York, Heslington YO10 5DD, U.K.; ‡ York Biomedical Research Institute, University of York, Heslington YO10 5DD, U.K.; § School of Physics, Engineering, and Technology, University of York, Heslington YO10 5DD, U.K.; ∥ Bioscience Technology Facility, Department of Biology, University of York, Heslington YO10 5DD, U.K.

## Abstract

Proximity catalysis exploits ligand binding for localized, catalytic protein modification. In this work, we introduce catalyst-functionalized peptides as versatile ligands for this approach. Through the functionalization of target-binding peptides with pyridinium oxime catalysts, we show that model proteins can be site-selectively modified with a variety of *N*-acyl-*N*-alkylsulfonamide reagents to introduce common functionalities, including fluorophores and affinity handles to the protein surface. Critically, we show that simple changes to the peptide-catalyst structure, moving the pyridinium oxime from the N- to C-terminus, alter the site of modification. This opens up possibilities to develop peptide libraries for a particular target protein and subsequently tuning the modification site for a given application.

## Graphical abstract

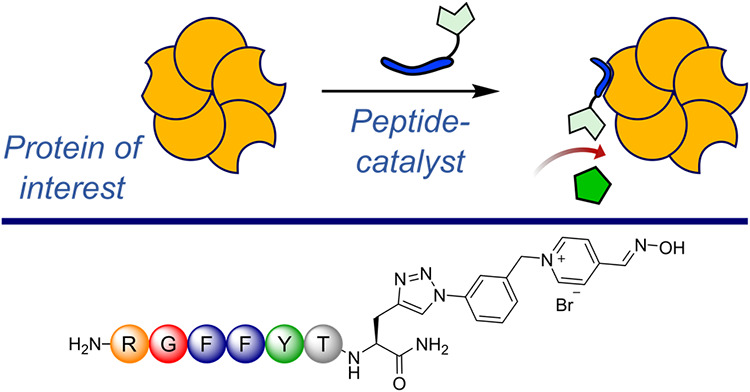

## Introduction

Site-selective chemical modification remains a crucial challenge in the development of protein-based technologies and tools. Chemo-selective approaches to achieve single-site labeling typically rely on the introduction of a uniquely reactive amino acid, most commonly a solvent-exposed cysteine or an unnatural reactive handle via codon reassignment.^1,2^ Though these methods are powerful, they are limited by the requirement for prior genetic engineering of the target protein, and are therefore poorly suited to proteins isolated from natural sources, or of eukaryotic origin where recombinant expression may be challenging.^3^ As a result, there has been growing interest in the development of regio-selective approaches that can specifically target an amino acid on a protein surface, even in the presence of other residues bearing the same functional groups.^4^

Of these regio-selective approaches, proximity-mediated strategies have proved most powerful to date. In these strategies, a moderately reactive species is brought into close proximity with a protein surface via some form of ligand binding, creating a pseudointramolecular environment that enables labeling reactions that would not otherwise take place without this effective concentration.^5^

In most cases, small-molecule ligands are used to mediate protein binding, and as a result, modification is most commonly (though not exclusively) in proximity to the protein active site.^6^ More recently, peptide-based ligands have found utility in proximity labeling^7–10^ providing three potential advantages: (i) with ongoing development in screening technologies such as phage and mRNA display, peptides can be “evolved” to bind to most target proteins;^11^ (ii) the lack of bias during this screening enables the identification of peptide ligands that bind away from the protein active site. Subsequent labeling at these sites is less likely to adversely affect protein activity;^12^ and (iii) solid-phase synthesis streamlines the synthesis and functionalization of peptides, reducing the challenges of ligand development. Peptides therefore provide a potential route to highly generalizable and translatable protein modification strategies, which can be widely applied to proteins of diverse origin and function.

In this paper, we report an example of peptide-based proximity catalysis, a powerful extension of traditional proximity modifications that decouples the processes of ligand binding and subsequent protein labeling (Figure 1).^7,13,14^ In doing so, it enables the introduction of a diverse range of labels using a single ligand-catalyst species. We demonstrate that different sites on the protein surface can be targeted by tuning the structure of the peptide-catalyst and that this versatile technology can be applied to a number of different protein targets. We anticipate that our peptide-directed proximity catalysis approach will provide a valuable addition to the toolbox of reactions for site-specific protein modification, particularly for targets that cannot be genetically engineered.

**Figure 1:**
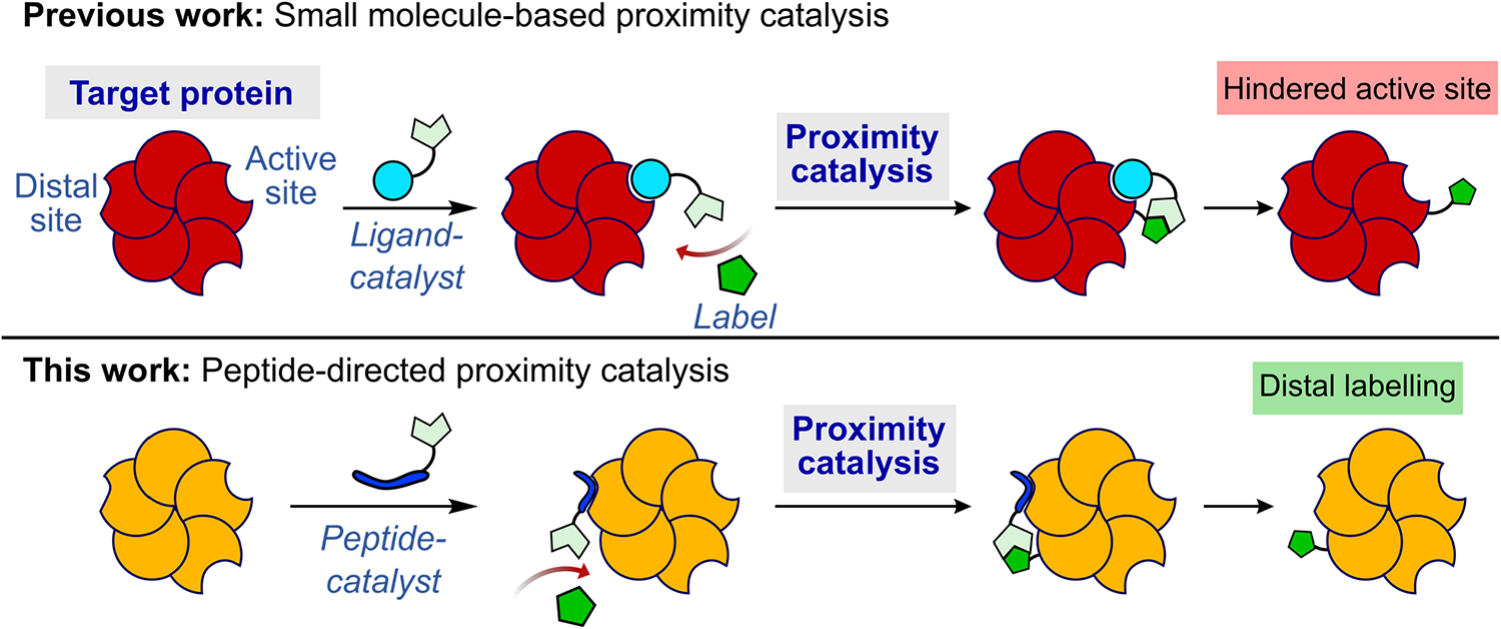
Overview of the use of peptide-directed proximity catalysis to mediate site-selective protein labeling distal to the active site. By comparison, traditional small-molecule ligands most commonly bind and induce labeling of the active site, potentially interfering with protein activity.

## Results and Discussion

Our initial project design was inspired by the work of the Hamachi group, who over the past 15 years have pioneered the development of both ligand-directed chemistries and proximity catalysis.^5,15,16^ Of the catalyst systems reported, we were particularly attracted to the use of pyridinium oximes, which can catalyze protein acylation with *N*-acyl-*N*-alkylsulfonamide (NASA) reagents in a proximity-dependent manner.^17^ NASAs have been shown to possess improved stability relative to alternative reagents for protein acylation, such as thioesters, while pyridinium oxime catalysts provide faster protein labeling than alternatives such as dimethylaminopyridine- or rhodium-catalysts.^5^

We envisaged introducing pyridinium oximes at strategic positions within our target-binding peptides during their synthesis, which could then be used to modify the target protein with a diverse set of NASA-functionalized labels. As an initial target on which to develop our approach, we chose human insulin. Although insulin can be expressed recombinantly, and genetic engineering is therefore plausible, its small size made it an ideal model. A number of peptide sequences have previously been reported to bind insulin.^18–20^ Of these, we chose to focus on the peptide RGFFYT (**P1**). This sequence is actually derived from insulin itself, playing a role in insulin self-assembly and oligomerization, and has been computationally predicted to bind parallel to the insulin B-chain with nM affinity.^20^ We hypothesized that the introduction of a catalyst at either end of the peptide would lead to significant differences in the preferred site of insulin labeling. A similar strategy has recently been reported by Kim et al., who introduced PyOx catalysts at different positions within a Z-domain specific affibody, leading to differences in both labeling site and efficiency on its Z-domain binding partner.^21^ In this case, genetic code expansion was required to install the PyOx catalyst, whereas the benefits of solid-phase synthesis greatly facilitate this installation within the peptides reported here. However, this work also highlights the need to consider both the location and local environment of a residue within the target protein, and the ability to generate multiple peptide catalysts from a single sequence is therefore advantageous.

To predict whether the insulin-binding capability of **P1** would be maintained after the introduction of a pyridinium oxime catalyst, peptides bearing either an N- or C-terminal cysteine (**C-P1** and **P1**-**C**, respectively) were first synthesized and tethered to a gold sensor for quartz crystal microbalance with dissipation monitoring (QCM-D) analysis. It is important to note that these model peptides lacked the positive charge that would be introduced upon installation of a pyridinium oxime, and so evidence of binding was expected to be indicative of binding potential, rather than providing an exact measure of binding affinity. Upon addition of insulin, a characteristic binding response was observed for both **C-P1** and **P1-C** with *K*_d_s in the low μM range, suggesting the introduction of the pyridinium oxime catalyst at either terminus had the potential to be tolerated (Supporting Information, Figures S1 and S2).

Peptides were subsequently synthesized bearing either a C-terminal propargylglycine or an N-terminal 5-pentynoic acid (**P1-alkyne** and **alkyne-P1**, respectively, Figure 2a). *p*-Nitrophenyl NASA-reagents were also synthesized bearing hexanoic acid (**3a**), biotin (**3b**), nitrobenzoxadiazole (NBD, **3c**), and Cy5 (**3d**) acyl groups, as model labels for subsequent protein modification (Figure 2b).^17^

**Figure 2:**
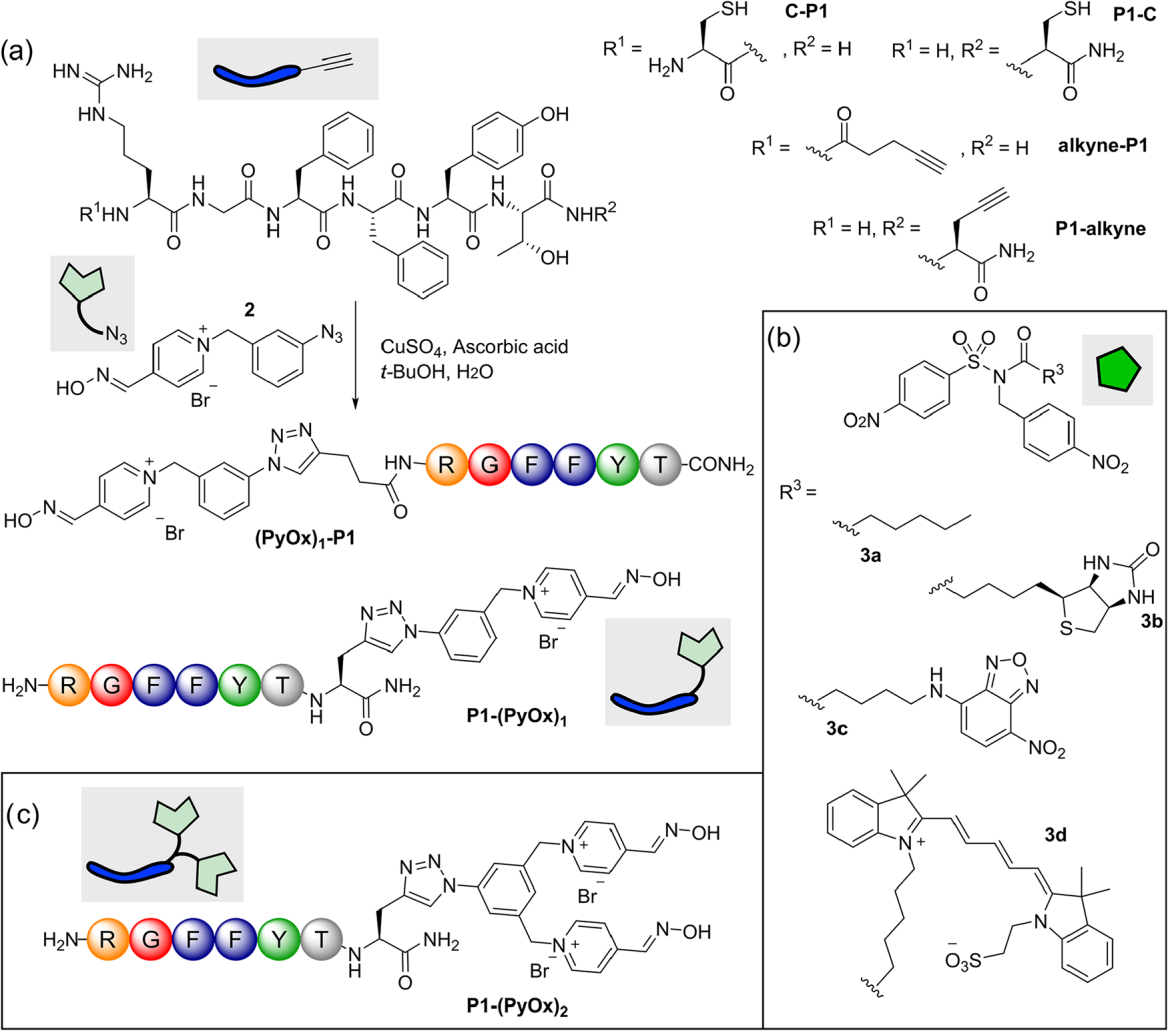
(a) Synthesis of pyridinium oxime-functionalized analogues of peptide **P1** via copper-catalyzed azide–alkyne cycloaddition; (b) *N*-acyl-*N*-alkylsulfonamide (NASA) reagents synthesized and used in this work; (c) bis-pyridinium oxime peptide **P1-(PyOx)**_**2**_.

With these reagents in hand, we attempted peptide-directed proximity catalysis on insulin. Initial experiments were performed in pH 7.4 phosphate buffer at a concentration of 50 μM, which was above the *K*_d_ calculated for peptide binding (Figure 3). Varying equivalents of pyridinium oxime peptide **P1-(PyOx)**_**1**_ (0, 1.25, 2.5, 5, or 10 equiv wrt protein) were then added, followed by the addition of biotin–NASA **3b** (20 equiv wrt protein). Loadings of the NASA were limited by the poor solubility of **3b** in aqueous media, requiring presolubilization in dimethyl sulfoxide (DMSO), which was subsequently diluted in the reaction mixture to a final concentration of ∼1% v/v. After 2 h at room temperature (22 °C), reactions were analyzed by intact protein liquid chromatography mass spectrometry (LC-MS). Pleasingly, in the presence of both **P1-(PyOx)**_**1**_ and **3b**, a mass corresponding to acylation of insulin with biotin was observed (entries 2–5; 14–36%). Five equiv of peptide was found to be optimal, potentially due to the need to balance protein-bound vs solution-phase activation of the NASA species, which would in turn lead to hydrolysis of the acyl-oxime intermediate (entry 4). Notably, even at high peptide loadings, no evidence of dual modification was observed by LC-MS. In the absence of **P1-(PyOx)**_**1**_, there was no labeling (entry 1), demonstrating that acylation was peptide-mediated and that background reactivity of the NASA was minimal under these conditions. Further controls using either a scrambled peptide (FYRTGF-(PyOx)_1_), peptide lacking the pyridinium oxime **P1**, or pyridinium oxime-azide **2** also gave no labeling, providing further evidence that labeling was mediated by a specific protein–peptide binding interaction, and catalyzed by the pyridinium oxime motif.

**Figure 3:**
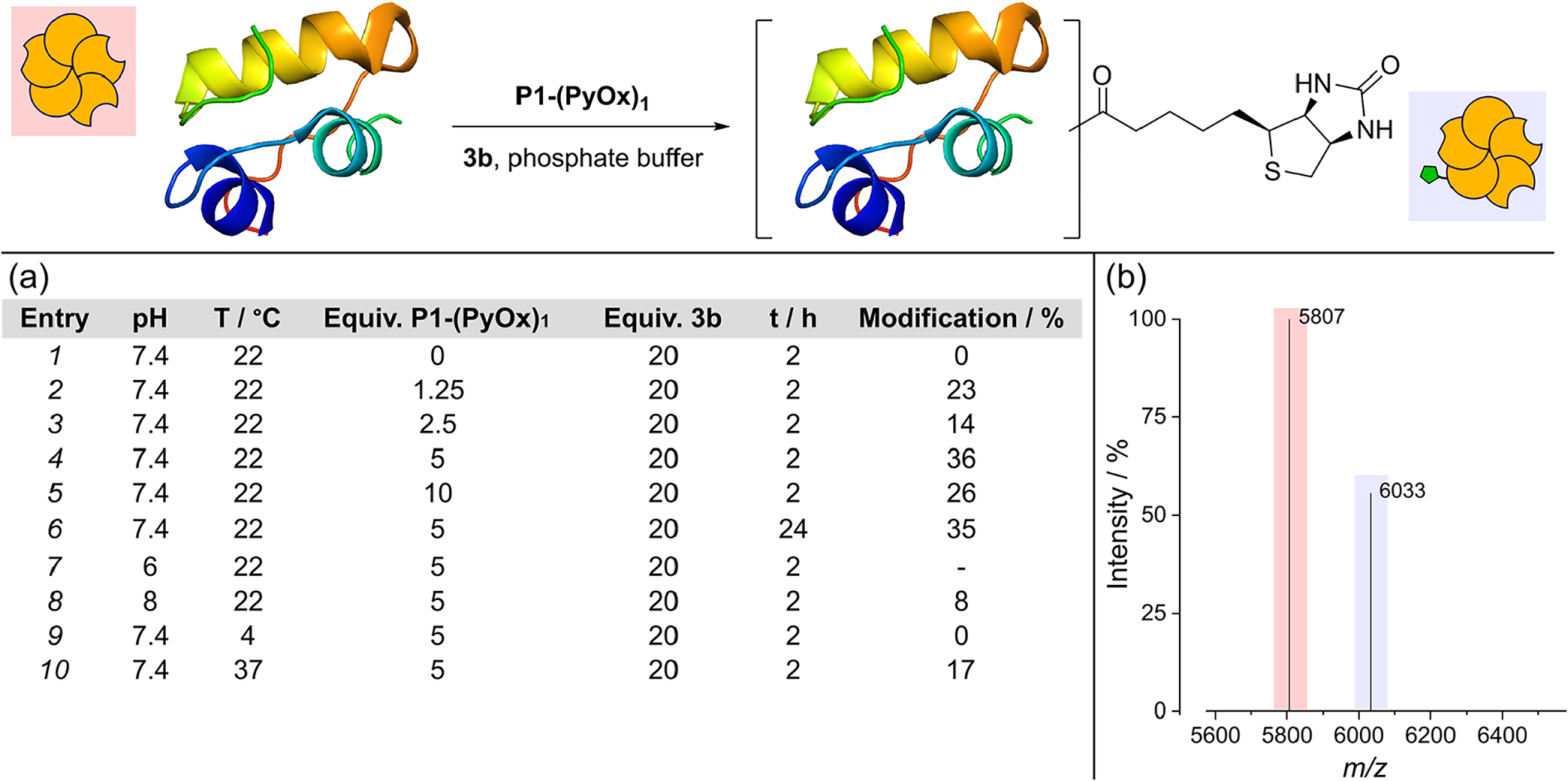
Optimisation of biotin labeling of insulin via peptide-directed catalysis with peptide **(PyOx)**_**1**_ **-P1** and NASA reagent **3b**; (a) table of selected conditions and percentage modifications determined by LC-MS analysis of crude reaction mixtures; (b) representative deconvoluted mass spectra for entry 4. Insulin Calcd: 5807, Observ.: 5807 Da; insulin–biotin Calcd: 6034, Observ.: 6033.

No difference in conversion was observed if reactions were extended to 24 h (entry 6), consistent with the limited hydrolytic stability of the intermediate oxime-acyl complex formed during catalysis and the recent report of Kim et al.^21^ However, following these extended periods very low levels of background labeling were observed in the absence of peptide (<2%), and so 2 h was chosen as a reaction length for all future experiments.

We next looked to optimize the reaction conditions to maximize conversion, while being cognizant of the risks of background modification resulting from nonspecific labeling, rather than mediated by a protein-bound peptide catalyst. Labeling at pH 6 was not possible due to the decreased solubility of insulin at this pH, leading to a loss of protein MS signals even in the absence of peptide or NASA (entry 7).^22^ At pH 8, labeling efficiency decreased relative to pH 7.4. *p*-Nitrophenyl NASA-reagents have been reported to have high stability at neutral pH (half-lifes of >3 days)^17^ but would be expected to be more susceptible to hydrolysis at pH 8, which may explain this difference, since the nucleophilicity of the target amino acid residues and PyOx catalyst would also be expected to increase at higher pH (entry 8).

With **P1-(PyOx)**_**1**_, no labeling was observed when lowering the reaction temperature to 4 °C. At 37 °C, modification was successful but less efficient, potentially due to an increased rate of NASA hydrolysis, and so room temperature (∼22 °C) was used in subsequent experiments (entries 9 and 10). Background labeling was also observed at 37 °C in the absence of peptide catalyst (∼7%), and so a temperature of 22 °C was used for all future studies. However, as discussed below, we expect the optimal conditions to be peptide- and protein-dependent, due to the complex interplay between NASA activation or hydrolysis, and the subsequent labeling profiles of different amino acid residues.

The conversions obtained highlight a potential drawback to the use of peptide ligands versus previously reported small-molecule-guided catalyst systems. Typically, the small-molecule ligands used possess an nM affinity for their target protein, in contrast to the predicted μM affinity of the **P1** peptide (noting the assumptions detailed above). Labeling efficiency has been previously demonstrated to correlate with *K*_d_,^16^ consistent with the moderate conversions obtained within this work. While the generation of peptides with modest affinity is achievable via standard library screening techniques, such as phage- or mRNA-display, obtaining high-affinity binders is more challenging and may require multiple rounds of optimization and/or chemical derivatization to achieve suitable ligands.^23^ In the absence of direct comparative data between the conversions obtained in this work and an analogous small-molecule-guided labeling of insulin, it is difficult to draw comparisons. However, it should be noted that the absence of well-defined and readily available small-molecule binders of insulin supports the use of our peptide-based strategy as a complementary ligand class for proximity labeling.

Using the optimized conditions for **P1-(PyOx)**_**1**_, we were also able to demonstrate labeling with NBD-NASA (**3c**). Strong fluorescent labeling of insulin was observed via in-gel fluorescence, while successful biotinylation with **3b** was also verified via antibiotin Western blotting (Supporting Information, Figures S13 and S15). Notably, the sensitivity of both fluorescent imaging and Western blotting allowed us to detect very low levels of background labeling at higher NASA loadings in the absence of **P1-(PyOx)**_**1**_ even after 2 h labeling, though this was found to be taking place at levels that were undetectable by LC-MS.

The use of catalyst **(PyOx)**_**1**_ **-P1**, sharing the same RGFFYT insulin-binding sequence but bearing the pyridinium oxime at the N-terminus, was then investigated. This peptide was also found to mediate successful insulin labeling, though with reduced conversion (25% conversion, see SI Section 4). Given the similarity in *K*_d_s between the two peptides, we attribute this difference in labeling efficiency to the availability or reactivity of suitable amino acids in proximity to the C-terminal vs N-terminal binding site. In an attempt to improve labeling efficiency, we also synthesized a peptide bearing two pyridinium oxime catalysts at the C-terminus via a bis-functional azide, to generate **P1-(PyOx)**_**2**_ (Figure 2c). Keijzer et al. have previously reported that aptamers bearing two pyridinium oximes were able to enhance protein labeling relative to the monovalent analogue.^24^ However, in contrast, we saw a reduction in labeling efficiency (19%, see SI Section 4), which may be attributed to a change in the orientation or positioning of the PyOx catalyst within the bis-functional peptide, or alternatively in the binding efficiency of the peptide to insulin. Importantly, it has been previously shown that variation in the length and rigidity of the spacer between the protein-binding ligand and the reactive group or catalyst can strongly influence the site and efficiency of protein labeling.^17,21,25^ For a particular protein target of interest, there is therefore scope to further improve or diversify labeling through variations to the PyOx catalyst structure.

Matrix-assisted laser desorption/ionization MS/MS (MALDI-MS/MS) analysis was subsequently performed to identify the sites of insulin labeling for each peptide catalyst. For N-terminal catalyst peptide **(PyOx)**_**1**_ **-P1**, labeling was found to be taking place within the first seven residues of the N-terminal region of insulin chain B (FVNQHLC, see SI Section 7, Figure 4). Within this region, the N-terminal α-amine is the most likely site for labeling, though we were not able to demonstrate this unambiguously. In contrast, using **P1-(PyOx)**_**1**_ with the catalyst at the C-terminus of the peptide, labeling was found to take place specifically at the N-terminus of insulin chain A (see SI Section 7, Figure 4). Though alterations to linker length and structure can change the site of labeling using small-molecule ligands, this labeling is still relatively confined to be within proximity to the ligand-binding site. In contrast, these results validate our hypothesis that different sites on a protein, within spatially distinct regions, can be targeted via a peptide-directed catalysis approach by tuning the peptide structure. The ease with which these peptides can be generated provides an advantage over an analogous small-molecule system.

**Figure 4:**
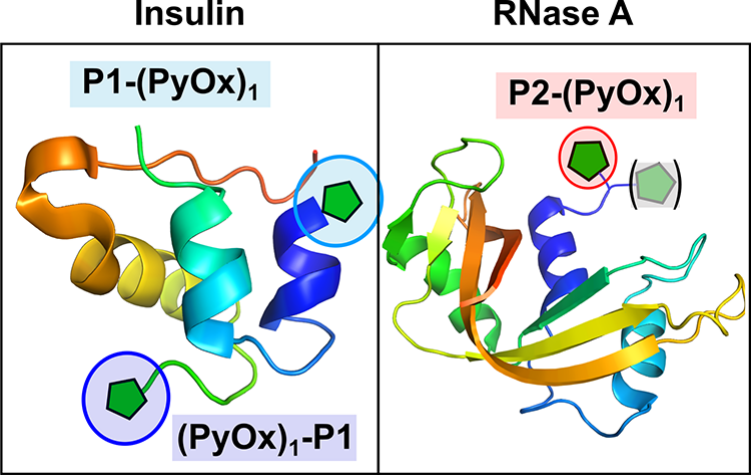
Sites of insulin and RNase labeling using the peptide catalysts developed in this study. For RNase A, a mix of mono- and dilabeling was observed due to modification of both the α- and ε-amines of the N-terminal lysine residue.

A potential limitation of using peptide-based ligands for proximity catalysis is the risk of preferential self-labeling of the peptide itself, rather than the protein, if it contains nucleophilic residues.^21^ The effects of self-labeling will differ depending on the peptide, with a decrease in protein binding affinity one possibility. Alternatively, self-labeling may have a neutral effect on *K*_d_. In this scenario, protein labeling would still be possible, albeit with a potentially reduced stoichiometry of the NASA reagent. These effects are likely to be complex, with the rates of peptide–protein binding and debinding, NASA activation, and reactivity with different amino acids on both peptide and protein contributing to the final outcome. Evidence of this was seen in control reactions in which insulin was omitted, with peptides **P1-(PyOx)**_**2**_ and **(PyOx)**_**1**_ **-P1**. In these controls, the peptides were incubated with hexanoic- (**3a**) or biotin-NASAs (**3b**) and analyzed by LC-MS at varying time points. For **P1-(PyOx)**_**2**_, a new species with mass matching the acylated-peptide was observed to increase over time (see SI Section 8). In contrast, for **(PyOx)**_**1**_ **-P1**, no modification was detected. MS/MS analysis of a labeled **P1-(PyOx)**_**1**_ complex allowed us to identify that the peptide was being modified within the first three residues of the peptide, RGF. Analogous results were obtained for experiments using hexanoic-NASA **3a**. The modification of arginine side-chains with NASA reagents has not previously been reported, and so it is likely that this labeling was taking place at the free α-amine of the N-terminus. Notably, in the case of **(PyOx)**_**1**_ **-P1**, the pyridinium oxime was introduced at the N-terminus via a pentynoic acid linker and the peptide did not possess an α-amine. This observation may therefore provide useful design criteria for peptide-binding catalysts in the future, particularly where a free N-terminus is not essential for protein-binding and can be, e.g., acetylated.

Having validated our labeling strategy on insulin, we aimed to demonstrate translatability on an alternative protein target. The S-peptide KETAAAKFERQHMDSSTSA, **P2**, can be proteolytically cleaved from the N-terminal domain of RNase A, wherein it retains nM binding affinty to the remaining parent protein, commonly referred to as RNase S.^26^ We reasoned that a low *K*_d_ would potentially make RNase S a challenging target to work with due to challenges removing the peptide postfunctionalization, but hypothesized that **P2** might retain some binding affinity for RNase A, even before proteolysis. QCM-D was again used to investigate the binding of peptides tethered to the sensor via their N- or C-termini (using the thiol-labeled peptides **HS-P2** and **P2-SH**, respectively). **P2-SH**, tethered via its C-terminus, was found to bind RNase A with low μM *K*_d_, and was therefore seen as a suitable sequence to carry forward into protein labeling experiments (Supporting Information, Figures S3 and S4). In contrast, **HS-P2**, tethered via its N-terminus, showed no RNase A binding, indicating that functionalization with a pyridinium oxime catalyst was unlikely to be tolerated. We therefore synthesized peptide **P2-(PyOx)**_**1**_ and tested its ability to modify RNase A with Cy5-functionalized NASA **3d**. Pleasingly, this occurred in the presence of 5 equiv **P2-(PyOx)**_**1**_ and 20 equiv **3d** clear fluorescent labeling of RNase A was observed via sodium dodecyl sulfate-polyacrylamide gel electrophoresis (TSDS-PAGE) (Supporting Information Figure S14). NanoLC-MS/MS analysis demonstrated that modification was happening specifically at the N-terminal lysine residue, with mono- and dimodification of this amino acid observed, due to labeling at both the free α- and ε-amines (see SI Section 7, Figure 4). This demonstrates that our peptide-directed catalysis approach can be applied to alternative protein targets, and opens up the possibility of developing a generalizable strategy for proximity-mediated protein labeling.

## Conclusions

In this work, we have demonstrated the use of functionalized peptide ligands as catalysts for proximity-dependent protein labeling. The use of peptide ligands provides a number of potential advantages: (i) labeling can be directed away from the active site of the enzyme, as epitomized by our observed N-terminal labeling of RNase A, far away from the catalytic RNA binding pocket; and (ii) simple changes to the peptide-catalyst structure, enabled by the high modularity of solid-phase peptide synthesis, can alter labeling site as demonstrated in our experiments through either N- or C-terminal functionalization of insulin-binding peptides. When coupled with the structural diversity of potential peptide ligands and the amenability of peptides to library screening against a particular protein target, this opens opportunities to tune the labeling site for a given downstream application. We envisage that our approach may therefore enable controlled, site-selective modification of otherwise challenging to label proteins, such as those of native origin that are not amenable to genetic engineering. With increasing interest in proximity-mediated catalysis in the bioconjugation community, the general approach detailed in this work can be expanded, for example, to alter the amino acid labeling preference in cases where suitable nucleophilic residues are not present in proximity to the peptide binding site, enable multisite labeling of proteins with peptides bearing two catalysts at rationally selected positions, or to promote selective modification of specific proteins in complex mixtures. We therefore believe that the technology outlined in this work represents an important new addition to the bioconjugation toolbox.

## Supplementary Material



## Notes

Experimental details, including peptide and small molecule synthesis, protein binding and labeling, and analysis of labeled constructs (PDF)
