# Supplementary material for: Site-Selective Protein Modification via Peptide-Directed Proximity Catalysis
Source: ACS Omega. 2025 Dec 18;10(51):62787–93. doi: 10.1021/acsomega.5c07883 (PMC12756790; doi:10.1021/acsomega.5c07883)

Site-selective protein modification via peptide-directed proximity catalysis

Laetitia Raynal,[a,b]* Joe Nabarro,[a,b]* Lisa M. Miller,[b,c] Adam A. Dowle,[d] Sophie L. Moul,[a,b] Phathutshedzo Masithi,[a,b] Steven D. Johnson,[b,c] Martin A. Fascione,[a,b] and Christopher D. Spicer*[a,b]

[a]Department of Chemistry, University of York, Heslington, YO10 5DD, UK.

[b]York Biomedical Research Institute, University of York, Heslington, YO10 5DD, UK.

[c]School of Physics, Engineering, and Technology, University of York, Heslington, YO10 5DD, UK.

[d]Bioscience Technology Facility, Department of Biology, University of York, Heslington, YO10 5DD, UK.

Table of contents

S3 General considerations

S4 PyOx azide synthesis

S6 NASA synthesis

S15 Peptide synthesis

S26 QCM-D

S30 Protein labelling

S34 (T)SDS-PAGE analysis

S35 Western blot analysis

S36 MALDI protein fragmentation analysis

S39 Peptide self-labelling

S44 References

S46 NMR spectra

General considerations

Proton and carbon nuclear magnetic resonance (^{1}H and ^{13}C NMR respectively) spectra were recorded on a Jeol ECX-400 (400 MHz) or Bruker AVIIIHD (500 MHz) spectrometer. NMR shifts were assigned using COSY, HSQC and HMBC spectra. All chemical shifts are quoted on the δ scale in ppm using residual solvent as the internal standard (^{1}H NMR: $CDCl_3$ = 7.26; D_2O = 4.69; DMSO-d_6 = 2.50 and ^{13}C NMR: $CDCl_3$ = 77.16, DMSO-d_6 = 39.52). Coupling constants (*J*) are reported in Hz with the following splitting abbreviations: s = singlet, d = doublet, t = triplet, q = quartet, m = multiplet, app = apparent, br = broad. Melting points (m.p.) were recorded on a Gallenkamp melting point apparatus. Infrared (IR) spectra were recorded on a Perkin Elmer UATR Two FT-IR spectrometer or a Bruker Alpha II ATR spectrometer with Opus build 8.5.29. High resolution electrospray ionisation (ESI) mass spectra (HRMS) were recorded on a Bruker Compact TOF-MS or a Jeol AccuTOF GCx-plus spectrometer. Nominal and exact *m/z* values are reported in Daltons (Da).

Thin layer chromatography (TLC) was carried out using aluminium backed sheets coated with 60 F_{254} silica gel (Merck). Visualization of the silica plates was achieved using a UV lamp (λ_{max} = 254 nm), potassium permanganate (5% $KMnO_4$ in 1M NaOH with 5% potassium carbonate), or ninhydrin (1.5% ninhydrin, 3% AcOH in *n*-butanol). Flash column chromatography was carried out using Geduran Si 60 (40-63 μm) (Merck). Mobile phases are reported as % volume of more polar solvent in less polar solvent. Reverse-phase flash column chromatography was performed on a Teledyne CombiFlash NEXTGEN 300+ system, using a Redi*Sep* Rf Gold C18Aq column. The column was pre-equilibrated with water, and the peptide purified using a gradient from 0-100% MeCN:H_2O at a flow rate of 13 mL^{-1} min. The gradient programme was dependent on the substrate purified.

Anhydrous solvents were dried using a PureSolv MD 7 Solvent Purification System. Deionized water was used for chemical reactions and for protein manipulations. All other solvents were used as supplied (Analytical or HPLC grade), without prior purification. Reagents were purchased from Sigma-Aldrich, VWR, or Fluorochem and used as supplied, unless otherwise indicated. Brine refers to a saturated solution of sodium chloride. Petrol refers to the fraction of petroleum ether boiling in the range 40-60 °C. Anhydrous magnesium sulfate ($MgSO_4$) was used as the drying agent after reaction workup unless otherwise stated.

Liquid chromatography-mass spectrometry (LC-MS) was performed on a HCTultra ETD II ion trap spectrometer, coupled to an Ultimate300 HPLC using an Accucore C18 column (150 × 2.1 mm, 2.6 µm particle size). Water (solvent A) and acetonitrile (solvent B), both containing 0.1% formic acid, were used as the mobile phase at a flow rate of 0.3 mL min^{-1}. LC traces were measured via UV absorption at 220, 270, and 280. The gradient was programmed as shown below:

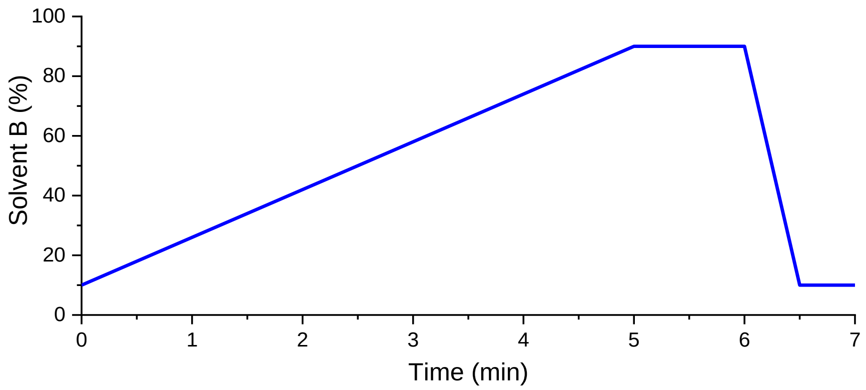

Spectra were analysed using the Bruker Data Analysis 4.4 software. Reaction conversions were determined from the integration of the UV absorption at 280 nm and the relative absorption coefficients of the products at this wavelength.

1. PyOx azide synthesis

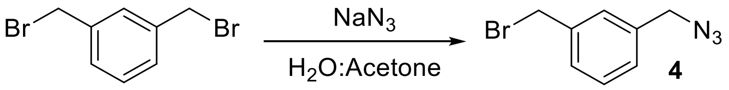

To a stirred solution of α,α'-dibromo-*m*-xylene (5.0 g, 18.9 mmol, 1 equiv.) in a mixture of acetone:water (6:1, 35 mL) was added sodium azide (1.8 g, 28.4 mmol, 1.5 equiv.). The reaction mixture was stirred for 7.5 h at room temperature, then diluted with DCM (30 mL). The organics were washed with H_2O (20 mL) and brine (20 mL), dried over Na_2SO_4, filtered, and concentrated *in vacuo*. The residue was purified by flash chromatography using 10% EtOAc:Petrol. Pure fractions were concentrated *in vacuo* to afford the product as a clear liquid (1.9 g, 8.2 mmol, 43%). Data were consistent with those previously reported.[1]

^{1}H NMR (400 MHz, $CDCl_3$): δ 7.42-7.25 (4H, m, -C<u>H</u>$_{arom}$), 4.49 (2H, s, -C<u>H$_2$</u>Br), 4.36 (2H, s, -C<u>H$_2$</u>N_3); **HRMS** (APCI): *m/z* calcd for C_8H_9BrN: 197.9913 [M+H]$^+$; found: 197.9925

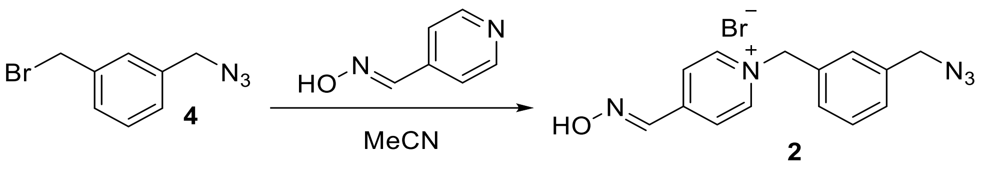

Azide **4** (750 mg, 3.33 mmol) and pyridine-4-aldoxime (800 mg, 6.56 mmol) were dissolved in MeCN (5 mL) and the mixture was stirred at 65 °C for 32 h. The mixture was then cooled to room temperature and the precipitate recovered by filtration. The precipitate was washed with MeCN (5 mL) and dried under vacuum. The residue was then purified by reverse-phase column chromatography using a gradient of 0-100% MeCN:Water. Pure fractions were concentrated *in vacuo* to afford the product **2** as a white solid (450 mg, 1.30 mmol, 39%).

^{1}H NMR (400 MHz, D_2O): δ 8.83 (2H, dd, *J* = 6.5, 1.5 Hz, Pyr H2), 8.33 (1H, d, *J* = 1.5 Hz, HC=NOH), 8.18-8.11 (2H, m, Pyr H4), 7.55-7.41 (4H, m, 4H, Phe), 5.79 (2H, s, -CH_2N^+), 4.41 (2H, d, *J* = 1.5 Hz, -CH_2N_3); **^{13}C NMR** (101 MHz, D_2O): δ 146.25 (C=NOH), 144.67 (Pyr C3), 144.56 (Pyr C3), 130.13 (Phe), 129.90 (Phe), 129.28 (Phe), 128.97 (Phe), 128.81 (Phe), 125.00 (Pyr C2), 124.98 (Pyr C2), 63.62 (-CH_2N^+), 53.75 (-CH_2N_3); **HRMS** (APCI): *m/z* calcd for $C_{14}H_{14}N_3O$: 240.1131 $[M+H]^+$; found: 240.1137.

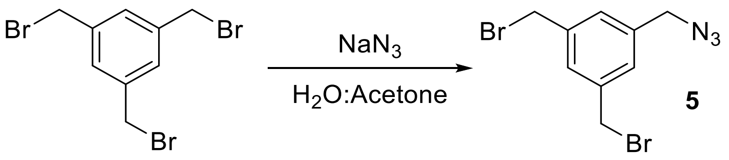

To a solution of 1,3,5-tris(bromomethyl)benzene (1.0 g, 2.8 mmol) in a mixture of acetone:water (6:1, 30 mL) was added sodium azide (0.18 g, 2.8 mmol) portionwise over 5 min. The reaction mixture was stirred for 3.5 h at room temperature, and then diluted with DCM (10 mL). The organics were washed with H_2O (3 × 10 mL) and brine (10 mL), dried over Na_2SO_4, filtered, and concentrated *in vacuo*. The residue was purified by flash chromatography using 10% DCM:Petrol. Pure fractions were concentrated *in vacuo* to afford the product **5** as an oil (220 mg, 0.79 mmol, 28%). Data were consistent with those previously reported.[2]

^{1}H NMR (400 MHz, $CDCl_3$): δ 7.38 (1H, d, *J* = 1.7 Hz, ArH2), 7.27 (2H, d, *J* = 1.7 Hz, ArH4), 4.46 (4H, s, -CH_2Br), 4.36 (2H, s, -CH_2N_3). **HRMS** (APCI): *m/z* calcd for

$C_9H_{10}Br_2N$: 289.9175[M+H]$^+$; found: 289.9167 (Compound ionised as the amine, rather than azide)

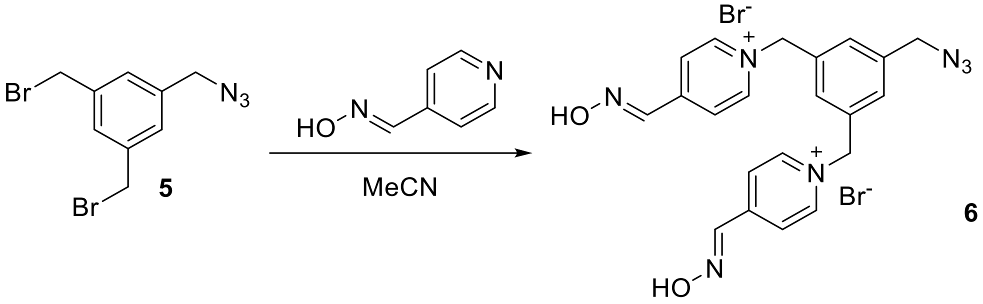

5 (400 mg, 1.13 mmol) and pyridine-4-aldoxime (703 mg, 5.76 mmol) were dissolved in MeCN (10 mL) and the mixture heated to 65 °C for 32 h. The mixture was cooled to room temperature and the precipitate recovered by filtration. The precipitate was washed with MeCN (5 mL) and dried under vacuum. The residue was then purified by reverse-phase column chromatography using a gradient of 0-100% MeCN:Water. Pure fractions were concentrated *in vacuo* to afford the product **6** as a yellow solid (450 mg, 0.80 mmol, 71%).

^{1}H NMR (400 MHz, D_2O): δ 8.88-8.76 (4H, m, Pyr H2), 8.36 (2H, d, *J* = 1.3 Hz, HC=NOH), 8.19 (4H, dd, *J* = 7.1, 1.3 Hz, Pyr H3), 7.53 (2H, d, *J* = 1.7 Hz, Phe H2), 7.49 (1H, d, *J* = 1.7 Hz, Phe H4), 5.84 (4H, s, 4H, -CH_2N$^+$), 4.46 (2H, s, -CH_2N$_3$); **^{13}C NMR** (101 MHz, D_2O): δ 149.48 (Pyr C4), 149.34 (Pyr C4), 146.21 (C=NOH), 146.15 (C=NOH), 144.81 (Pyr C3), 144.70 (Pyr C3), 135.64 (Phe C3), 134.77 (Phe C1), 130.29 (Phe C2), 129.98 (Phe C2), 129.06 (Phe C4), 125.08 (Pyr C2), 63.38 (-CH_2N^+), 63.05 (-CH_2N^+), 53.29 (-CH_2N_3); **HRMS** (ESI): *m/z* calcd for $C_{21}H_{20}N_7O_2$: 402.1673 [M+H]$^+$; found: 402.1668.

2. NASA synthesis

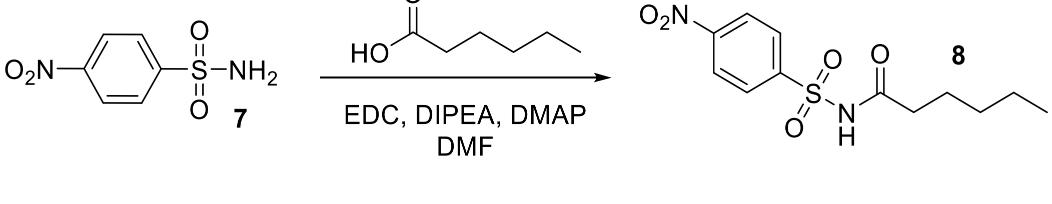

To a solution of 4-nitrobenzenesulfonamide **7** (101 mg, 0.50 mmol) in DMF (10 mL) was added hexanoic acid (95 µL, 0.75 mmol), EDC (300 mg, 1.5 mmol), 4-

dimethylaminopyridine (30 mg, 0.25 mmol) and DIPEA (260 μL, 1.5 mmol). The mixture was allowed to stir at room temperature for 40 h. The solvent was then removed *in vacuo*, and the residue purified by flash chromatography using 5-30% EtOAc:Petrol. Pure fractions were concentrated *in vacuo* to afford the product **8** as a white solid (140 mg, 0.45 mmol, 90%). Data were consistent with those previously reported.[3]

^{1}H NMR (400 MHz, $CDCl_3$) δ: 8.35 (2H, d, *J* = 9.0 Hz, ArH3), 8.25 (2H, d, *J* = 9.0 Hz, ArH2), 2.27 (2H, t, *J* = 7.4 Hz, -$COCH_2$), 1.59-1.50 (3H, m, -$COCH_2CH_2$ & -$COCH_2CH_2CH_2$), 0.94-0.85 (3H, m, -$COCH_2CH_2CH_2$ & -CH_2CH_3), 0.82 (3H, t, *J* = 6.9 Hz, -CH_3). **HRMS** (ESI^-): *m/z* calcd. for $C_{12}H_{15}N_2O_5S$: 299.0707 [M-H]; found: 299.0707.

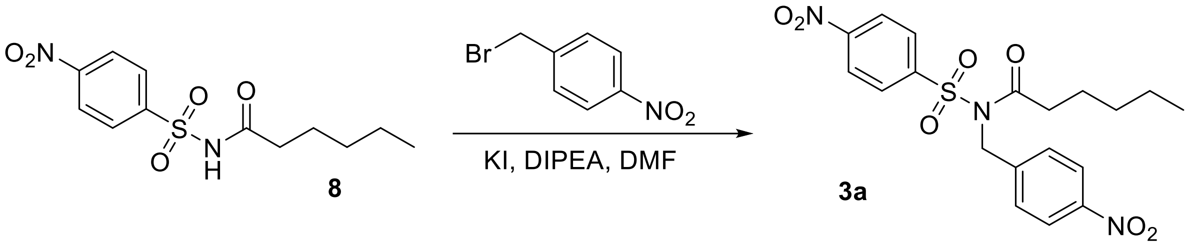

8 **3a**

To a solution of *N*-((4-nitrophenyl)sulfonyl)hexanamide **8** (70 mg, 0.23 mmol) in DMF (2 mL) was added 4-nitrobenzyl bromide (151 mg, 0.7 mmol), potassium iodide (115 mg, 0.7 mmol) and DIPEA (122 μL, 0.7 mmol). The mixture was allowed to stir at room temperature for 48 h. The solvent was then removed *in vacuo*, and the residue purified by flash chromatography using 10-100% EtOAc:Petrol. Pure fractions were concentrated *in vacuo* to afford the product **3a** as a pale pink solid (39 mg, 89 μmol, 39%).

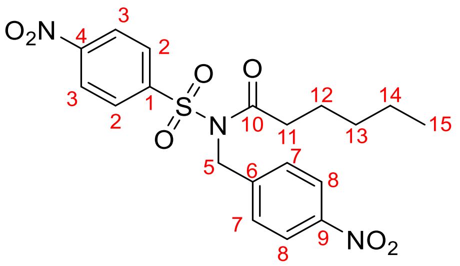

^{1}H NMR (500 MHz, $CDCl_3$): δ 8.38 (2H, d, *J* = 8.9 Hz, -H3), 8.23 (2H, d, *J* = 8.7 Hz, -H8), 8.06 (2H, d, *J* = 8.9 Hz, -H2), 7.55 (2H, d, *J* = 8.7 Hz, -H7), 5.16 (2H, s, -H5), 2.48

(2H, t, J = 7.4 Hz, -H11), 1.49 (2H, tt, $J_1 = J_2$ = 7.4 Hz, -H12), 1.23-1.06 (4H, m, -H13 and H14), 0.84 (3H, t, J = 7.4 Hz, -H15). **^{13}C NMR** (125 MHz, $CDCl_3$): δ 172.72 (C10), 150.70 (C1), 147.65 (C6), 144.66 (C4), 143.34 (C9), 129.25 (C2), 128.11 (C7), 124.42 (C3), 124.06 (C8), 49.26 (C5), 36.19 (C11), 30.86 (C13), 23.91 (C12), 22.18 (C14), 13.67 (C15); **HRMS** (ESI$^-$): *m/z* calcd. for $C_{19}H_{21}N_3O_7S$: 434.1027 [M-H]; found: 434.1019.

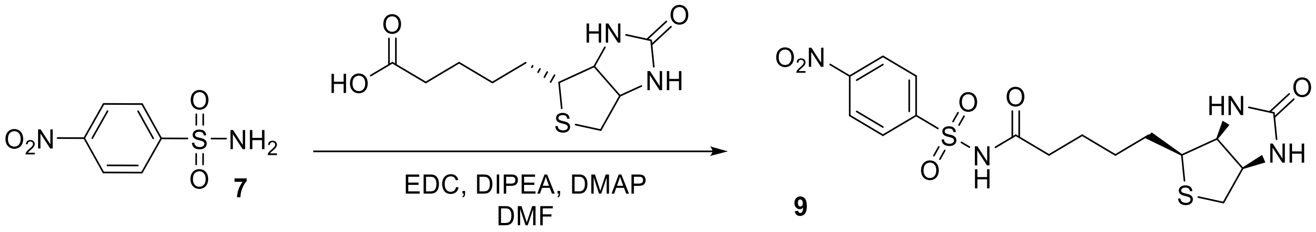

To a solution of 4-nitrobenzenesulfonamide **7** (376 mg, 1.86 mmol) in DMF (12 mL) was added biotin (502 mg, 2.05 mmol), EDC (720 mg, 3.75 mmol), 4-dimethylaminopyridine (DMAP) (458 mg, 3.75 mmol) and DIPEA (1.0 mL, 5.58 mmol). The mixture was allowed to stir at room temperature for 18 h. The solvent was then removed under reduced pressure, and the residue purified by flash chromatography using 0.25% AcOH, 2.5% MeOH:DCM. Pure fractions were concentrated under reduced pressure to afford the product **9** as a white solid (540 mg, 1.05 mmol, 68%). Data were consistent with those previously reported.[4]

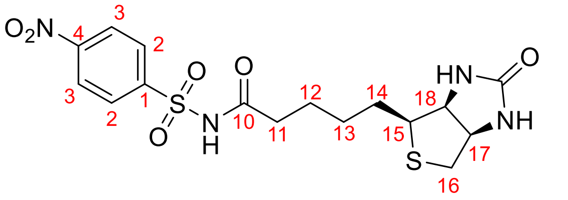

^{1}H NMR (400 MHz, DMSO-d_6): δ 8.39 (2H, d, J = 7.2 Hz, -H3), 8.11 (2H, d, J = 7.2 Hz, -H2), 6.34 (1H, s, -NH biotin), 6.32 (1H, s, -NH biotin), 4.27-4.23 (1H, m, -H17 or 18), 4.06-4.03 (1H, m, -H17 or 18), 3.01-2.96 (1H, m, -H15), 2.76 (1H, dd, J = 12.0, 5.2 Hz, -H16), 2.52 (1H, d, J = 12.0 Hz, -H16), 2.18 (2H, t, J = 7.4 Hz, -H11), 1.52-1.32 (4H, m, -H12 and -H14), 1.20-1.14 (2H, m, -H13). **HRMS** (ESI$^-$): *m/z* calcd for $C_{16}H_{20}N_4NaO_6S_2$: 451.0716 [M-H]; found: 451.0723

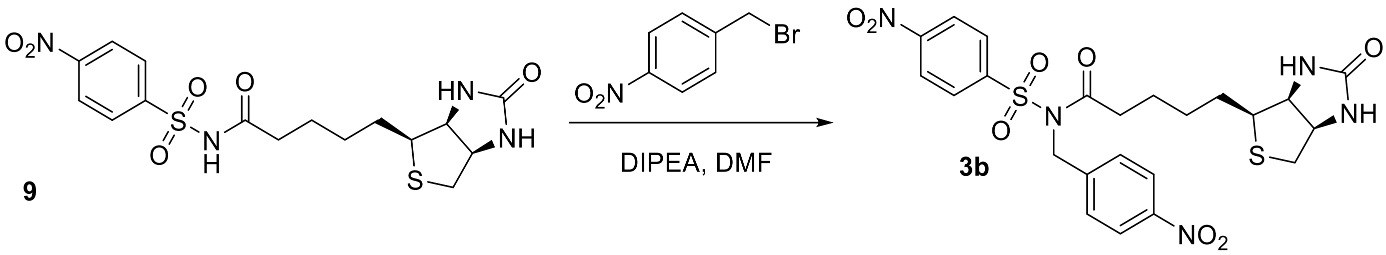

To a solution of compound **9** (90 mg, 0.21 mmol) in dry DMF (3.0 mL) was added 4-nitrobenzyl bromide (272 mg, 1.27 mmol) and DIPEA (100 µL, 0.57 mmol). The mixture was allowed to stir at room temperature for 48 h. The solvent was then removed under reduced pressure, and the residue purified by flash chromatography using 1-10% MeOH:DCM. Pure fractions were concentrated under reduced pressure to afford the product **3b** (60 mg, 0.11 mmol, 51%) as a white solid. Data were consistent with those previously reported.[4]

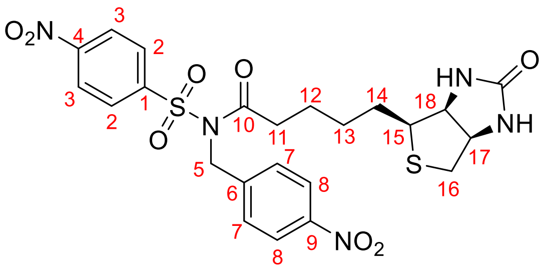

^{1}H NMR (400 MHz, $CDCl_3$): δ 8.41 (2H, d, J = 8.8 Hz, -H3), 8.25 (2H, d, J = 8.8 Hz, H8), 8.10 (2H, d, J = 8.8 Hz, -H2), 7.56 (2H, d, J = 8.8 Hz, -H7), 5.18 (2H, s, -H5), 4.50-4.47 (1H, m, -H17 or 18), 4.25-4.22 (1H, m, -H17 or 18), 3.07-3.02 (1H, m, -H15), 2.87 (1H, dd, J = 13.0, 5.2 Hz, -H16), 2.68 (1H, d, J = 13.0 Hz, -H16), 2.54 (2H, t, J = 7.0 Hz, -H11), 1.61-1.43 (4H, m, -H12 and -H14), 1.32-1.23 (2H, m, -H13). **HRMS** (ESI^{+}): *m/z* calcd for $C_{23}H_{25}N_5NaO_8S_2$: 586.1037 [M+Na]; found: 586.1051

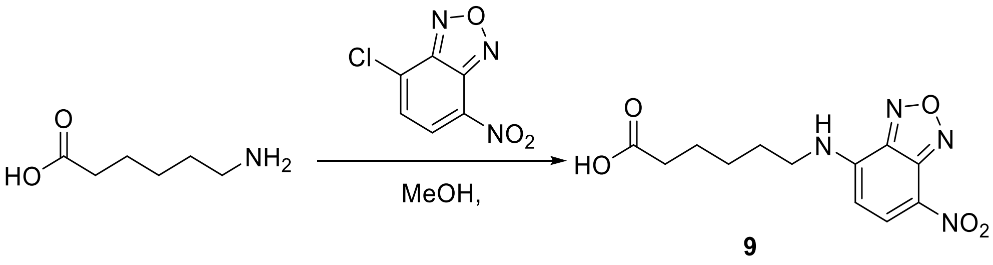

4-chloro-7-nitrobenzo-2-oxa-1,3-diaziole (924 mg, 4.6 mmol) and $NaHCO_3$ (1.16 g, 13.8 mmol) were added to a solution of 6-aminohexanoic acid (602 mg, 4.6 mmol) in methanol (32 mL) in an amber flask for light exclusion. The reaction mixture was stirred

under light exclusion at 50 °C for 3.5 h. After cooling to room temperature, the reaction was concentrated *in vacuo* and the residue diluted with hydrochloric acid (0.5 M, 30 mL) and extracted with ethyl acetate (3 × 30 mL). The organic layers were combined, dried over anhydrous $MgSO_4$ and concentrated under reduced pressure to afford **9** (1.25 g, 4.3 mmol, 93%) as a brown solid. Data were consistent with those previously reported.[5]

^{1}H NMR (400 MHz, DMSO-d_6): δ 9.55 (1H, app s, -NH), 8.50 (1H, d, J = 9.0 Hz, ArH), 6.40 (1H, d, J = 9.0 Hz, ArH), 3.46 (2H, t, J = 6.9 Hz, 2H, -C<u>H</u>$_2$NH-), 2.21 (2H, t, J = 7.3 Hz, -C<u>H</u>$_2$CO$_2$H), 1.68 (2H, tt, J_1 = J_2 = 7.4 Hz, -C<u>H</u>$_2$CH$_2$NH-), 1.54 (2H, tt, J_1 = J_2 = 7.4 Hz, -C<u>H</u>$_2$CH$_2$CO$_2$H), 1.37 (2H, tt, J_1 = J_2 = 7.4 Hz, -C<u>H</u>$_2$CH$_2$ CH$_2$NH-); **HRMS** (ESI$^+$): *m/z* calcd for $C_{12}H_{13}N_4O_5$: 293.0891 [M+H]$^+$; found: 293.0892.

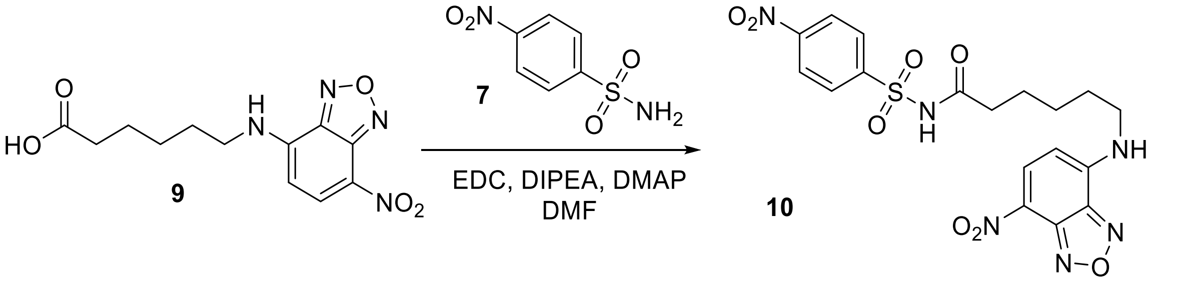

To a solution of 4-nitrobenzenesulfonamide **7** (101 mg, 0.50 mmol) in DMF (5 mL) was added NBD **9** (219 mg, 0.75 mmol), EDC (300 mg, 1.5 mmol), 4-dimethylaminopyridine (33 mg, 0.25 mmol) and DIPEA (260 µL, 1.5 mmol). The mixture was allowed to stir at room temperature for 40 h. The solvent was then removed *in vacuo*, and the residue purified by flash chromatography using 0.25-5% MeOH:DCM. Pure fractions were concentrated *in vacuo* to afford the product **10** as an orange solid (36 mg, 0.07 mmol, 14%). The product was used in subsequent steps without further analysis.

^{1}H NMR (400 MHz, DMSO-d_6): δ 8.50 (1H, d, J = 9.0 Hz, NBD ArH), 8.46-8.38 (2H, m, PhH3), 8.17-8.12 (2H, m, PhH4), 6.37 (1H, d, J = 9.0 Hz, NBD ArH), 3.45 (2H, td, J_1 = J_2 = 6.8 Hz, -C<u>H</u>$_2$NH), 2.24 (2H, t, J = 7.3 Hz, -C<u>H</u>$_2$CONHR), 1.60 (2H, tt, J_1 = J_2 = 7.3 Hz, -C<u>H</u>$_2$CH$_2$NH), 1.45 (2H, tt, J_1 = J_2 = 7.3 Hz, -C<u>H</u>$_2$CH$_2$CONHR), 1.29-1.21 (2H, m, -C<u>H</u>$_2$CH$_2$CH$_2$CONHR); **HRMS** (ESI$^+$): *m/z* calcd for $C_{18}H_{17}N_6O_8S$: 477.0834 [M+H]; found: 477.0840.

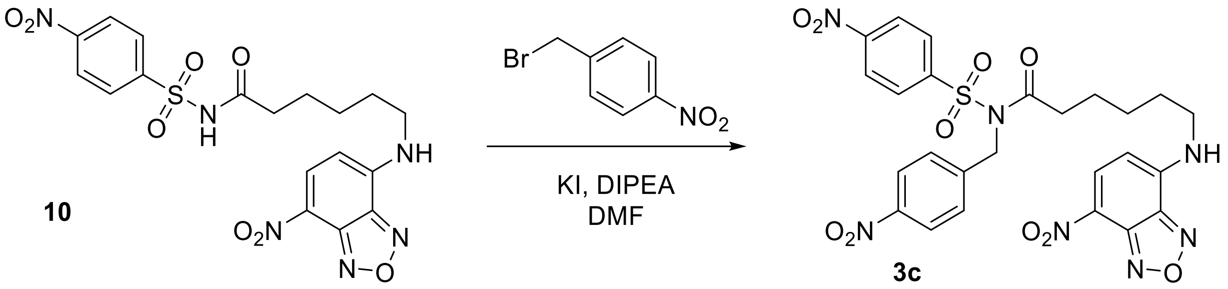

To a solution of **10** (36 mg, 0.075 mmol) in dry DMF (2.0 mL) was added 4-nitrobenzyl bromide (98 mg, 0.45 mmol), potassium iodide (46 mg, 0.22 mmol) and DIPEA (40 µL, 0.22 mmol). The mixture was allowed to stir at room temperature for 30 h. The solvent was then removed *in vacuo*, and the residue purified by flash chromatography using 0-0.5% MeOH:DCM. Pure fractions were concentrated *in vacuo* to afford the product **3c** as an orange solid (10 mg, 16 µmol, 22%).

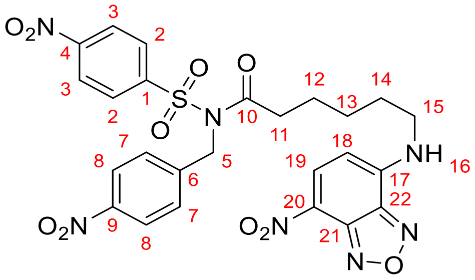

^{1}H NMR (500 MHz, $CDCl_3$): δ 8.47 (1H, d, J = 8.5 Hz, 1H, H18), 8.40 (2H, d, J = 8.5 Hz, H3), 8.24 (2H, d, J = 8.2 Hz, H8), 8.08 (2H, d, J = 8.5 Hz, H2), 7.56 (2H, d, J = 8.2 Hz, 2H, H7), 6.21 (1H, app s, H16), 6.13 (1H, d, J = 8.5 Hz, H19), 5.15 (2H, s, H5), 3.45 (2H, td, J_1 = J_2 = 6.8 Hz, 2H, H15), 2.59 (2H, t, J = 7.0 Hz, H11), 1.76 (2H, tt, J_1 = J_2 = 7.0 Hz, H14), 1.65 (2H, tt, J_1 = J_2 = 7.0 Hz, H12), 1.39 (2H, tt, J_1 = J_2 = 7.0 Hz, H13); **^{13}C NMR** (126 MHz, Chloroform-*d*): δ 207.28 (C17), 172.92 (C10), 150.94 (C1), 147.86 (C6), 144.59 (C21/22), 144.35 (C21/22), 143.65 (C4), 143.15 (C9), 136.37 (C19), 129.31 (C2), 128.37 (C7), 124.69 (C3), 124.24 (C8), 98.41 (C18), 48.64 (C5), 43.48 (C15), 36.16 (C11), 28.20 (C14), 26.78 (C12), 23.42 (C13); **HRMS** (ESI$^+$): *m/z* calcd for $C_{25}H_{22}N_7O_{10}S$: 612.1154 [M+H]; found: 612.1145.

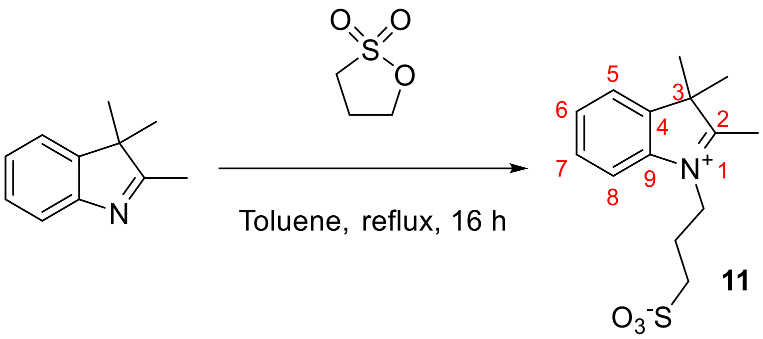

11

A mixture of 2,3,3-trimethylindolenine (2.00 mL, 12.7 mmol) and 1,3-propanesultone (1.55 g, 12.7 mmol) in toluene (50 mL) was refluxed for 20 h, during which time a dark red precipitate formed. After cooling to r.t., the reaction mixture was concentrated under reduced pressure. The residue was redissolved in dichloromethane (5 mL) and the solution added dropwise to diethyl ether (200 mL). The resultant precipitate was collected by filtration, washed with diethyl ether (50 mL), and dried in air to yield a red oil (3.10 g, 11.0 mmol, 87%). Data were consistent with those previously reported.[6]

^{1}H NMR (400 MHz, CD_3OD) δ 8.01-7.93 (m, 1H, $\underline{H}5$), 7.78-7.70 (m, 1H, $\underline{H}7$), 7.68-7.59 (m, 2H, $\underline{H}6$, $\underline{H}8$), 4.78-4.67 (m, 2H, $PhC\underline{H}_2$), 3.03-2.93 (m, 2H, $C\underline{H}_2SO_3^-$), 2.43-2.26 (m, 2H, $C\underline{H}_2CH_2SO_3^-$), 1.58 (s, 6H, 2 × $C\underline{H}_3$).

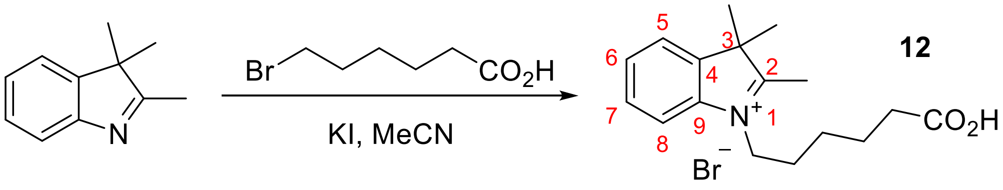

12

A mixture of 2,3,3-trimethylindolenine (2.00 mL, 12.7 mmol), 6-bromohexanoic acid (3.16 g, 16.2 mmol), and potassium iodide (2.69 g, 16.2 mmol) in acetonitrile (20 mL) was refluxed for 20 h. After cooling to r.t., the reaction mixture was added dropwise to diethyl ether (200 mL). The resultant precipitate was collected by filtration, washed with diethyl ether (50 mL), and dried in air to yield a red solid (4.38 g, 11.0 mmol, 87%). Data were consistent with those previously reported.[7]

^{1}H NMR (400 MHz, DMSO) δ 7.91-7.97 (1H, m, $\underline{H}5$), 7.76-7.784 (1H, m, $\underline{H}7$), 7.53-7.63(2H, m, $\underline{H}6$, $\underline{H}8$), 4.41 (2H, t, J = 7.7 Hz, $-CH_2N-$), 2.18 (2H, t, J = 7.2 Hz, $-C\underline{H}_2CO_2H$), 1.80 (2H, tt, J_1 = J_2 = 7.7 Hz, $-C\underline{H}_2CH_2N-$), 1.39-1.55 (10H, $-C\underline{H}_2C\underline{H}_2CH_2CO_2H$, 2 × $C\underline{H}_3$).

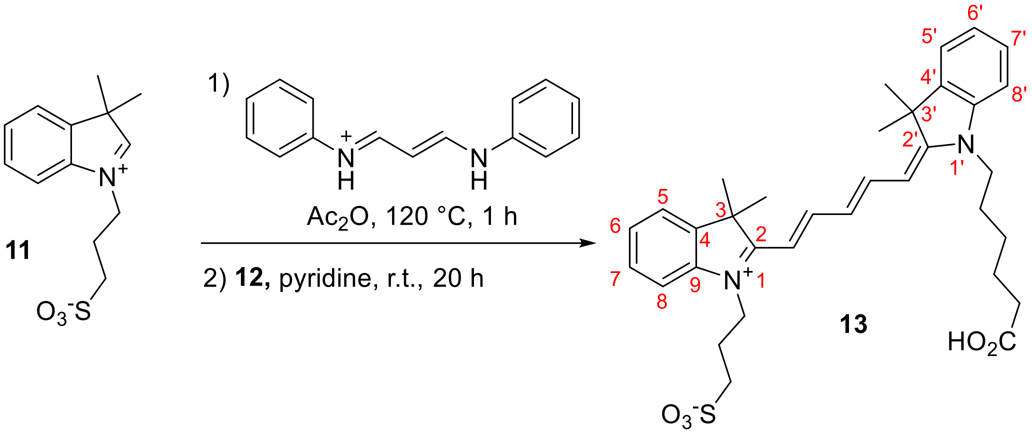

A mixture of **11** (0.5 g, 1.8 mmol) and malonaldehyde bis(phenylimine) monohydrochloride (0.47 g, 1.8 mmol) in acetic anhydride (4 mL) was heated to 120 °C for 1.5 h. After cooling to r.t, a solution of **12** (0.94 g, 2.3 mmol) in pyridine (4 mL) was added and stirring was continued at r.t. for a further 16 h. The reaction mixture was then added dropwise to diethyl ether (200 mL), and the resultant precipitate collected by filtration, washed with diethyl ether (30 mL), and dried under vacuum. The solid was then redissolved in methanol (10 mL) and concentrated under reduced pressure. The residue was purified via flash column chromatography eluting with 5-9% MeOH:DCM. Fractions containing the product were concentrated under reduced pressure to provide a blue solid (0.71 g, 1.22 mmol, 68%). Data were consistent with those previously reported.[7]

^{1}H NMR (400 MHz, DMSO-d_6) δ 8.31 (dd, $J_1 = J_2$ = 13.0 Hz, 2H, 2 × CHCHCN), 7.30-7.64 (6H, m, H5, H8**,** H7), 7.15-7.23 (2H, m, H6), 6.52 (1H, dd, $J_1 = J_2$ = 13.0 Hz, CHCHCHCN), 6.40 (1H, d, J = 13.0 Hz, CHCN), 6.27 (1H, d, J = 13.0 Hz, CHCN), 4.18-4.28 (2H, m, -CH_2N), 4.05 (2H, t, J = 7.2 Hz, -CH_2N), 2.51-2.58 (2H, m, $CH_2SO_3^-$), 2.16 (2H, t, J = 7.2 Hz, -CH_2CO_2H), 1.89-2.02 (4H, m, -CH_2-), 1.63 (s, 12H, CyCH_3), 1.45-1.54 (2H, m, -CH_2-), 1.26-1.35 (2H, m, -CH_2-).

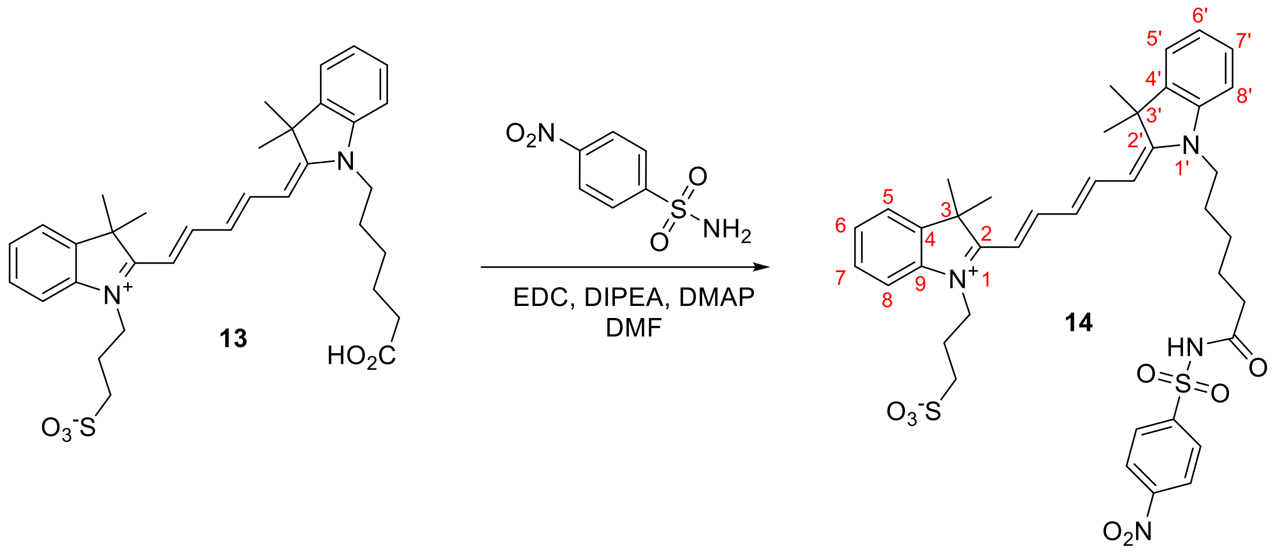

To a solution of 4-nitrobenzenesulfonamide (35 mg, 0.17 mmol) in DMF (5 mL) was added Cy5-CO_2H **13** (110 mg, 0.19 mmol), EDC (67 mg, 0.35 mmol), 4-dimethylaminopyridine (52 mg, 0.35 mmol) and DIPEA (92 µL, 0.53 mmol). The mixture was stirred for 20 h and then concentrated *in vacuo*. The residue was purified by flash chromatography eluting with 1-10% MeOH:DCM. Pure fractions were concentrated *in vacuo* to afford the product **14** as an orange solid (36 mg, 0.07 mmol, 14%). The product was used in subsequent steps without further analysis.

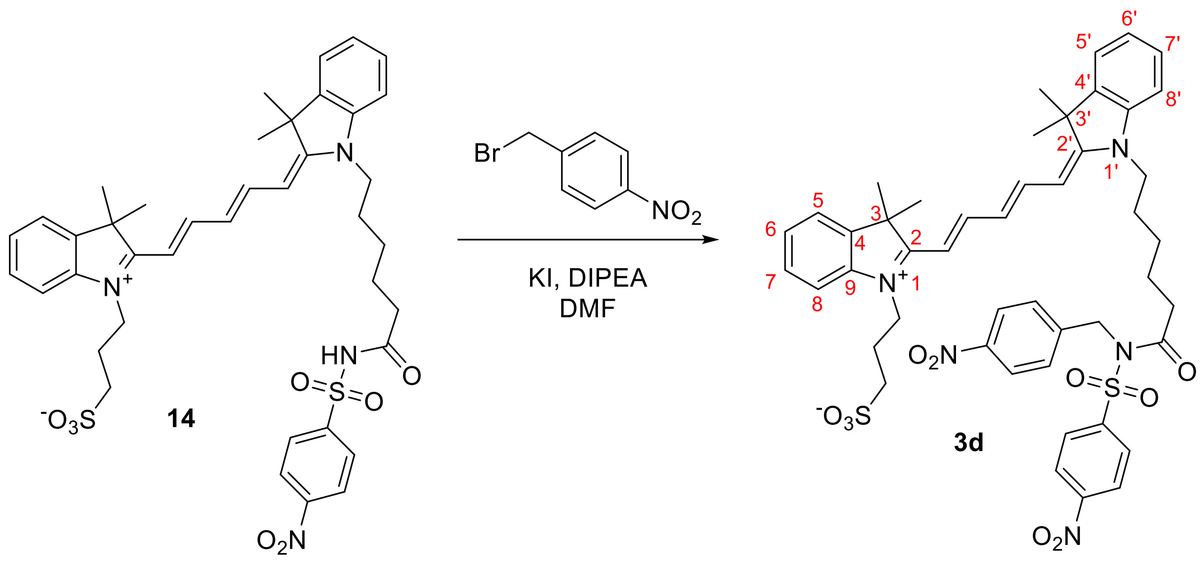

To a solution of **14** (36 mg, 0.07 mmol) in dry DMF (2.0 mL) was added 4-nitrobenzyl bromide (98 mg, 0.45 mmol), potassium iodide (46 mg, 0.22 mmol) and DIPEA (40 µL, 0.22 mmol). The mixture was allowed to stir at room temperature for 30 h. The solvent

was then removed *in vacuo*, and the residue purified by flash chromatography using 1-10% MeOH:DCM. Pure fractions were concentrated *in vacuo* to afford the product **3d** as a blue solid (9 mg, 10 µmol, 14%).

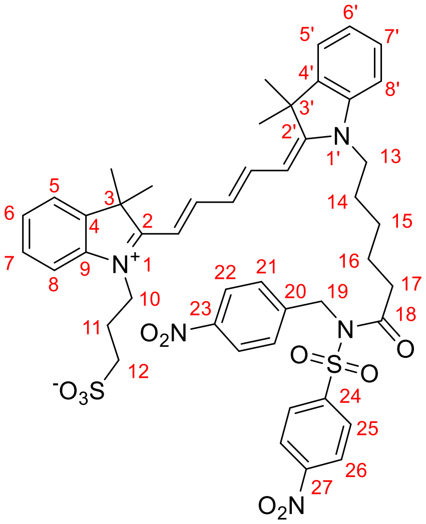

^{1}H NMR (400 MHz, DMSO-d_6) δ 8.41-8.45 (2H, m, H25), 8.22-8.37 (6H, m, H26, H25, & 2 × CHCHCN), 7.19-7.63 (10H, m, H21, H5, H6, H7, & H8), 6.44-6.52 (2H, m, CHCN & CHCHCHCN), 6.21 (1H, d, *J* = 13.7 Hz, CHCHCHCN), 5.31 (2H, s, H19), 4.29 (2H, t, *J* = 7.6 Hz, H10/13), 4.01 (2H, t, *J* = 6.8 Hz, H10/13), 2.58 (2H, t, *J* = 6.7 Hz, H12), 1.97-2.04 (2H, m, H17), 1.67 (6H, s, CyCH_3), 1.63 (6H, s, CyCH_3), 1.55 (2H, t, *J* = 7.5 Hz, H14-16), 1.46 (2H, t, *J* = 7.3 Hz, H14-16), 1.23-1.28 (2H, m, H14-16); **^{13}C NMR** (101 MHz, DMSO-d_6) δ 173.1, 172.3, 162.7, 154.3, 153.9, 150.5, 146.9, 144.9, 144.0, 142.1, 142.0, 141.1, 141.1, 129.7, 128.5, 128.3, 127.5, 125.6, 124.8, 124.6, 124.5, 123.9, 122.4, 122.4, 111.2, 110.9, 103.6, 102.9, 49.3, 49.0, 48.8, 47.8, 43.0, 42.7, 34.7, 27.1, 27.1, 26.5, 25.1, 23.4, 23.2; **HRMS** (ESI$^+$): *m/z* calcd for $C_{47}H_{51}N_5NaO_{10}S_2$: 932.2975 [M+Na]; found: 932.3040; (FT-ATR)/cm^{-1}: 1705, 1479, 1447, 1373, 1333, 1169, 1130, 1083, 1031, 1012; **m.p**: 180-186 °C.

3. Peptide synthesis

Solid-phase peptide synthesis (SPPS) was performed on a CEM Liberty Lite Automated Microwave Peptide Synthesiser, according to the manufacturer's standard protocols. Briefly, Fmoc-protected amino acids (5 equiv., 0.2 M in DMF) were coupled in the presence of *N,N'*-diisopropylcarbodiimide (DIC, 15 equiv.) and Oxyma Pure (5 equiv.), as coupling agent and base respectively, under microwave irradiation at a

temperature of 90 °C for 2 minutes. Arginine and cysteine were coupled at 25 °C and 50 °C for 10 minutes respectively. Where required, the unnatural amino acids 4-(Fmoc-amino)butyric acid (Fmoc-GABA-OH), Fmoc-Lys(Dde)-OH, or Fmoc-propargylglycine-OH were installed as handles for peptide functionalisation. 5-pentynoic acid was similarly installed under standard coupling conditions where required. Fmoc deprotection was performed using 20% piperidine in DMF at 90 °C for 60 seconds. Syntheses were performed on a 0.1 mmol scale, using Rink Amide MBHA resin (*C*-terminal amide, 0.5 mmol/g, 1% DVB, 100-200 mesh, Fluorochem) unless otherwise specified. Prior to cleavage, the resin was washed sequentially with DCM (3 × 15 mL) and methanol (3 × 15 mL).

Peptides were cleaved from the resin in 20 mL of cleavage cocktail (90% TFA, 5% H_2O, 3% TIPS, 2% DTT for Cys-containing sequences) for 4 hrs (18 hrs for Arg-containing sequences). After filtration, the resin was washed extensively with DCM (3 × 50 mL) and the filtrate concentrated *in vacuo* to ~2 mL volume. The residue was dropped into ice cold diethyl ether (~ 50 mL), and the resultant precipitate collected by centrifugation (3000 rpm, 5 min), resuspended in diethyl ether, and centrifuged again. The residual solid was allowed to air dry for 10 min, then dissolved in deionized water (10 mL) and dried by lyophilisation.

Peptides were typically pure by LC-MS analysis, and used directly. When required, purification was undertaken via reverse-phase flash column chromatography on a Teledyne CombiFlash NEXTGEN 300+ system, using a Redi*Sep* Rf Gold C18Aq column. The column was pre-equilibrated with water, and the peptide purified using a gradient from 0-100% MeCN:H_2O at a flow rate of 13 mL^{-1} min. The gradient programme was dependent on the peptide purified.

On-resin deprotection Lys(Dde): For peptides bearing pyridinium oximes at the C-terminus, Fmoc-Lys(Dde)-OH was introduced as the first amino acid during peptide synthesis and the N-terminus was kept Fmoc-protected. Dde deprotections were then undertaken on a 0.05 mmol scale:

Hydroxylamine hydrochloride (83 mg, 1.2 mmol) and imidazole (65 mg, 1.0 mmol) were suspended in *N*-methyl-2-pyrrolidone (1.5 mL) and the mixture sonicated till full dissolution. The mixture was then added to the peptide resin (0.05 mmol) suspended in DCM (0.5 mL) and stirred for 17 h. The resin was then extensively washed with DCM (3 × 15 mL) and methanol (3 × 15 mL).

Solution phase CuAAC: Unprotected alkyne-modified peptides (0.05 mmol) and azide-functionalised pyridinium oxime (0.06 mmol) were dissolved in a mixture *tert*-butanol (0.5 mL) and water (0.5 mL). Copper sulfate (0.075 mmol) and sodium ascorbate (0.15 mmol) were then added and the mixture stirred for 2 h. Crude mixtures were then analysed by LC-MS, and if starting peptide remained additional azide-functionalised pyridinium oxime (0.025 mmol) was added and the mixture stirred for a further 2 h. The sample was then centrifuged (3000 rpm, 5 min) and the supernatant passed through a 0.45 µm PES filter. The filtrate was then incubated with DMT-functionalised silica gel (40 mg, ≥0.5 mmol/g, Merck) and incubated for 4 hr. The mixture was then filtered *in vacuo*, washed with *tert*-butanol (1 mL), and the filtrate purified by reverse-phase column chromatography as detailed above.

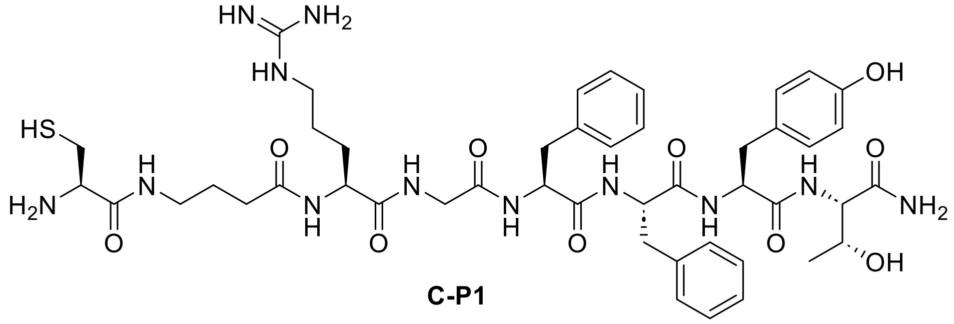

C-P1

HRMS (ESI$^+$): *m/z*
Calcd for $C_{46}H_{66}N_{12}O_{10}S$: 977.4662 $[M+H]^+$; Observed: 977.4647.

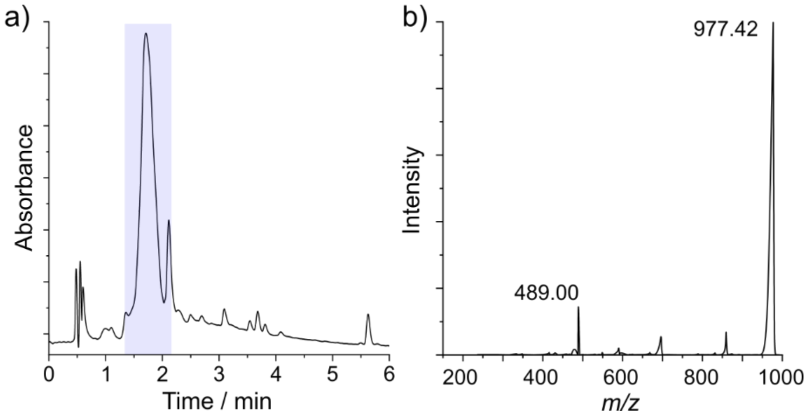

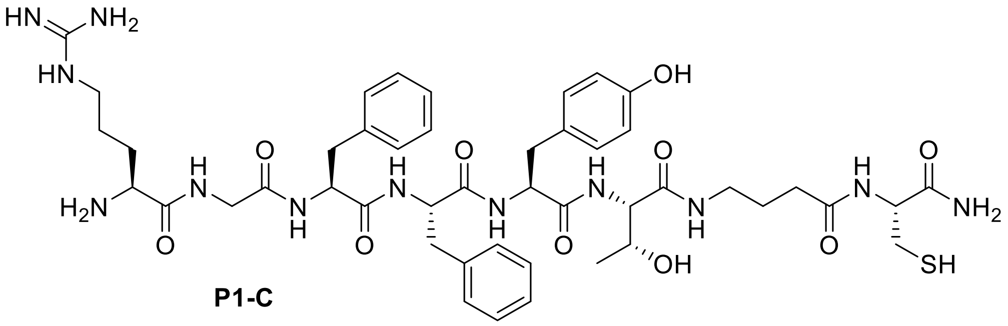

P1-C

HRMS (ESI$^+$): *m/z*

Calcd for $C_{46}H_{66}N_{12}O_{10}S$: 977.4662 [M+H]$^+$; Observed: 977.4655

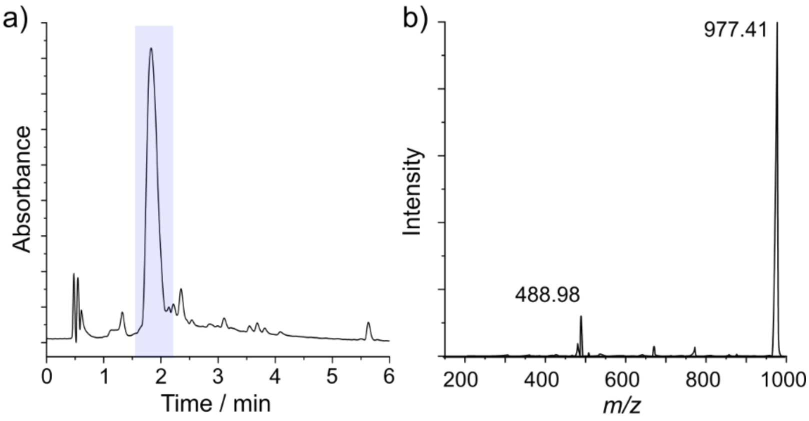

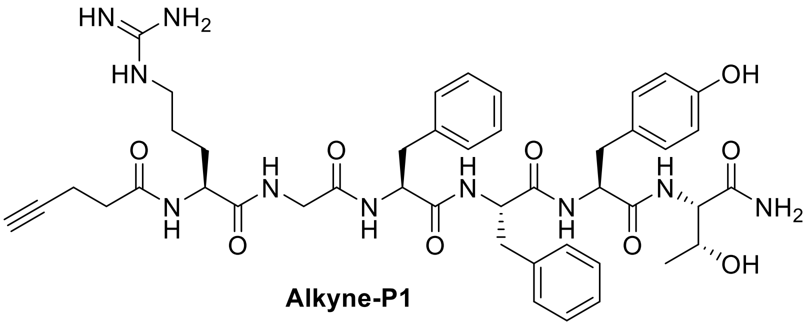

Alkyne-P1

LRMS (ESI$^+$): *m/z* Calcd for $C_{44}H_{56}N_{10}O_9$: 869.43 [M+H]$^+$; Observed: 869.48.

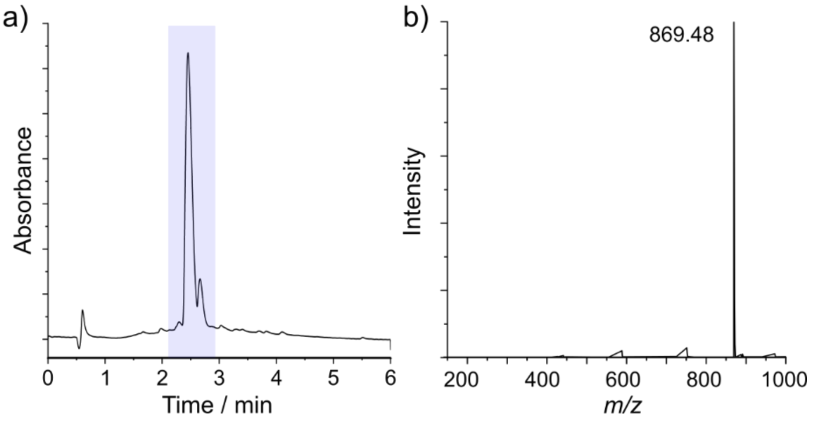

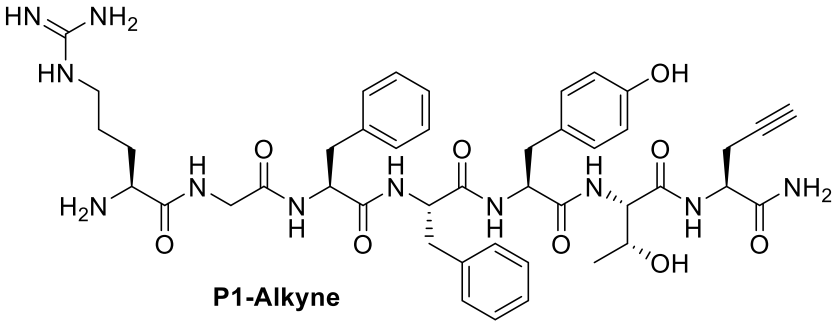

P1-Alkyne

LRMS (ESI+): *m/z* Calcd for $C_{44}H_{57}N_{11}O_9$: 884.44 $[M+H]^+$; Observed: 884.47.

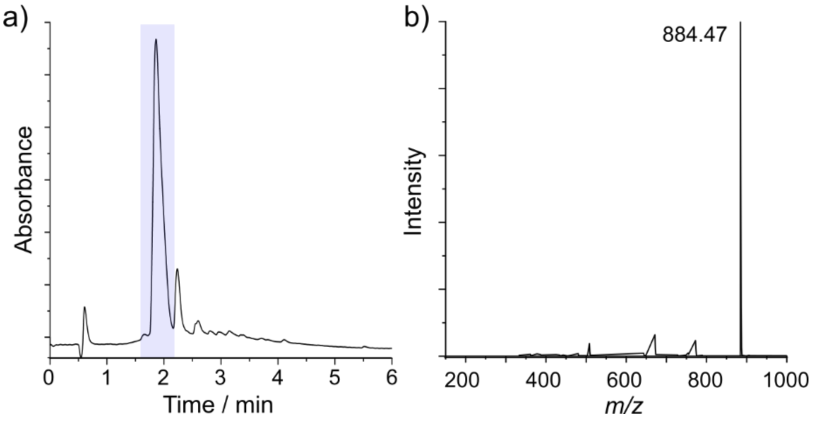

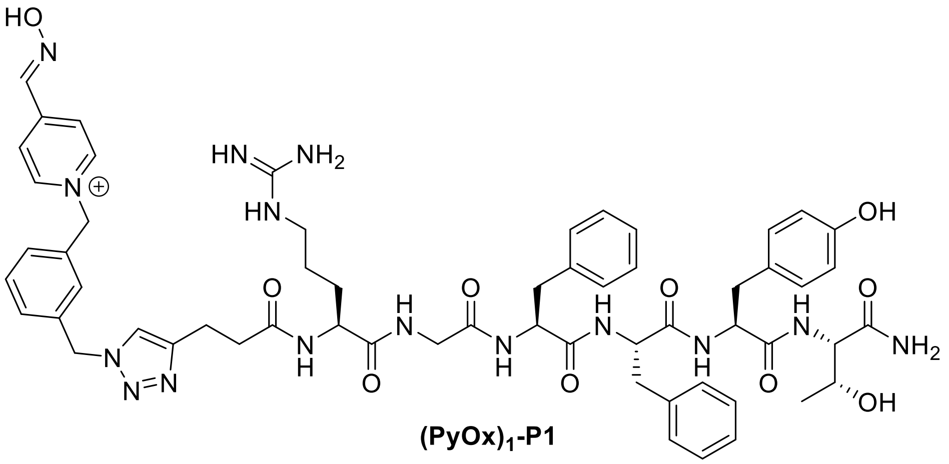

(PyOx)$_1$-P1

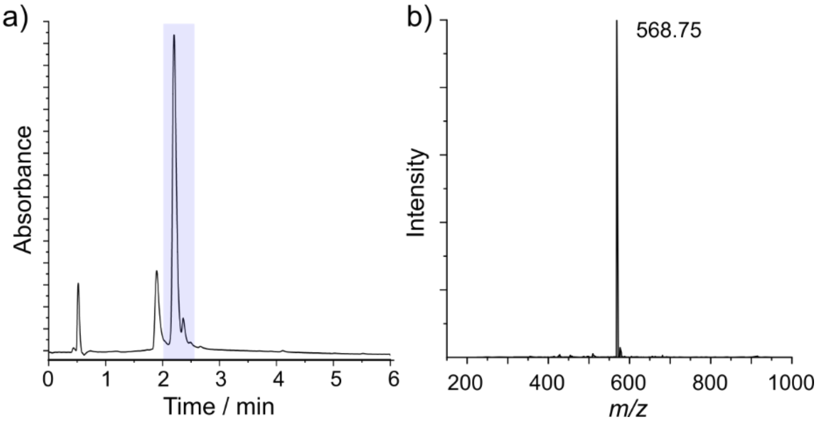

HRMS (ESI$^+$): *m/z*

Calcd for $C_{58}H_{69}N_{15}O_{10}^+$: 568.7749 $[M+2H]^{2+}$;

Observed: 568.7725.

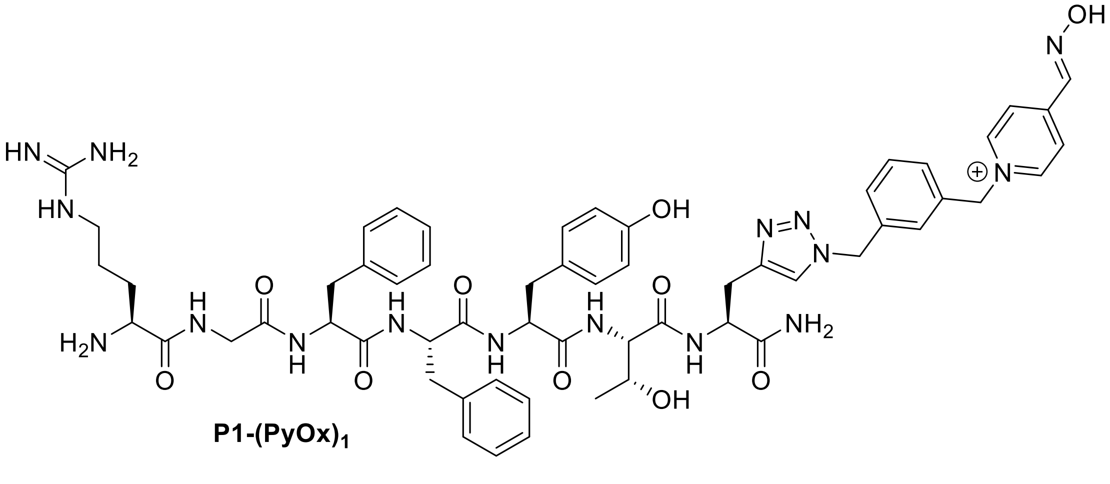

P1-(PyOx)$_1$

HRMS (ESI$^+$): *m/z*

Calcd for $C_{58}H_{71}N_{16}O_{10}$: 576.2803 $[M+2H]^{2+}$; Observed: 576.2735

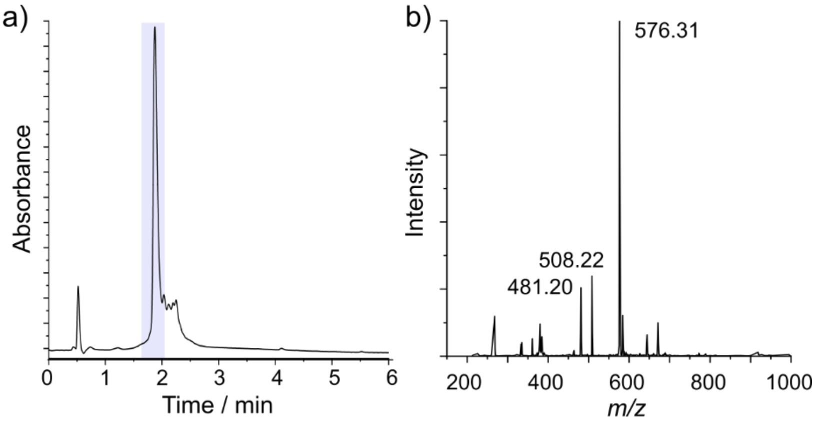

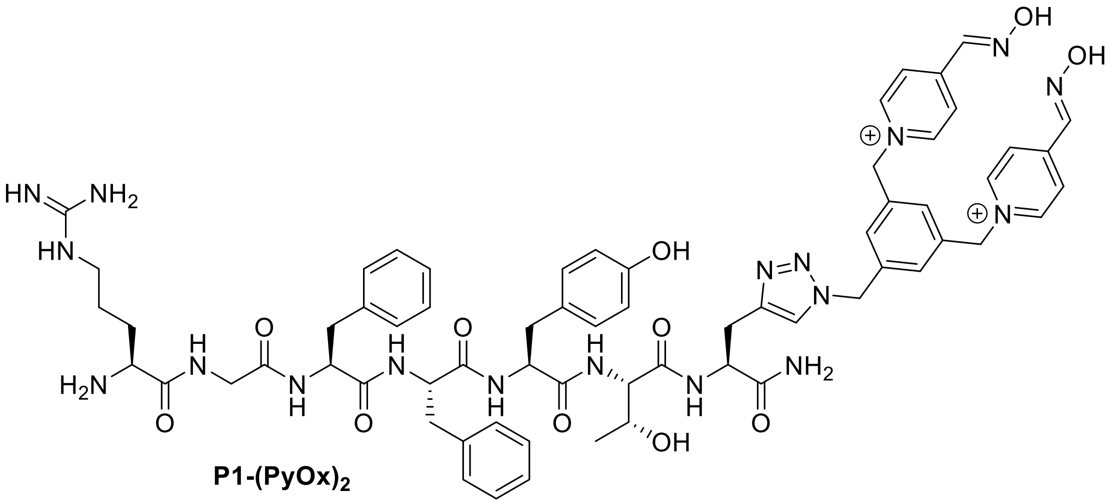

P1-(PyOx)$_2$

HRMS (ESI^+): *m/z* Calcd for $C_{65}H_{78}N_{18}O_{11}^{2+}$: 643.3043 $[M]^{2+}$; Observed: 643.2966.

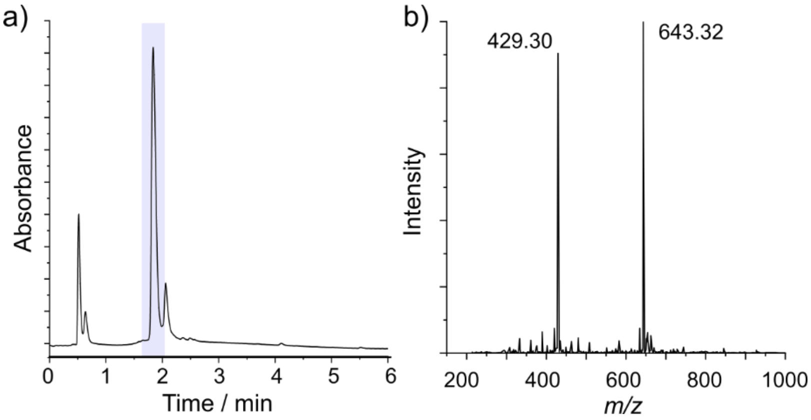

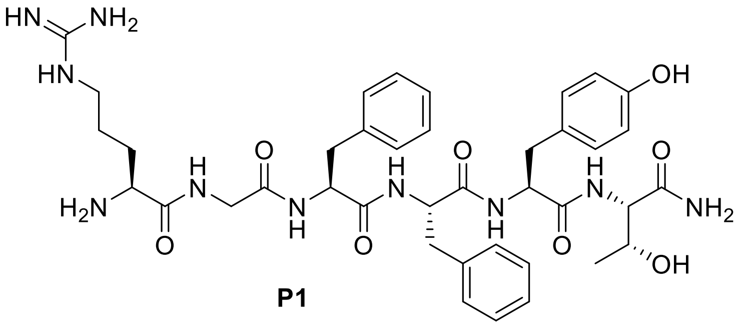

P1

HRMS (ESI$^+$): *m/z*

Calcd for $C_{39}H_{53}N_{10}O_8$: 789.4042 [M+H]$^+$; Observed: 789.4058

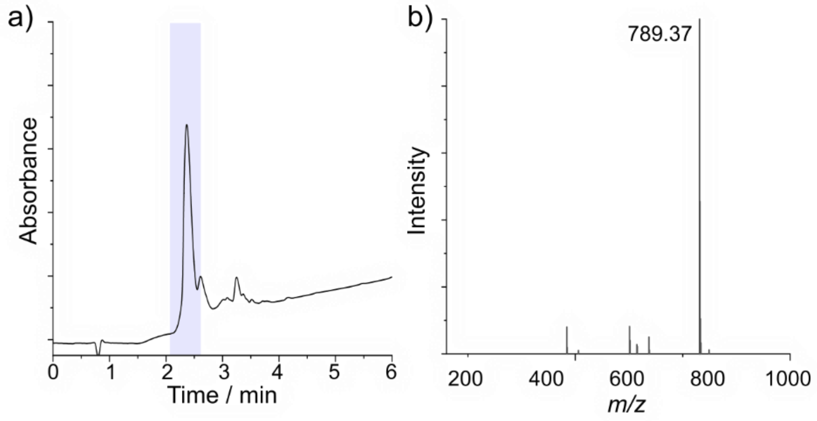

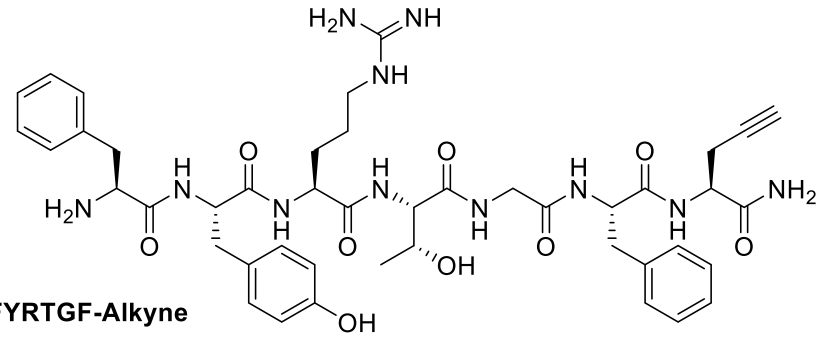

FYRTGF-Alkyne

HRMS (ESI$^+$): *m/z*

Calcd for $C_{44}H_{58}N_{11}O_9$: 884.4413 [M+H]$^+$; Observed: 884.4446

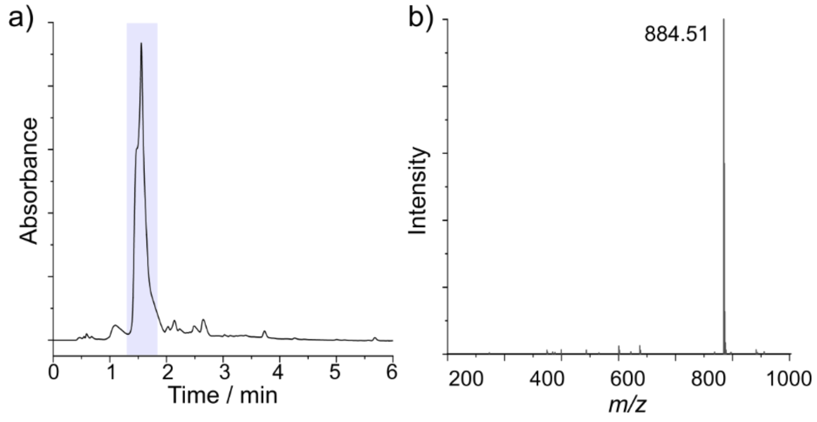

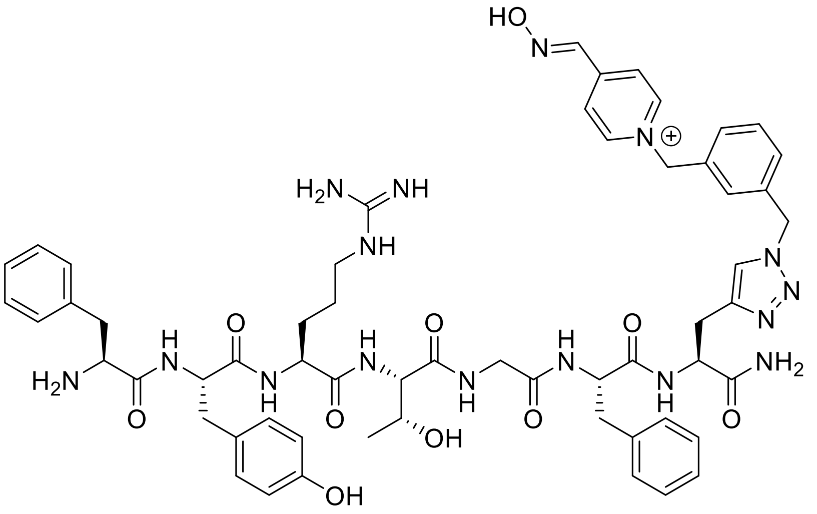

FYRTGF-(PyOx)$_1$

HRMS (ESI$^+$): *m/z*

Calcd for $C_{58}H_{71}N_{16}O_{10}$: 576.2803 [M+2H]$^{2+}$; Observed: 576.2742

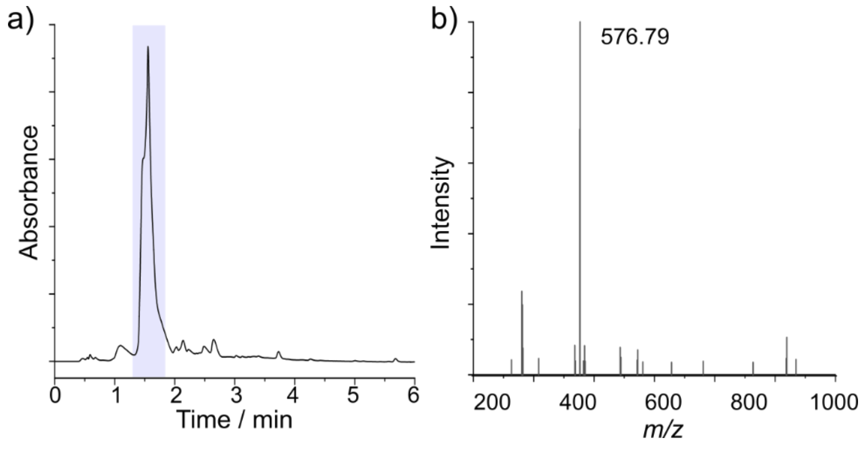

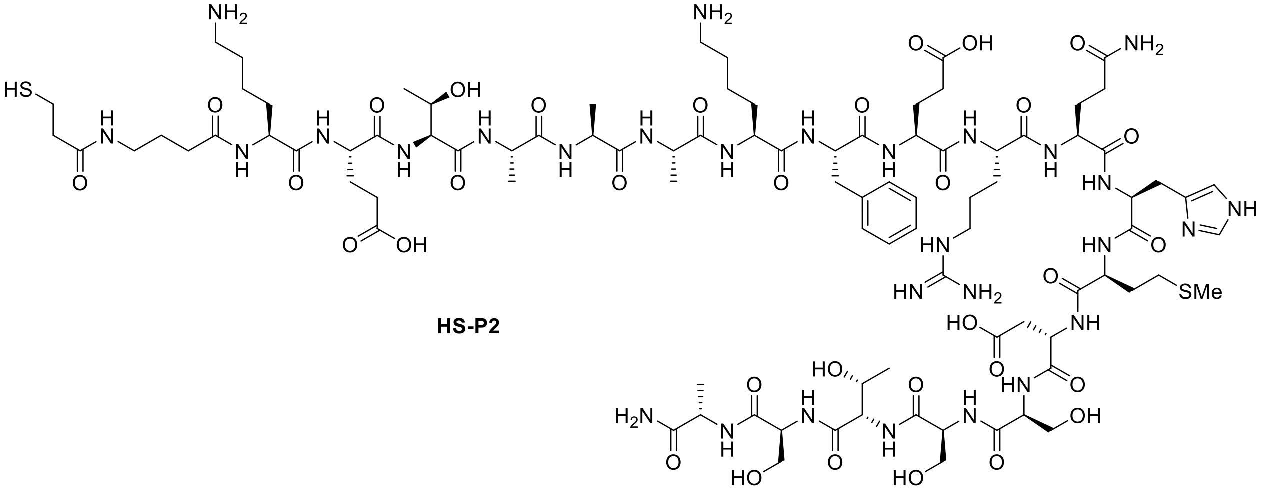

HS-P2

LRMS (ESI$^+$): *m/z* Calcd for $C_{93}H_{153}N_{29}O_{33}S_2^{2+}$: 1134.52 $[M]^{2+}$;Observed: 1134.52.

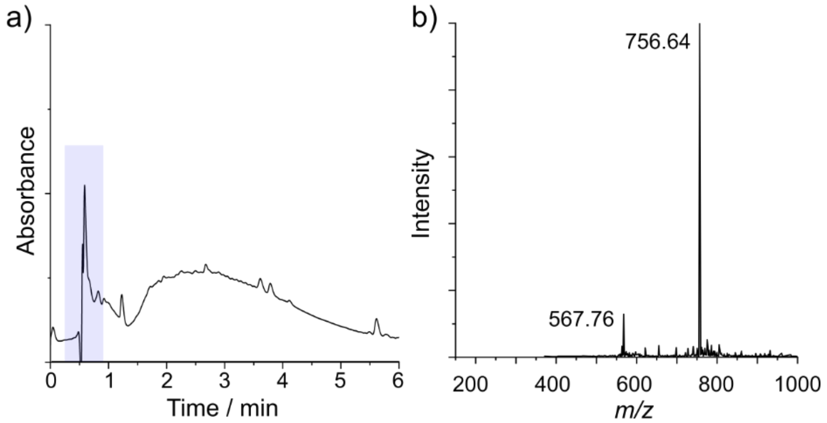

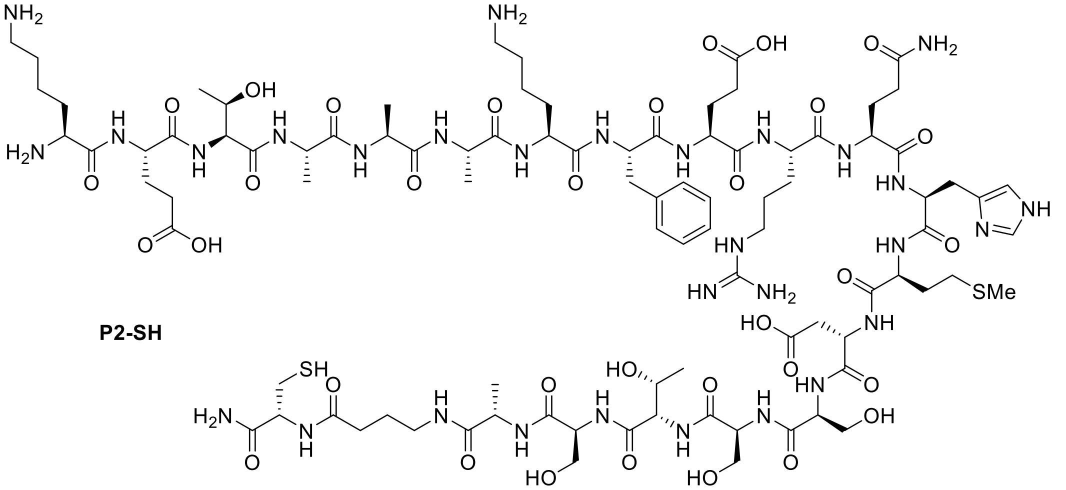

P2-SH

HRMS (ESI$^+$): *m/z*

Calcd for $C_{93}H_{154}N_{30}O_{33}S_2$: 1141.5363 $[M+2H]^{2+}$; Observed: 1141.5353.

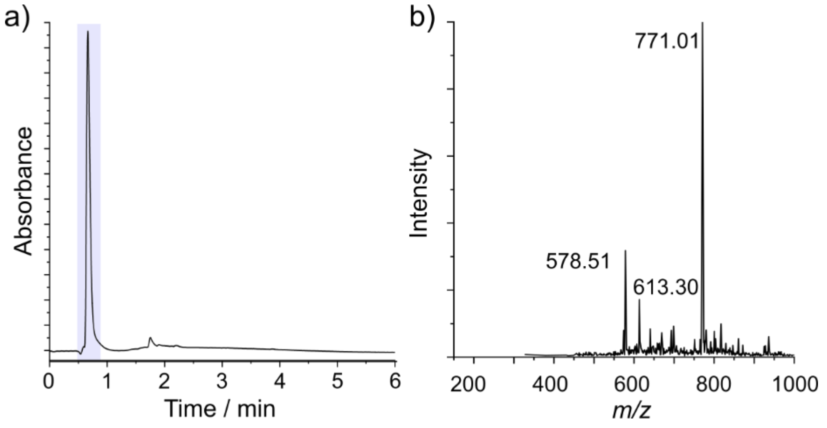

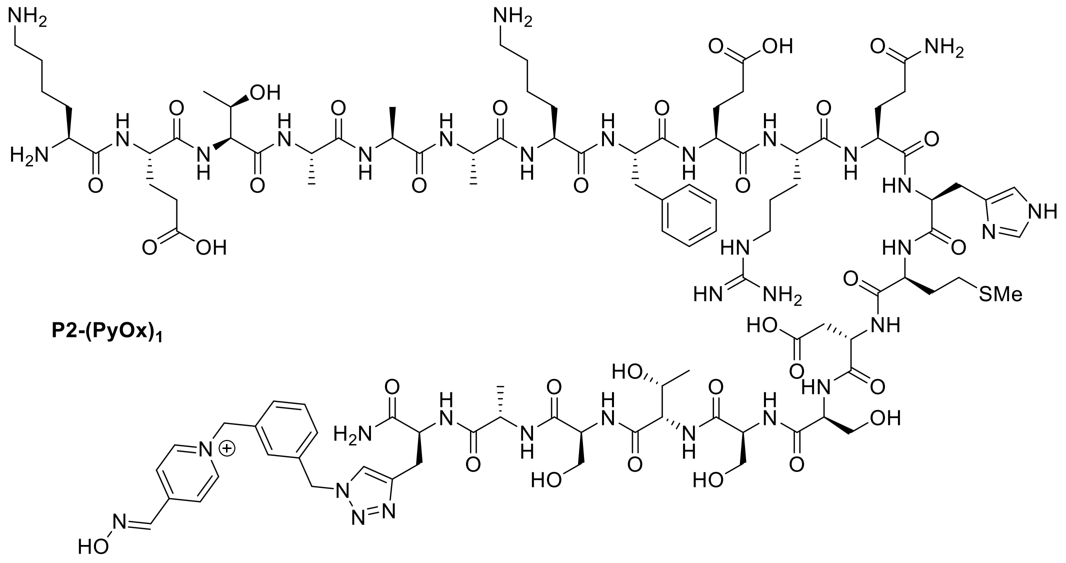

P2-(PyOx)$_1$

HRMS (ESI$^+$): *m/z*

Calcd for $C_{105}H_{160}N_{34}O_{33}S^+$: 1228.5742 $[M+H]^{2+}$; Observed: 1228.5798.

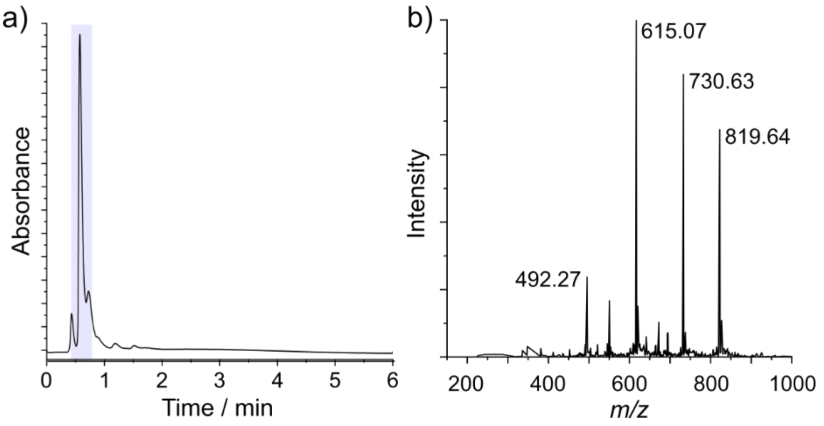

4. QCM-D

Synthesis of blocking agent

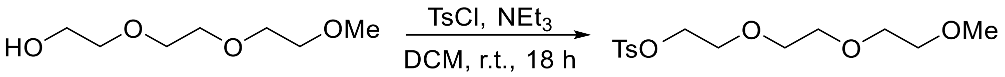

15

A solution of *para*-toluenesulfonic acid (2.5 g, 13.1 mmol) in DCM (5 mL) was added dropwise to a solution of triethylamine (2.5 mL, 17.9 mmol) and triethylene glycol monomethyl ether (2.0 g, 12.2 mmol) in DCM (30 mL) at 0 °C over 5 min. The mixture was allowed to warm to r.t. and stirred for 18 h. The reaction was then quenched through the addition of hydrochloric acid (4 M, 20 mL) and extracted with DCM (3 × 40 mL). The combined organics were dried with $MgSO_4$, filtered, and concentrated *in vacuo*. The residue was then purified by flash column chromatography, eluting with 33% EtOAc:Petrol. Pure fractions were concentrated *in vacuo* to provide product **15** as a pale-yellow liquid (2.5 g, 7.9 mmol, 64%). Data were consistent with those previously reported.[8]

^{1}H NMR (400 MHz, $CDCl_3$) δ 7.66 (2H, d, J = 8.0 Hz, Ts), 7.22 (2H, d, J = 8.0 Hz, Ts), 4.01-4.06 (2H, m, -CH_2OTs), 3.51-3.59 (2H, m, PEG), 3.44-3.49 (6H, m, PEG), 3.37-3.41 (2H, m, PEG), 3.23 (3H, s, -OMe), 2.23 (3H, s, Ts).

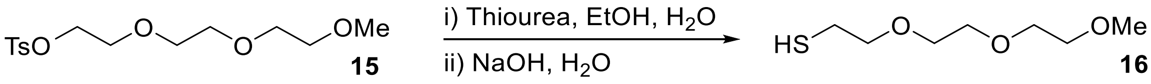

A mixture of **15** (1.5 g, 4.72 mmol) and thiourea (360 mg, 4.74 mmol) in ethanol (3 mL) and water (2 mL) was refluxed for 3 h. After cooling to r.t., sodium hydroxide (230 mg, 5.75 mmol) and water (2 mL) were added and the mixture refluxed for a further 1.5 h. After cooling to r.t. the mixture was acidified with conc. hydrochloric acid and extracted with DCM (3 × 10 mL). The combined organics were dried with $MgSO_4$, filtered, and concentrated *in vacuo* to afford product **16** as a colourless liquid (0.60 g, 3.33 mmol, 71%). Data were consistent with those previously reported.[8]

^{1}H NMR (400 MHz, $CDCl_3$) δ 3.51-3.67 (10H, m, PEG), 3.37 (3H, s, -OMe), 2.68 (2H, dt, J = 8.1, 6.3 Hz, -CH_2SH), 1.57 (1H, t, J = 8.1 Hz, -SH).

Sensor preparation: QCM-D gold sensors (QSX 301, Biolin Scientific) were cleaned by UV–ozone treatment (30 min), followed by sonication in a 2% Hellmanex III solution (5 min) and then sonication in ultrapure water (5 min). Sensors were then dried with N_2 gas.

Binding studies: Following cleaning, each sensor was installed into the flow modules of a Q-Sense E4, QFM 401 system by Biolin Scientific. Each chamber was then filled with Milli-Q water at a flow rate of 100 μL/min controlled by a four-channel peristaltic pump. To achieve a stable baseline, PBS was left to flow through the modules at 100 μL/min until the drift in frequency was <±1 Hz over 10 min. For all experiments, the temperature of the modules was kept at 20 °C (standard deviation 5×10^{-3} °C) and the flow rate was kept constant at 100 μL/min. Sensors were functionalized in flow starting with a solution of cysteine-labelled peptide in PBS (t_1, 10 μM, 1 mL) followed by a blocking solution of **16** (t_2, 10 μM, 1 mL). Buffer was allowed to flow over the sensor surface between sample injections to ensure excess reagents were removed (t_3). Once the peptide surface was prepared and stable (t_4), 1 mL of each concentration of protein was then inserted at the same flow rate. The range of concentration for insulin and RNase-A was 0.05, 0.1, 0.5, 1, 5, 10, 50, 100 μM.

Data processing: 4 data points for each concentration were averaged to calculate the delta frequency versus a control using PBS. The data were then fit to a sigmoidal function.

Insulin

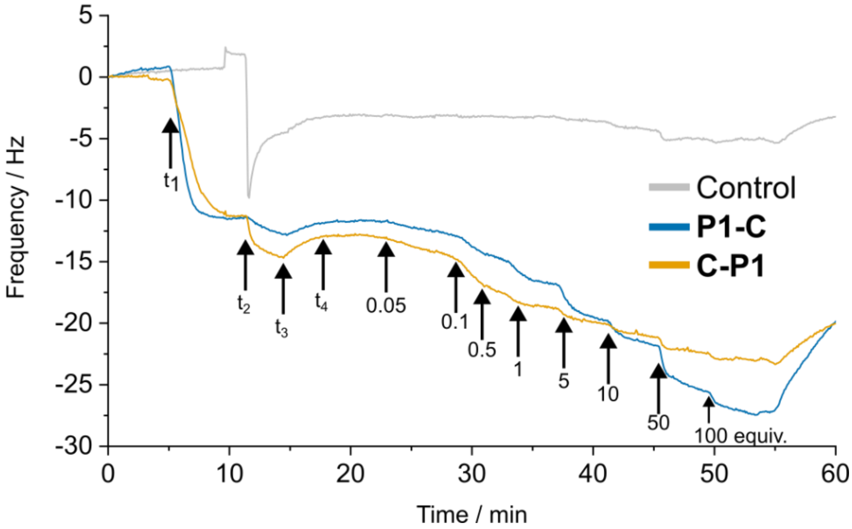

Figure S1: QCM-D curves obtained for the binding of **C-P1** and **P1-C** and insulin on a gold surface, as well as a control in the absence of peptide. t_1 = addition of peptide; t_2 = addition of compound **16** to minimise non-specific binding; t_3 = washing step with PBS. All subsequent timepoints represent the addition of increasing equivalents of insulin relative to peptide.

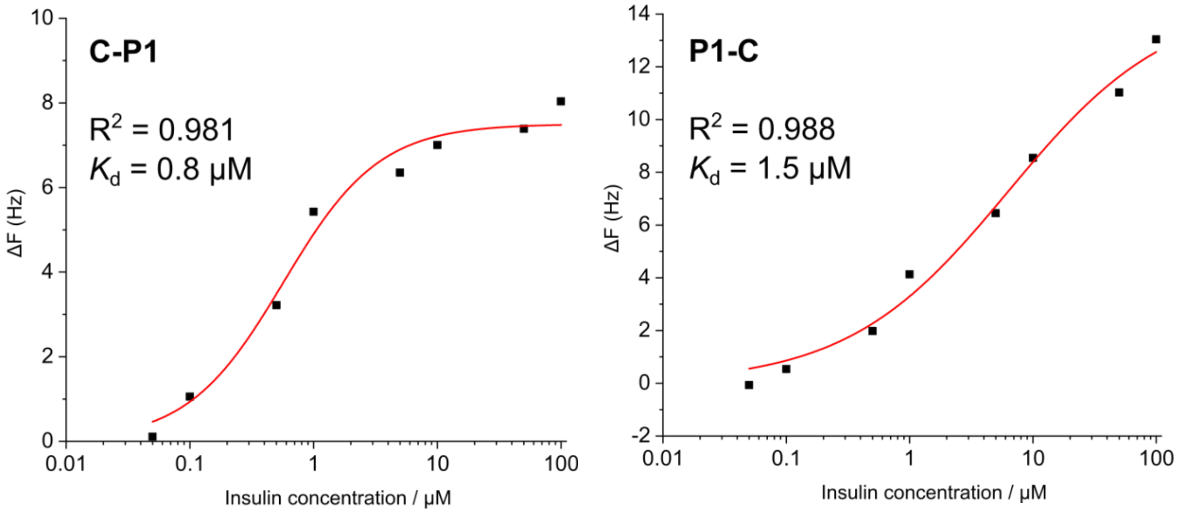

Figure S2: Binding curves for **C-P1** and **P1-C** to insulin extracted from the QCM-D binding data.

RNase A

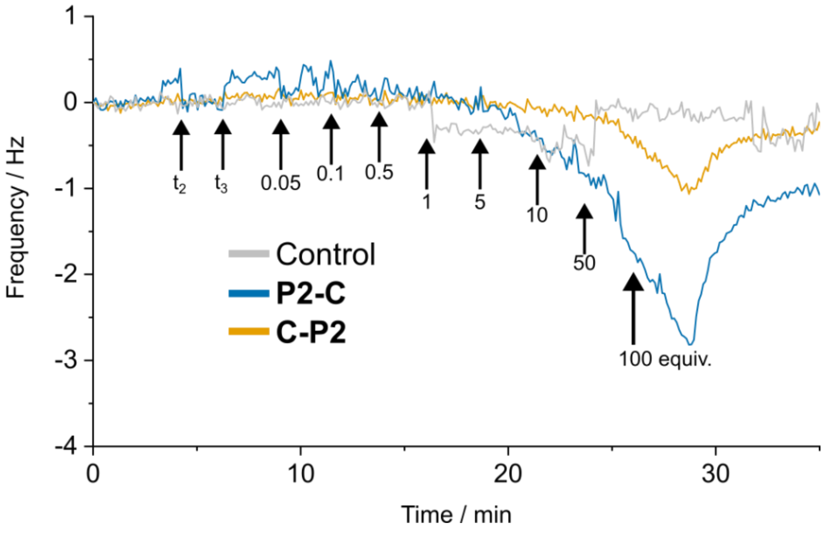

Figure S3: QCM-D curves obtained for the binding of **HS-P2** and **P2-SH** and RNase A on a gold surface, as well as a control in the absence of peptide. t_2 = addition of compound **16** to minimise non-specific binding; t_3 = washing step with PBS. All subsequent timepoints represent the addition of increasing equivalents of RNase A relative to peptide.

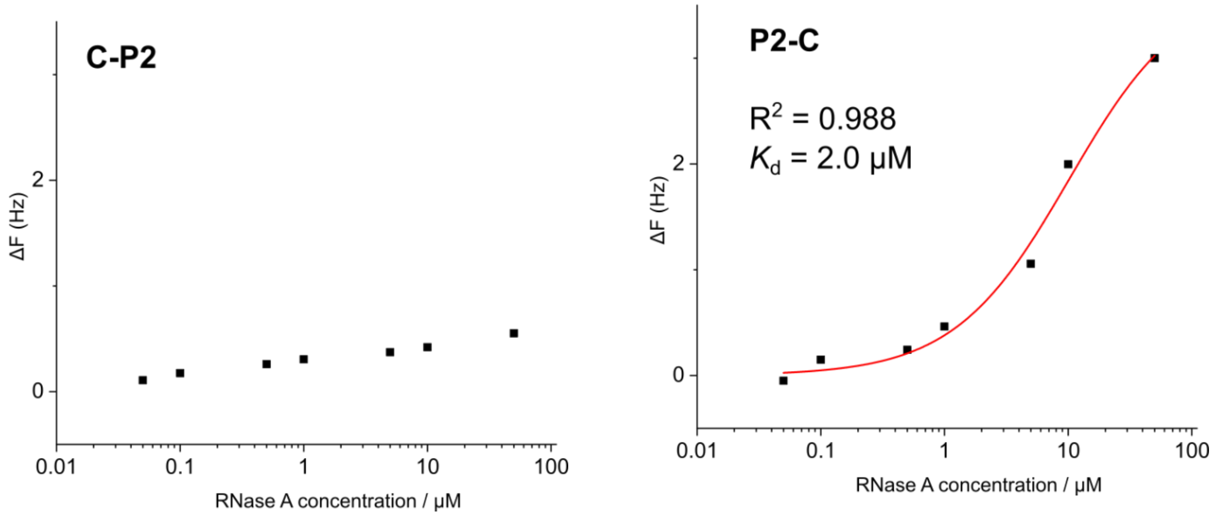

Figure S4: Binding curves for **HS-P2** and **P2-SH** to insulin extracted from the QCM-D binding data.

4. Protein labelling

General protocol: A solution of PyOx-peptide probe (250 µM in pH 7.4 PBS, 25 µL, 6.25 nmol; Final concentration 122 µM) was added to a solution of insulin (50 µM in pH 7.4 PBS, 25 µL, 1.25 nmol; Final concentration 25 µM) and shaken for 30 min. A solution of biotin-NASA **3b** (25 mM in DMSO, 1 µL, 25 nmol; Final concentration 490 µM) was then added and the mixture shaken at room temperature for 2 h. Samples were then analysed by LC-MS without purification.

P1-(PyOx)1

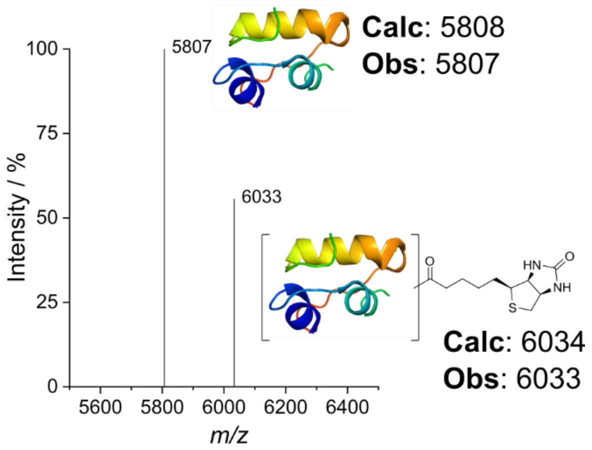

Figure S5: Deconvoluted mass spectra from general labelling protocol showing modified and unmodified insulin.

Varying peptide concentration: Run as described above, varying the amount of **P1-(PyOx)1** added (0, 1.25, 2.5, or 10 equiv). For 0-2.5 equiv, a 500 µM stock solution was used and volumes were made up to 25 µL before addition to the protein. For 10 equiv, a 1 mM stock solution was used.

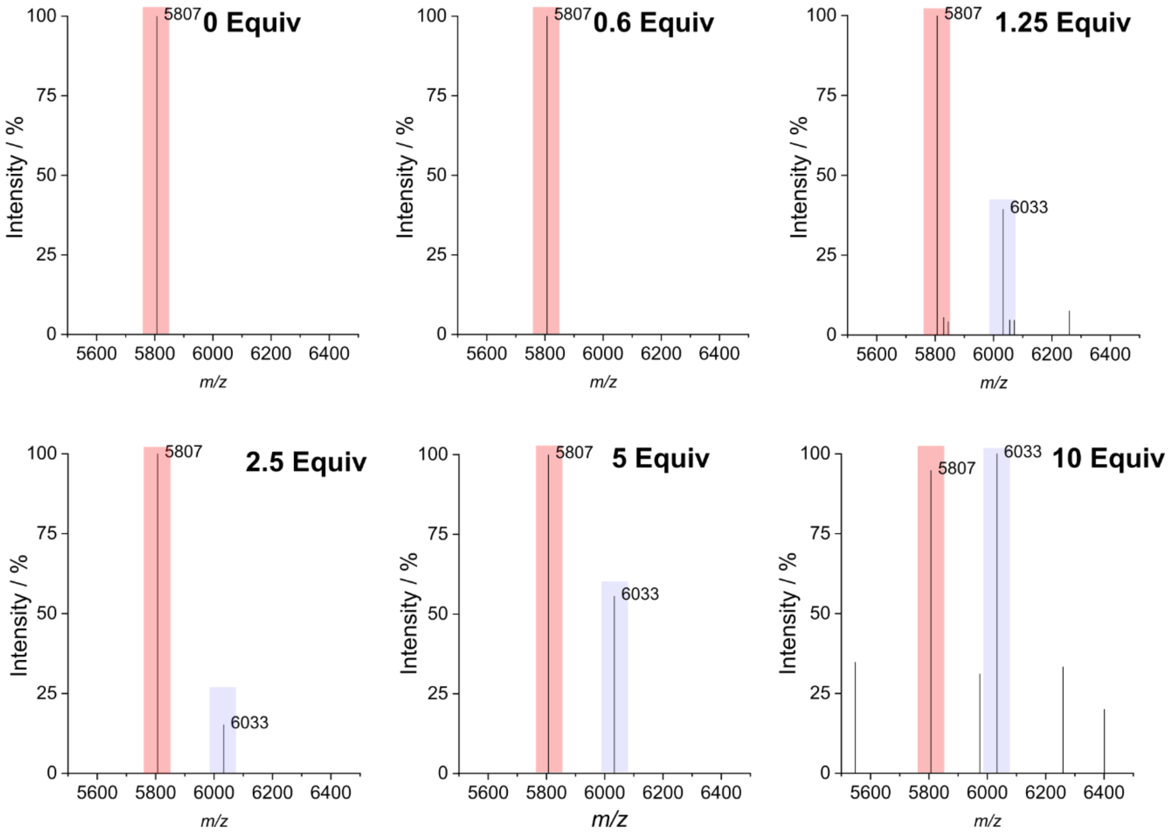

Figure S6: Deconvoluted mass spectra with different equivalents of peptide catalyst.

Varying pH: Run as described above, but with the peptide and PyOx-peptide stocks dissolved in PBS buffer adjusted to either pH 6 or pH 8. In the case of reactions run at pH 6, no peaks relating to insulin were observed by LC-MS. Nb. Additional peaks observed at with 10 equiv. are the result of signal background during deconvolution, and not off-target protein labelling.

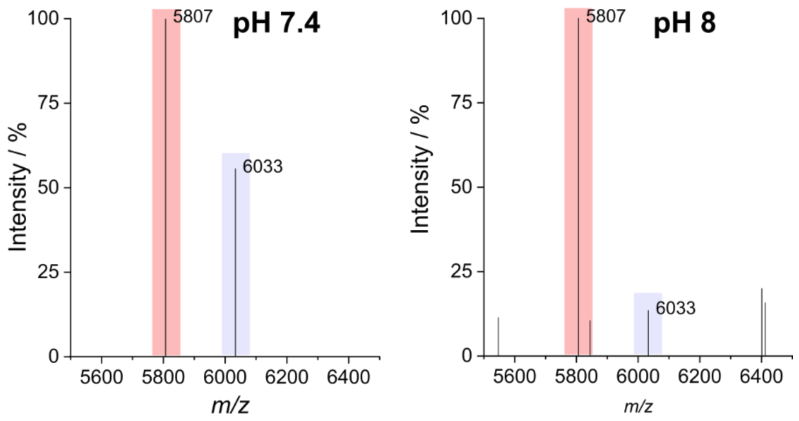

Figure S7: Deconvoluted mass spectra following labelling at different pHs.

Varying reaction time: Run as described above over 24 h.

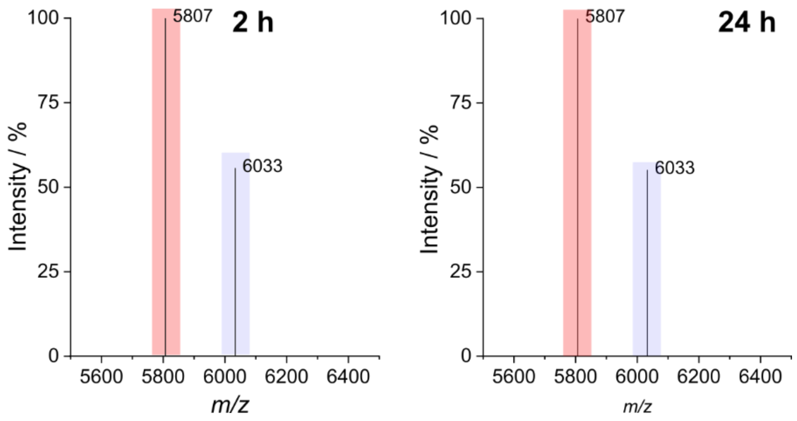

Figure S8: Deconvoluted mass spectra following labelling at different time.

Varying temperature: Run as described above at either 4 °C or 37 °C.

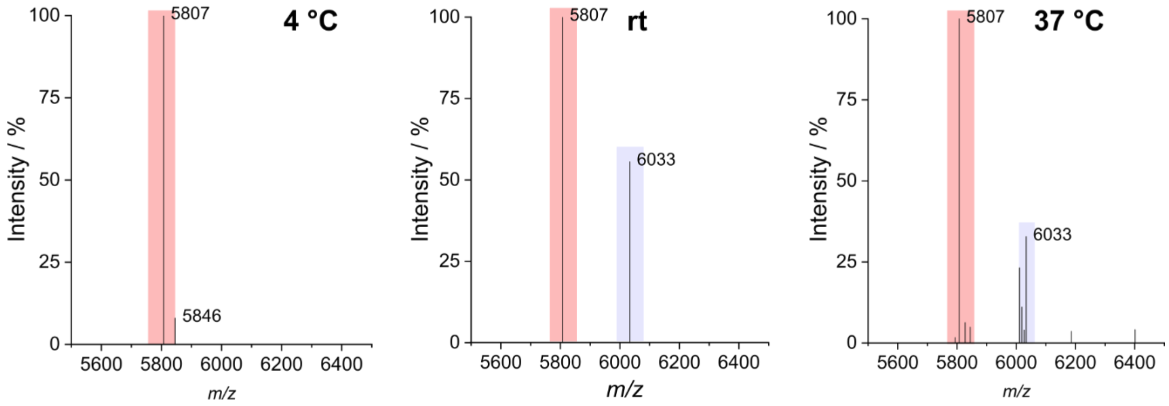

Figure S9: Deconvoluted mass spectra following labelling at different temperatures.

Temperature controls: Run as described above, in the absence of peptide, at either 4 °C or 37 °C.

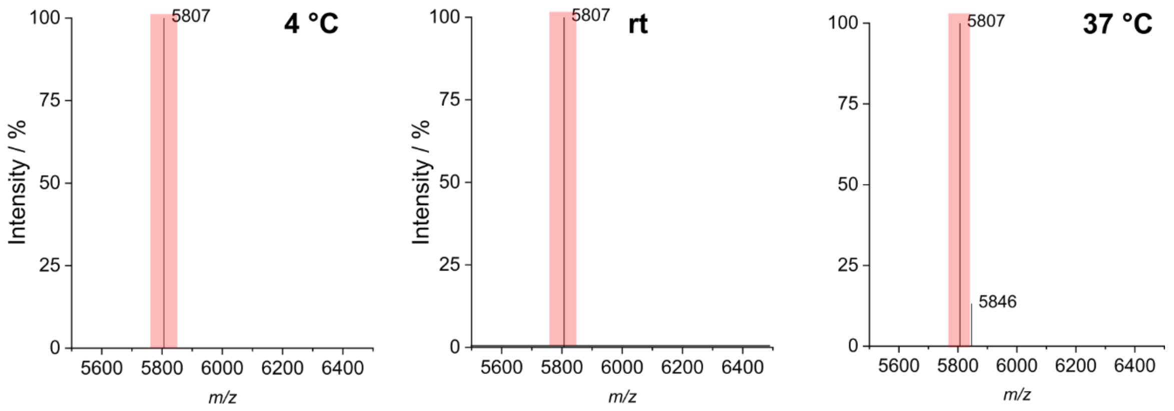

Figure S10: Deconvoluted mass spectra following labelling at different temperatures in the absence of peptide catalysts.

Varying NASA probe: Run as described above with NBD-NASA (**3c**).

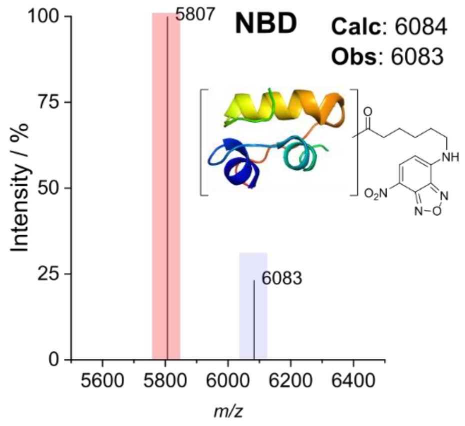

Figure S11: Deconvoluted mass spectra following labelling with NASA **3c**.

Varying peptide: Run as described above with either **(PyOx)$_1$-P1** or **P1-(PyOx)$_2$**.

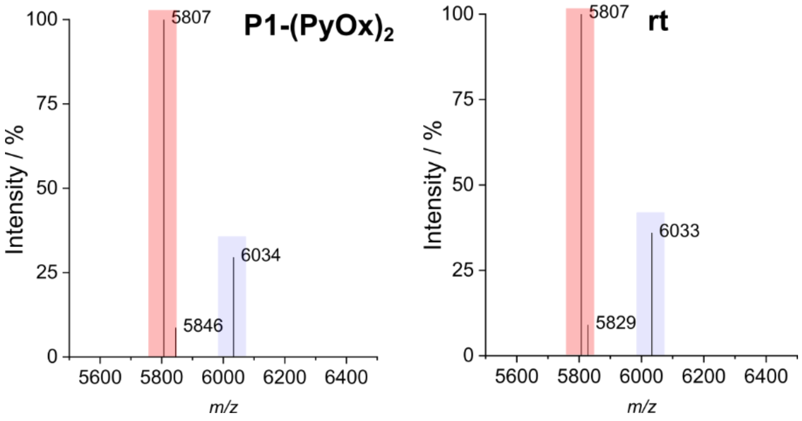

Figure S12: Deconvoluted mass spectra following labelling with different peptide catalysts.

5. (T)SDS-PAGE analysis

Insulin: Crude reaction mixtures, prepared as described in Section 4 with NBD-NASA **3c**, were analysed directly by SDS-PAGE without purification. Protein samples (5 µL) were diluted with dye-free SDS sample buffer (2% SDS, 4% glycerol, 40 mM Tris-HCl, pH 6.8; 2.5 µL) and water (5 µL). Samples were loaded directly onto pre-cast SDS-PAGE gels (BIO-RAD Mini-PROTEAN TGX Gel, 4-15% acrylamide). Samples were separated at 200 V for 20 min, and then either subjected to fluorescent imaging or stained using InstantBlue protein stain for 30 min and imaged using a Syngene G:BOX Chemi XRQ imager equipped with a Synoptics 4.0 Mp camera.

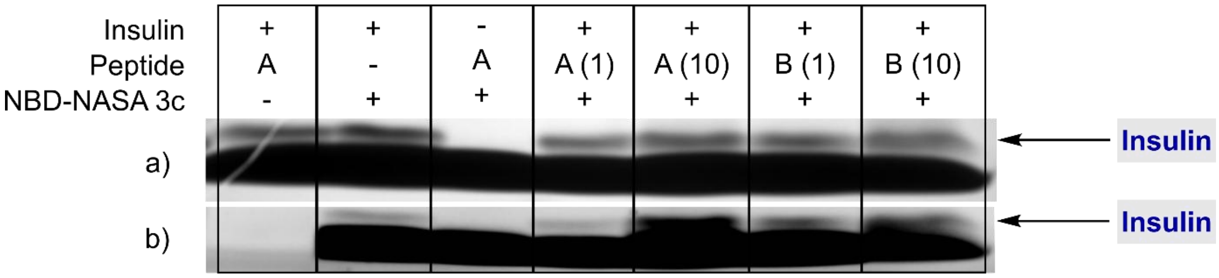

Figure S13: SDS-PAGE analysis of insulin labelled with NBD-NASA **3c** and peptides **(PyOx)$_1$-P1** (denoted peptide A, equiv. w.r.t. protein in parentheses) or **P1-(PyOx)$_1$** (denoted peptide B); a) stained with InstaBlue protein stain; b) fluorescence imaging. Nb. Due to its low molecular weight insulin runs very close to the running front, and so this has been included in for clarity in the images. In a) this can be seen as the running

buffer front stained with InstantBue in all lanes; in b) it is seen as a fluorescent band from unreacted NBD-NASA **3c** where included in the reaction mixture. The full gel can be found in Section 8.

RNase A: Crude reaction mixtures, prepared as described in Section 4 with Cy5-NASA **3d**, were purified by dialysis (3.5 kDa micro-dialysis cassette, ThermoFisher, 18 h). Protein samples (5 µL) were diluted with dye-free SDS sample buffer (3 µL) and water (5 µL). Samples were heated to 95 °C for 5 min, and then after cooling and centrifugation, loaded directly onto a 6% acrylamide stacking/15% acrylamide resolving tricine gel. Samples were separated, initially at 30 mA current for 30-45 min until all samples had accumulated at the stacking/resolving gel interface. The current was then increased to 75 mA for 2-3 hr. Washed gels were then either subjected to fluorescent imaging or stained using Coomassie Blue protein stain for 18 h, and imaged using an Amersham Typhoon 5 Bioimager.

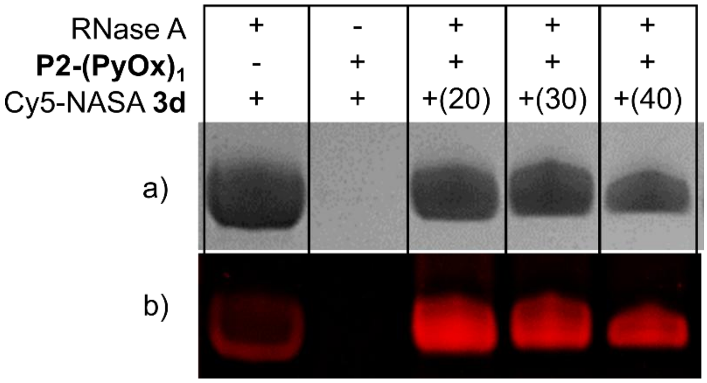

Figure S14: TSDS-PAGE analysis of RNase A labelled with Cy5-NASA **3d** (equivalents w.r.t. protein in parantheses) and peptide **P2-(PyOx)$_1$**; a) stained with Coomassie Blue; b) fluorescence imaging. Nb. The full gel can be found in Section 8.

6. Western blot analysis

Gels of reaction mixtures, prepared as described in Section 4 with Biotin-NASA **3a**, were prepared as described in Section 5. Gels were transferred onto Amersham Hybond P PVDF Membranes using a Power Blotter XL System (Invitrogen) following the manufacturers instructions. Following transfer the membrane was incubated in blocking buffer (0.1% Tween-20, 5% non-fat dry milk powder in PBS; 5 mL) for 1 h. The membrane was washed (0.1% Tween-20 in PBS; 5 mL, 3 × 5 min), and then

inclubated with anti-biotin horseradish peroxidase GTX77581 antibody (1/1000 dilution in PBS, 0.1% Tween-20) for 1 h. After washing the membrane (0.1% Tween-20 in PBS; 5 mL, 3 × 5 min) it was incubated with a BCIP/NBT Alkaline Phosphatase Substrate Kit (Vector Labs) until proteins were visible on the membrane (~ 20 min). The membrane was then washed with water and imaged using a Syngene G:BOX Chemi XRQ imager equipped with a Synoptics 4.0 Mp camera.

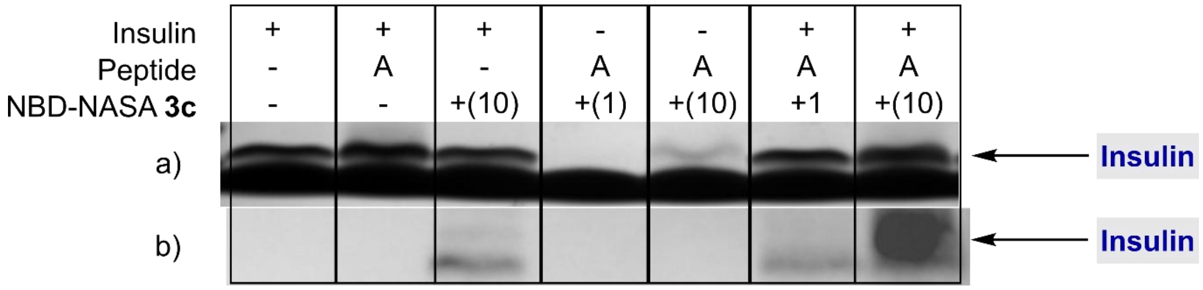

Figure S15: Western blot analysis of insulin labelled with Biotin-NASA **3c** (parentheses denote equivalents w.r.t. peptide) and peptide **$(PyOx)_1$-P1** (A); a) SDS-PAGE stained with InstaBlue protein stain; b) anti-biotin western blot. Nb. Due to its low molecular weight insulin runs very close to the running front in the SDS-PAGE gel, stained with InstantBue in all lanes. The full gel can be found in Section 8.

7. MALDI protein fragmentation analysis

For insulin, Protein or peptide samples were diluted to a concentration of 10 µM with water, before the addition of a solution of TCEP (50 mM, 1 µL) and incubation at 60 °C for 1 h. Post-reduction, proteins were alkylated with MMTS (200 mM, 0.5 µL) or iodoacetamide (100 mM, 1 µL) for 10 min.

A 0.5 µL aliquot of the protein mixture was applied to a ground steel MALDI target plate, followed immediately by an equal volume of a freshly-prepared solution of 4-hydroxy-α-cyano-cinnamic acid (10 mg/mL in 50% aqueous (v:v) acetonitrile containing 0.1% trifluoroacetic acid).

Positive-ion MALDI mass spectra were obtained using a Bruker ultrafleXtreme mass spectrometer in reflectron mode, equipped with a Nd:YAG smart beam laser. MS spectra were acquired over a range of *m/z* 900-7000. Final mass spectra were externally calibrated against an adjacent spot containing 6 peptides (des-Arg^1-

Bradykinin, 904.681; Angiotensin I, 1296.685; Glu[1]-Fibrinopeptide B, 1750.677; ACTH (1-17 clip), 2093.086; ACTH (18-39 clip), 2465.198; ACTH (7-38 clip), 3657.929.). Monoisotopic masses were obtained using the SNAP averaging algorithm (C 4.9384, N 1.3577, O 1.4773, S 0.0417, H 7.7583) and an S/N threshold of 2.

Precursors were manually selected for LIFT MS/MS fragmentation without the introduction of a collision gas. The laser power and number of spectra summed was manually optimised for each precursor. The default calibration was used for MS/MS spectra, which were baseline-subtracted and smoothed (Savitsky-Golay, width 0.15 m/z, cycles 4); monoisotopic peak detection used a SNAP averaging algorithm (C 4.9384, N 1.3577, O 1.4773, S 0.0417, H 7.7583) with a minimum S/N of 6. Bruker flexAnalysis software (version 3.3) was used to perform spectral processing and peak list generation, and manual annotation of peptide modifications.

As the reduced insulin A-chain was not observed by MALDI acquisition, analysis was repeated using nanoLC-MS/MS. Peptides were loaded onto EvoTip Pure tips for desalting and as a disposable trap column for nanoUPLC using an EvoSep One system. A pre-set EvoSep 100 SPD gradient was used with a 8 cm EvoSep C_{18} Performance column (8 cm x 150 μm x 1.5 μm). The nanoUPLC system was interfaced to a timsTOF HT mass spectrometer (Bruker) with a CaptiveSpray ionisation source (Source). Positive PASEF-DDA, ESI-MS and MS^2 spectra were acquired using Compass HyStar software (version 6.2, Thermo). Instrument source settings were: capillary voltage, 1,500 V; dry gas, 3 l/min; dry temperature; 180°C. Spectra were acquired between *m/z* 100-1,700. The following TIMS settings were applied as: 1/K0 0.6-1.60 V.s/cm^2; Ramp time, 100 ms; Ramp rate 9.42 Hz. Data dependant acquisition was performed with 10 PASEF ramps and a total cycle time of 1.17 s. An intensity threshold of 2,500 and a target intensity of 20,000 were set with active exclusion applied for 0.4 min post precursor selection. Collision energy was interpolated between 20 eV at 0.5 V.s/cm^2 to 59 eV at 1.6 V.s/cm^2. Spectra were searched using PEAKS Studio 11 (Bioinformatic Solutions Inc.) against the theoretical sequence of insulin, specifying no enzymatic cleavage and allowing for variable modification by biotinylation. Peptide matches were filtered to 1% FDR and verified by manual inspection.

RNase A samples were reduced and alkylated as described for insulin, before incubation with 0.2 μg sequencing grade trypsin (Promega) overnight at 37°C. Resulting peptides were measured by MALDI-MS/MS with LIFT fragmentation as detailed for insulin.

Insulin labelling with biotin-NASA 3b and $(PyOx)_1$-P1

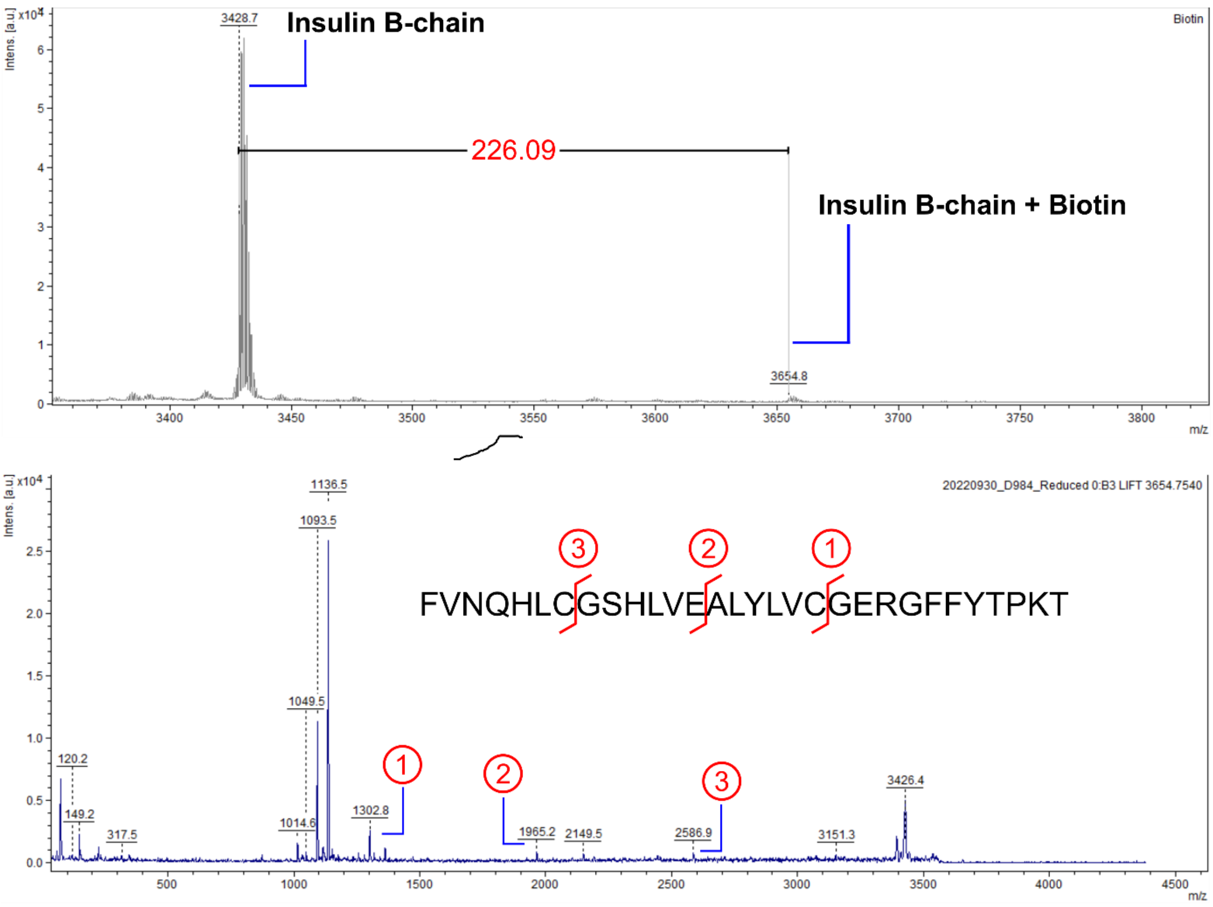

Figure S16: MALDI spectra of biotin-labelled insulin B-chain before and after LIFT-MS/MS fragmentation.

Insulin labelling with biotin-NASA 3b and P1-(PyOx)1

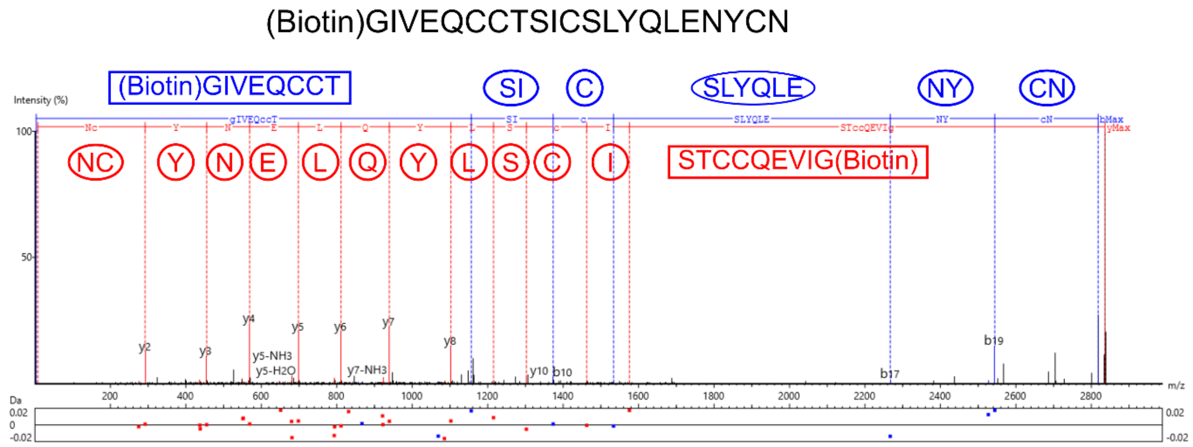

Figure S17: NanoLC-MS/MS spectra of biotin-labelled insulin A-chain

RNase labelling with biotin-NASA 3b

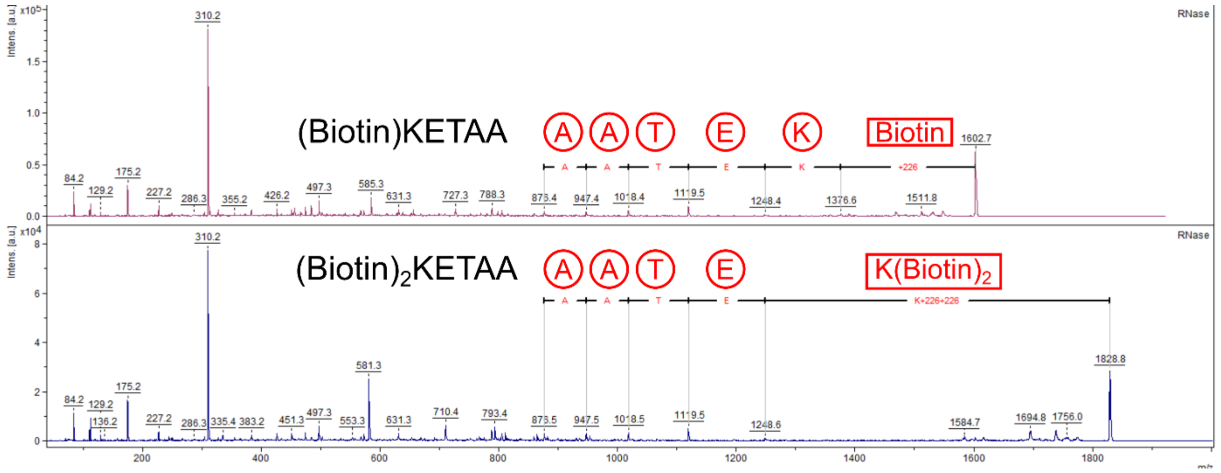

Figure S18: MALDI spectra of trypsin-digested, biotin-labelled RNase A after LIFT-MS/MS fragmentation, showing both mono- and di-biotinylation at the N-terminal lysine.

8. Peptide self-labelling

Reactions were run as described in the general procedure of Section 4, in the absence of added protein, using peptides **P1-(PyOx)₂** or **P1-(PyOx)₁** (i.e. just peptide plus biotin-NASA **3a**). At *t* = 2h and *t* = 24 h samples were analysed by LC-MS to identify peaks relating to the peptide or its modification products. Samples were then analysed by MALDI-MS/MS analysis to identify fragmentation patterns and potential sites of labelling.

Hexanoic-NASA 3a

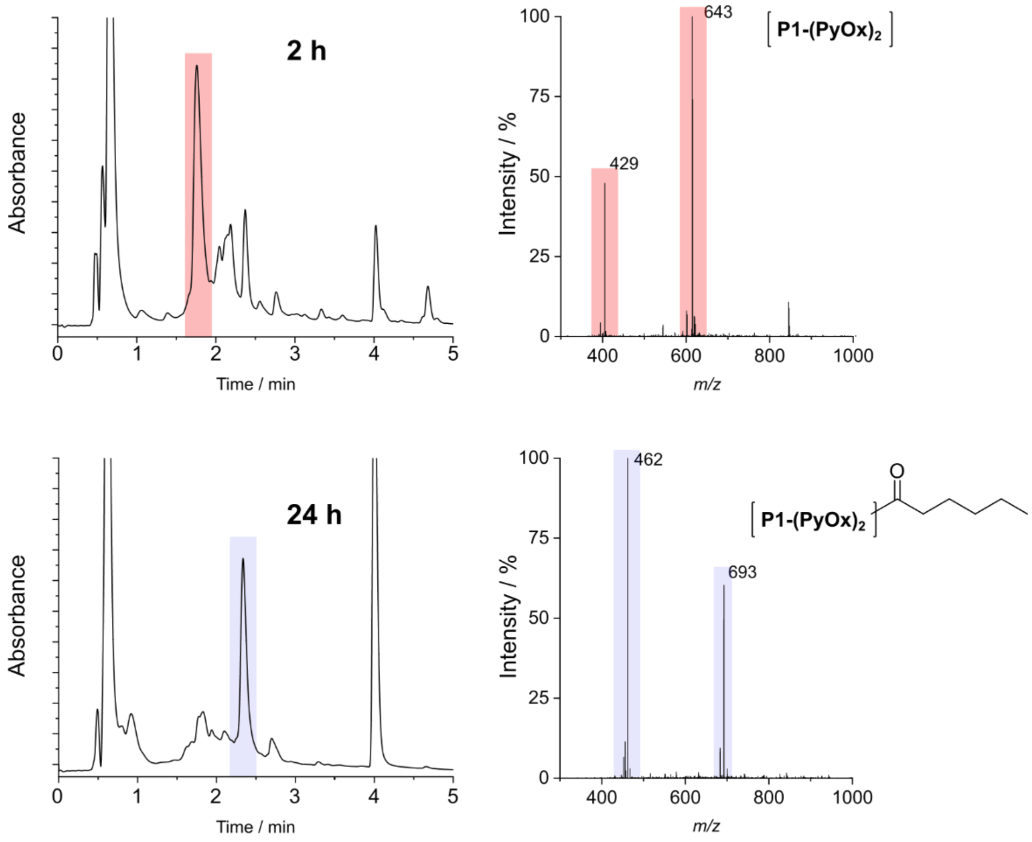

Biotin-NASA 3b

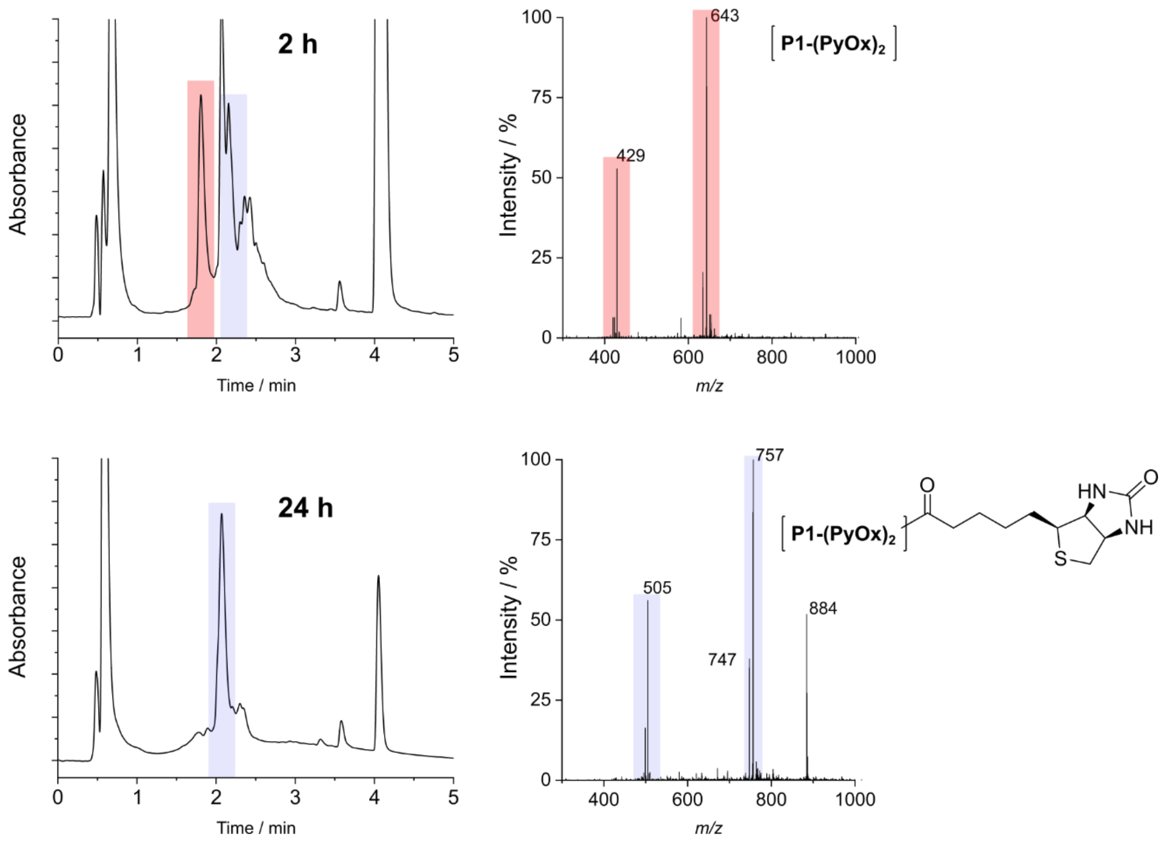

Figure S19. LC-MS traces and deconvoluted mass spectra for the labelling of peptide catalysts with NASA reagents.

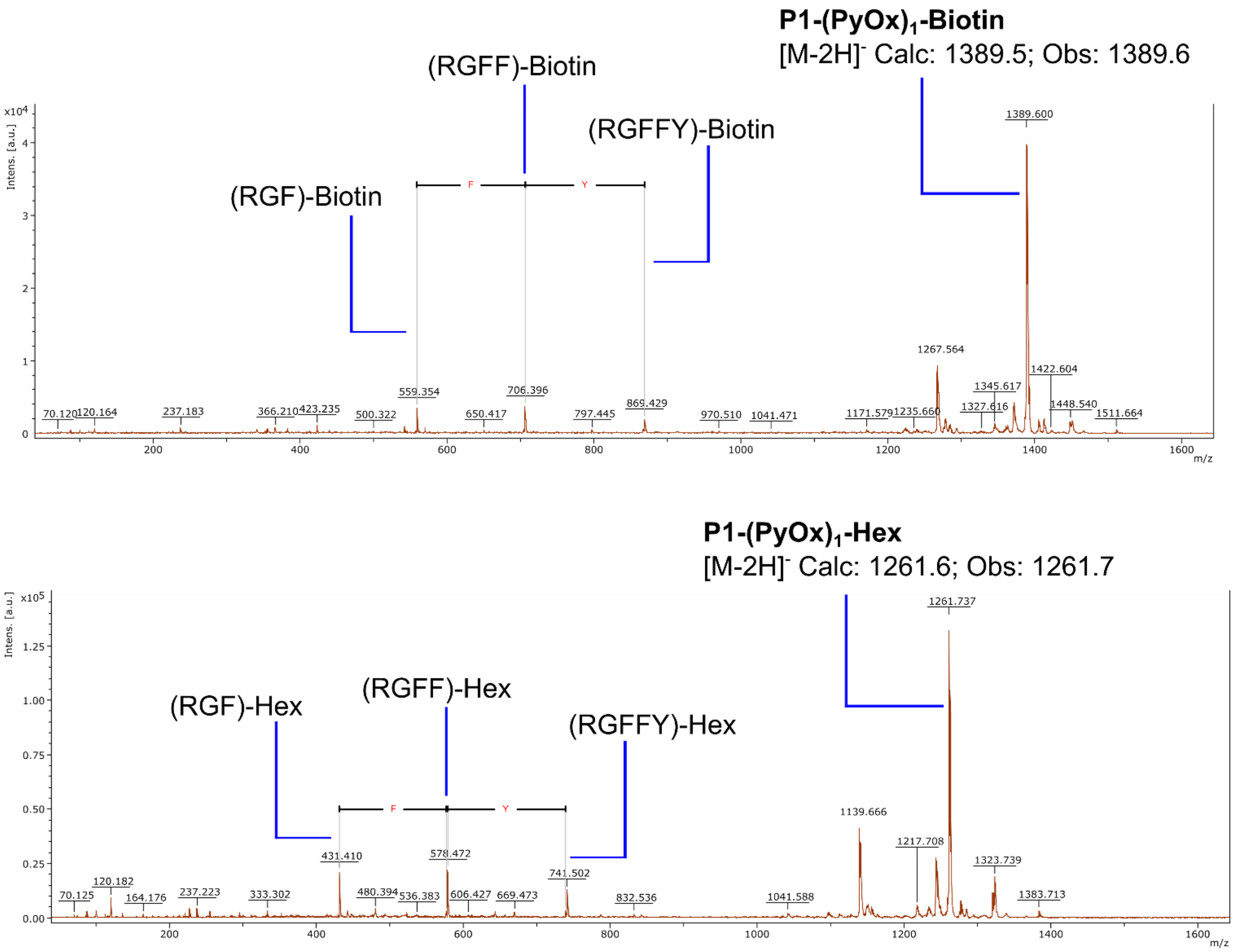

Figure S20: MALDI-MS/MS spectra of biotin- and hexanoic acid-labelled **P1-(PyOx)$_1$**.

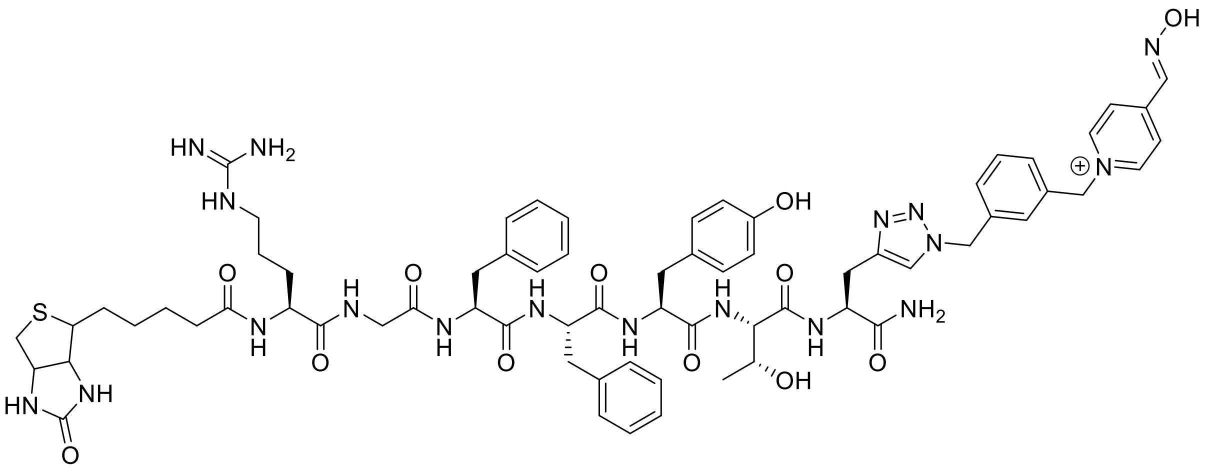

Proposed **P1-(PyOx)$_1$-Biotin** structure

8. Full gel images

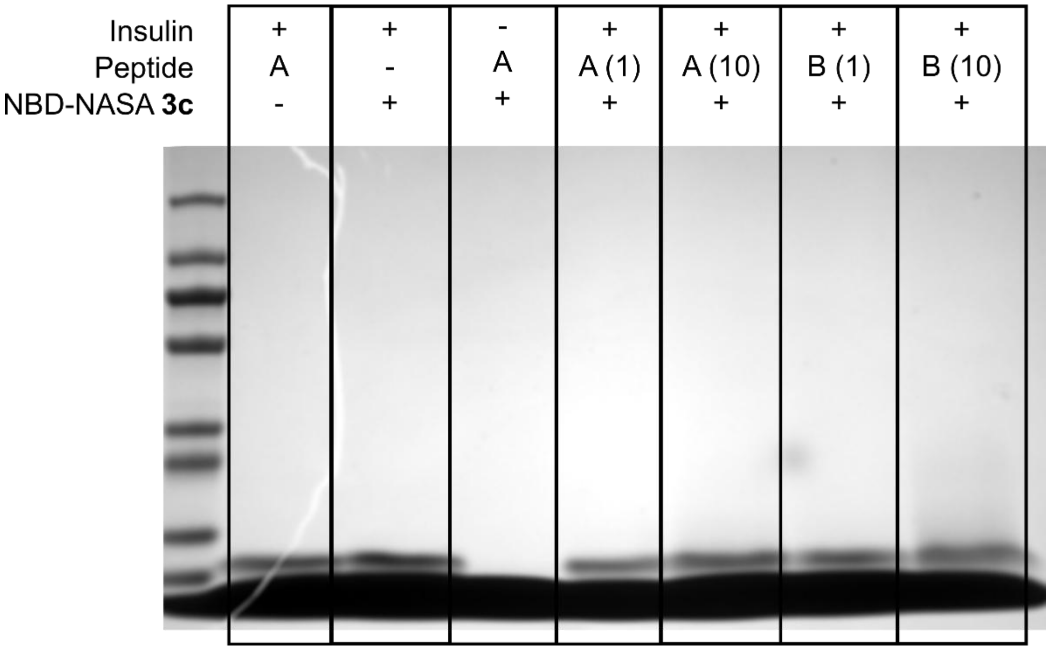

Figure S21: Full version of Fig. S13, showing SDS-PAGE analysis of insulin labelled with NBD-NASA **3c** and peptides **$(PyOx)_1$-P1** (denoted peptide A, equiv. w.r.t. protein in parentheses) or **P1-$(PyOx)_1$** (denoted peptide B).

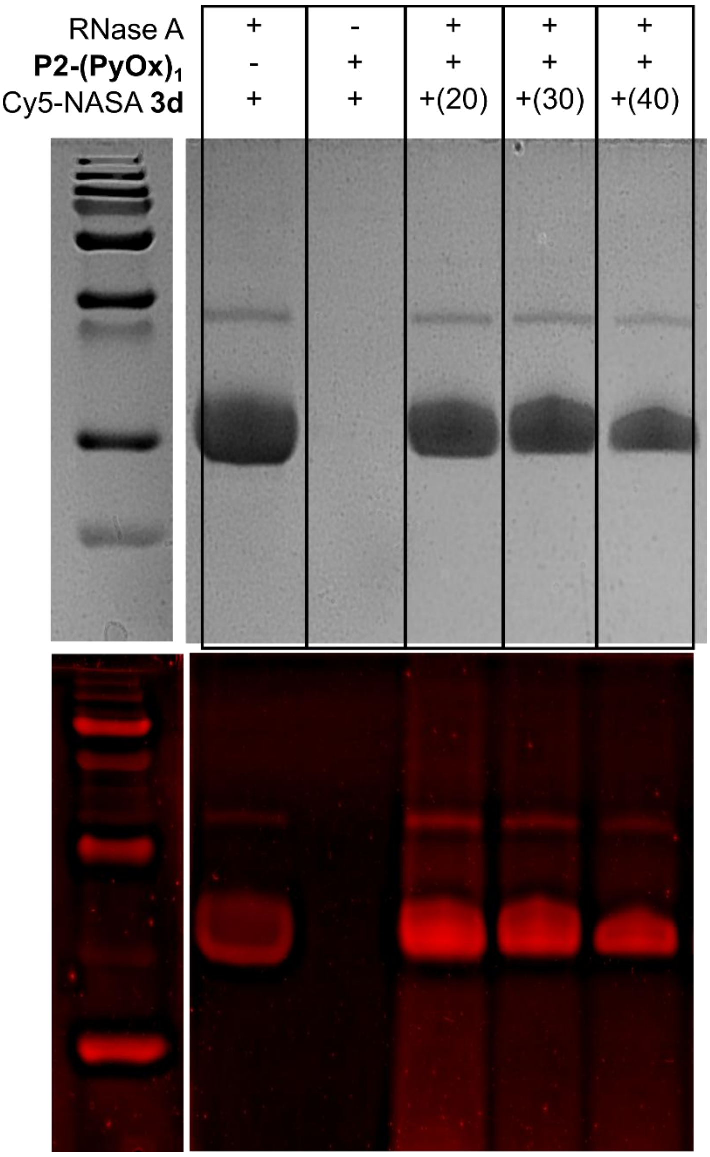

Figure S22: Full version of Fig. S14, showing TSDS-PAGE analysis of RNase A labelled with Cy5-NASA **3d** (equivalents w.r.t. protein in parantheses) and peptide **P2-(PyOx)$_1$**; a) stained with Coomassie Blue; b) fluorescence imaging.

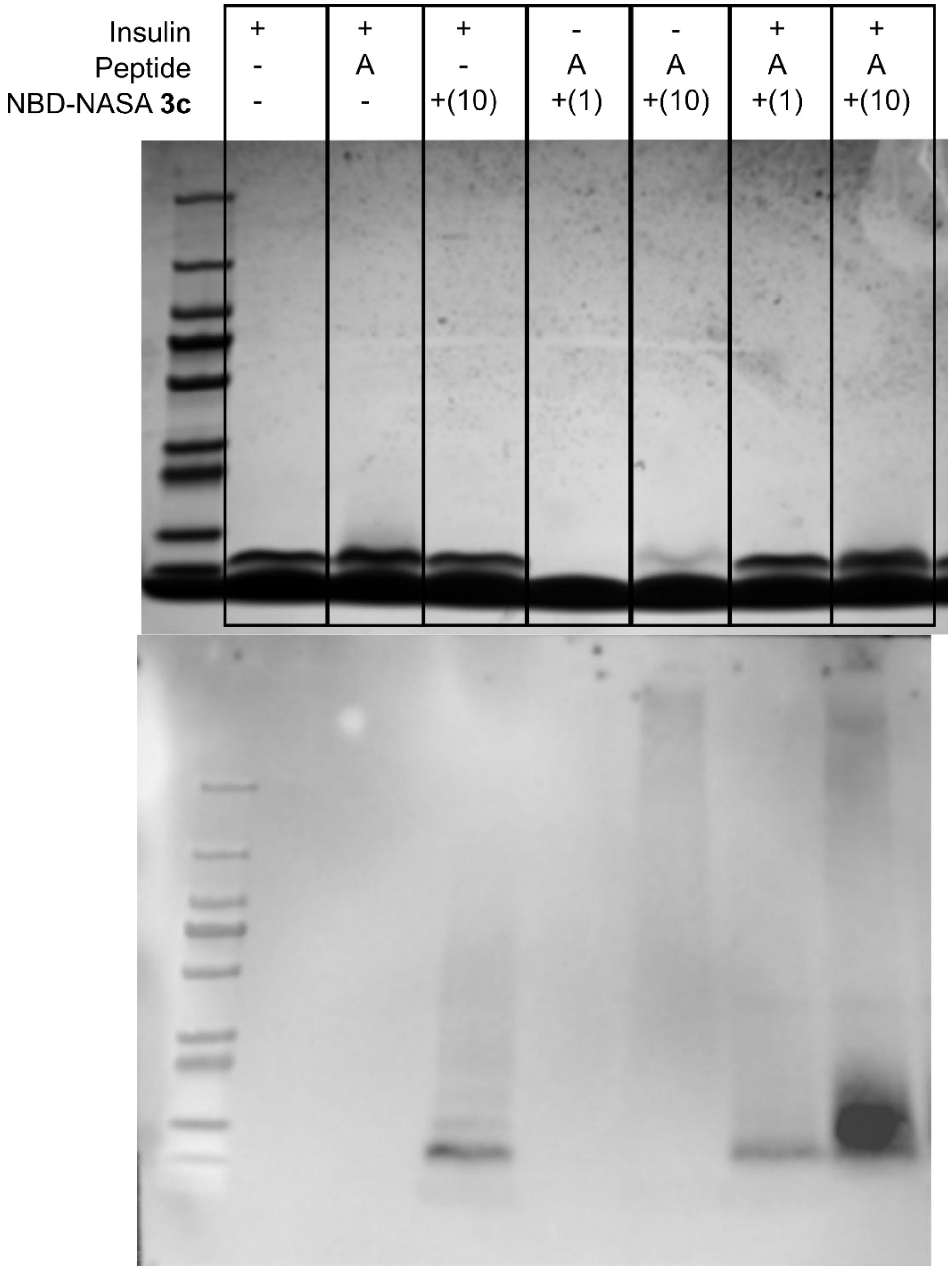

Figure S23: Full version of Fig. S15, showing western blot analysis of insulin labelled with Biotin-NASA **3c** (parentheses denote equivalents w.r.t. peptide) and peptide **(PyOx)$_1$-P1** (A).

9. References

1 Q. P. Peterson, D. C. Hsu, D. R. Goode, C. J. Novotny, R. K. Totten, P. J. Hergenrother, *J. Med. Chem.*, 2009, **52**, 5721-5731.

2 P. R. Werkhoven, M. Elwakiel, T. J. Meuleman, H. C. Q. van Ufford, J. a. W. Kruijtzer and R. M. J. Liskamp, *Org. Biomol. Chem.*, 2015, **14**, 701–710.

3 T. Nanjo, E. C. Jr. de Lucca and M. C. White, *J. Am. Chem. Soc.*, 2017, **139**, 14586–14591.
4 T. Tamura, Z. Song, K. Amaike, S. Lee, S. Yin, S. Kiyonaka and I. Hamachi, *J. Am. Chem. Soc.*, 2017, **139**, 14181–14191.
5 L. Wenskowsky, H. Schreuder, V. Derdau, H. Matter, J. Volkmar, M. Nazaré, T. Opatz and S. Petry, *Angew. Chem. - Int. Ed.*, 2018, **57**, 1044–1048.
6 Z. Shi, P. Peng, D. Strohecker and Y. Liao, *J. Am. Chem. Soc.*, 2011, **133**, 14699–14703.
7 E. Herbst and D. Shabat, *Org. Biomol. Chem.*, 2016, **14**, 3715–3728.
8 L. Ruizendaal, S. P. Pujari, V. Gevaerts, J. M. J. Paulusse and H. Zuilhof, *Chem. – Asian J.*, 2011, **6**, 2776–2786.

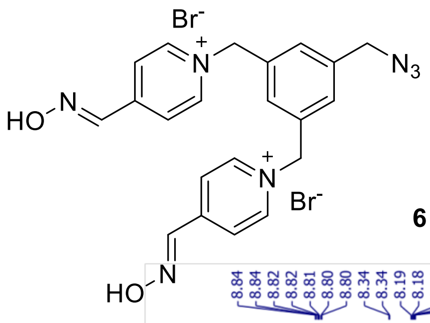

6

^{1}H NMR (400 MHz, D_2O)

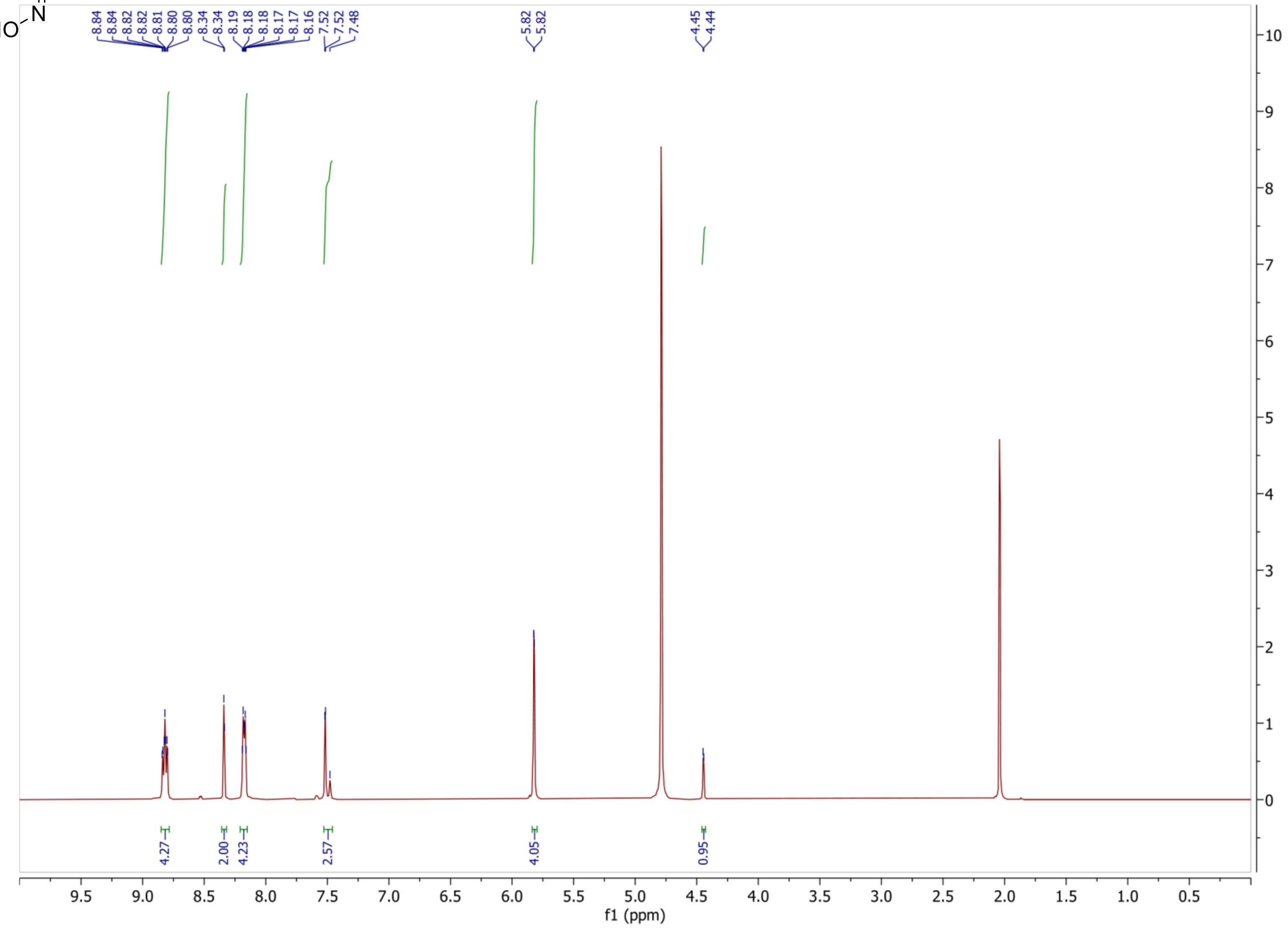

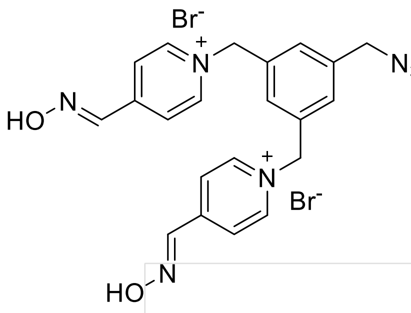

6

^{13}C NMR (101 MHz, D_2O)

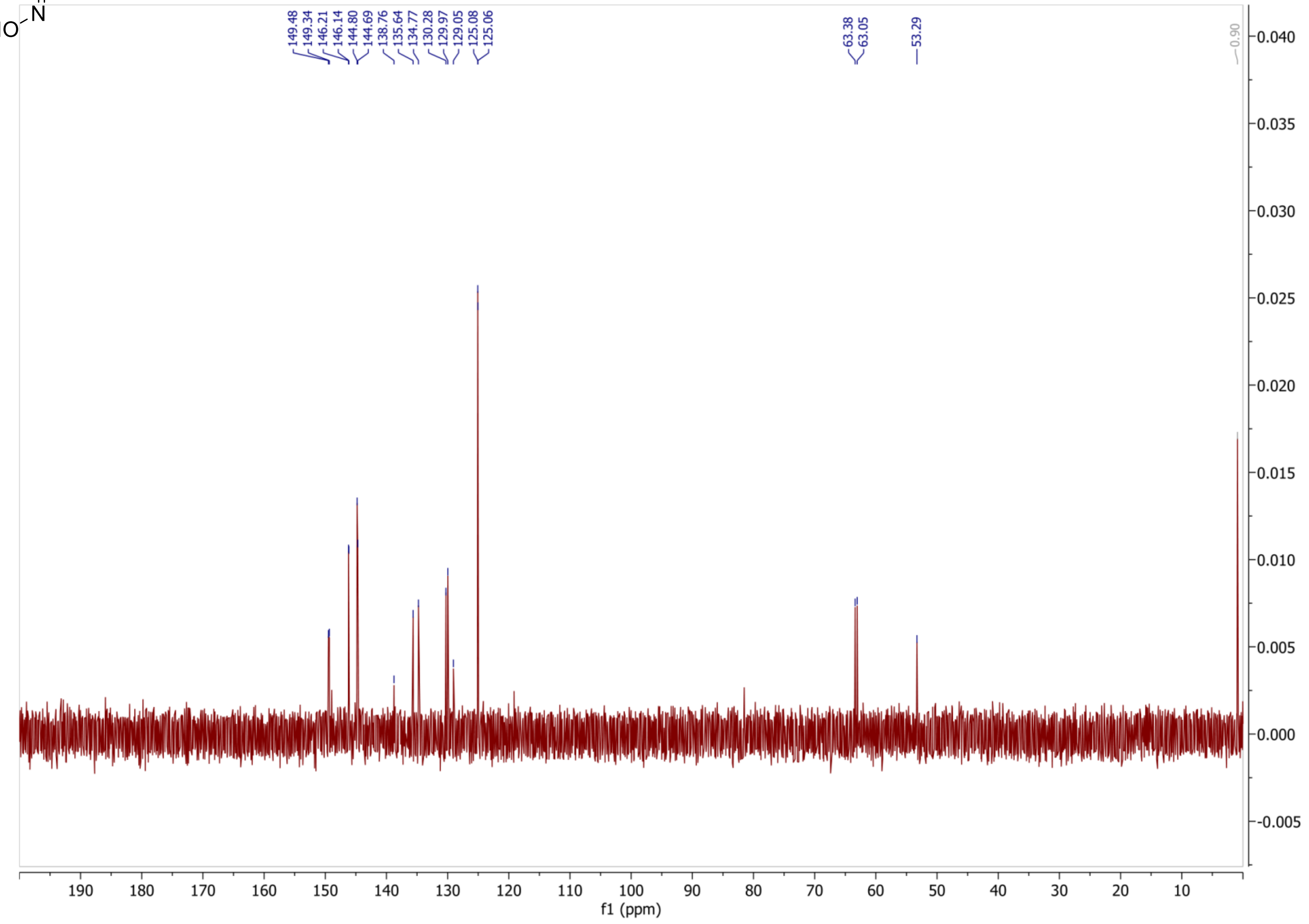

^{1}H NMR (400 MHz, $CDCl_3$)

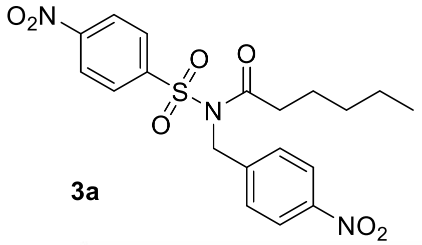

3a

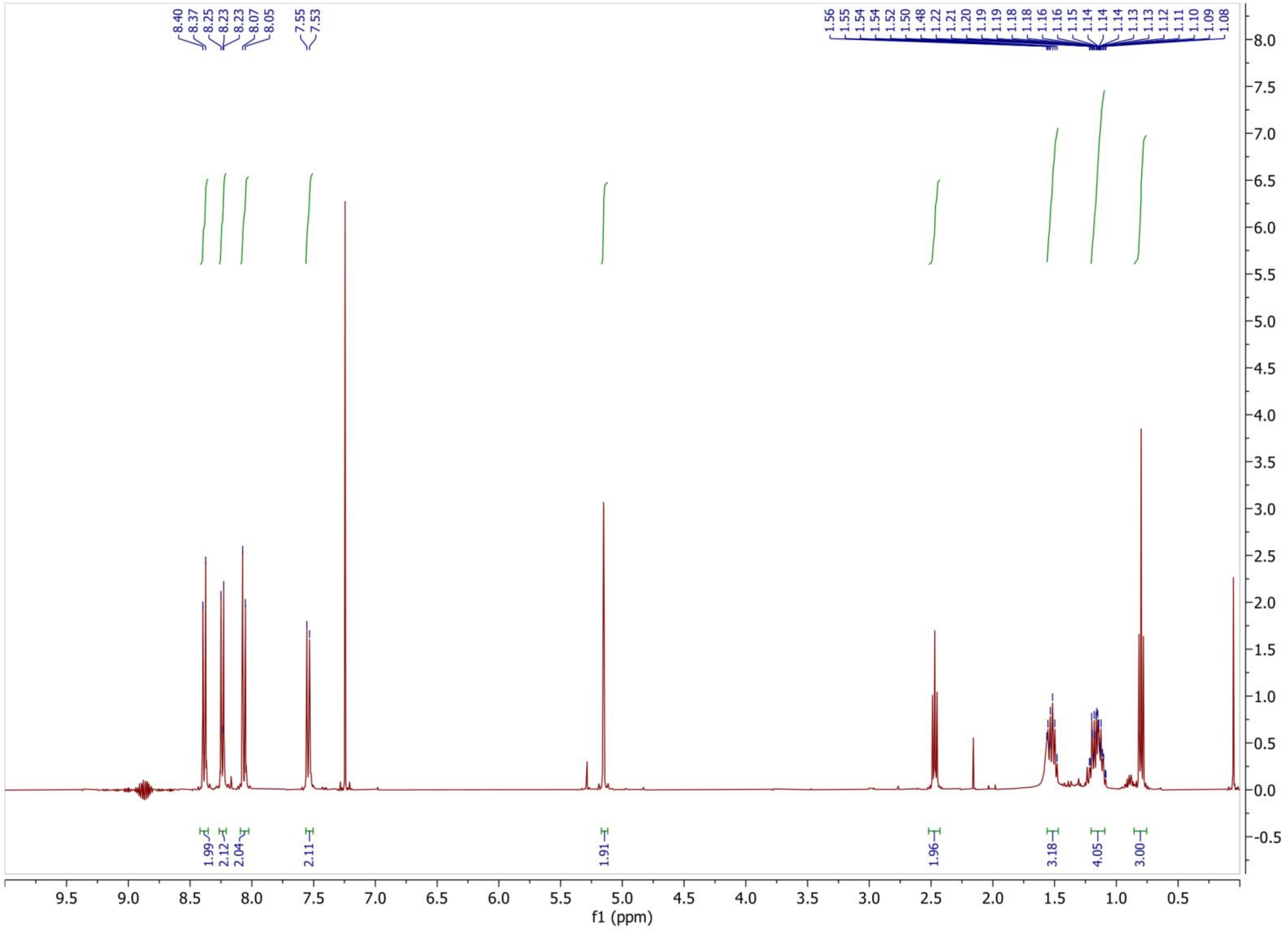

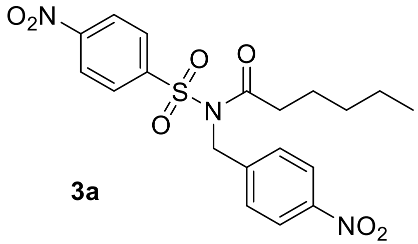

3a

^{13}C NMR (101 MHz, $CDCl_3$)

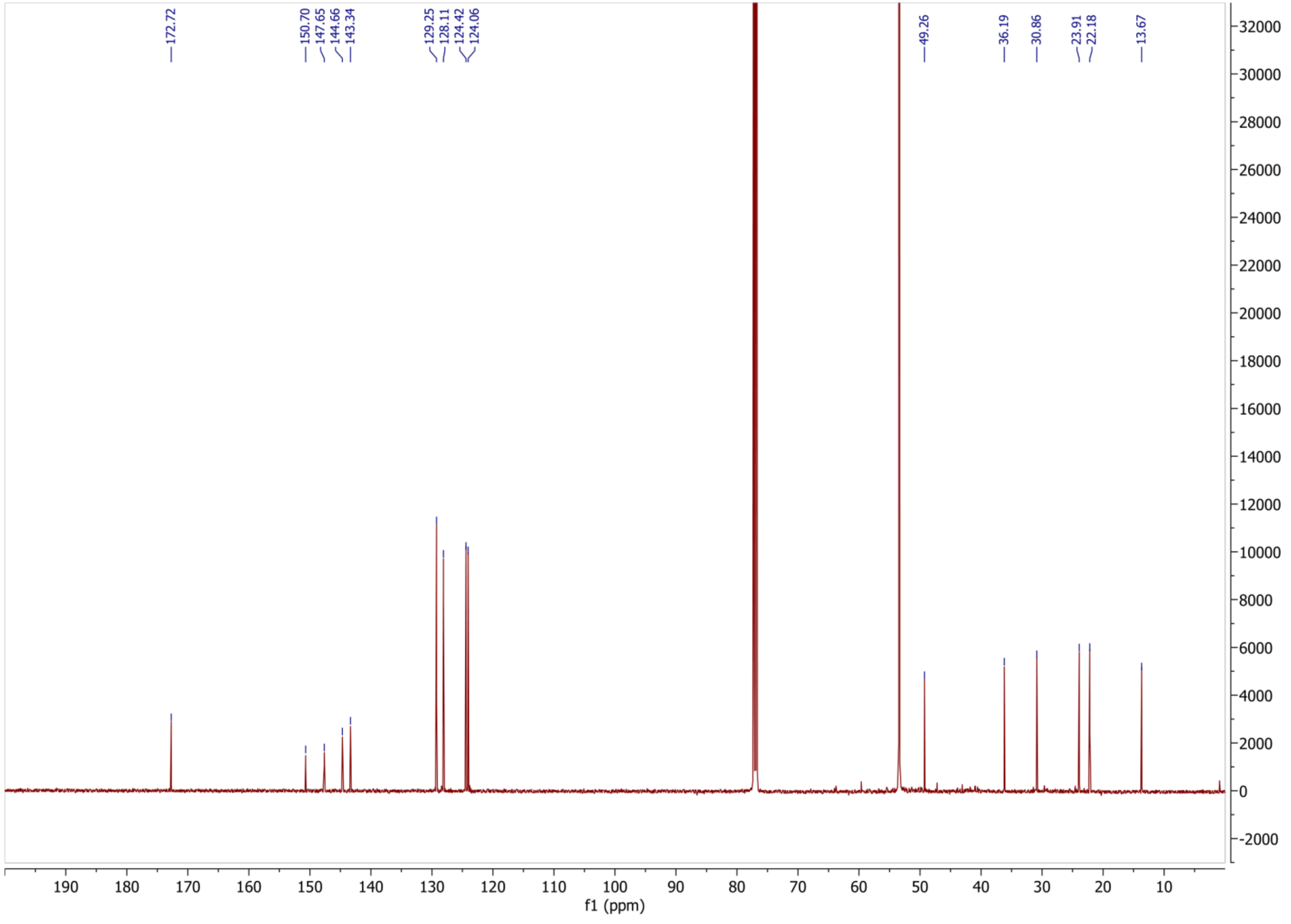

^{1}H NMR (400 MHz, D_2O)

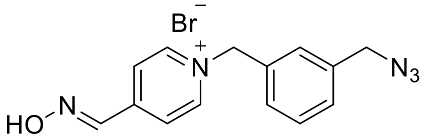

2

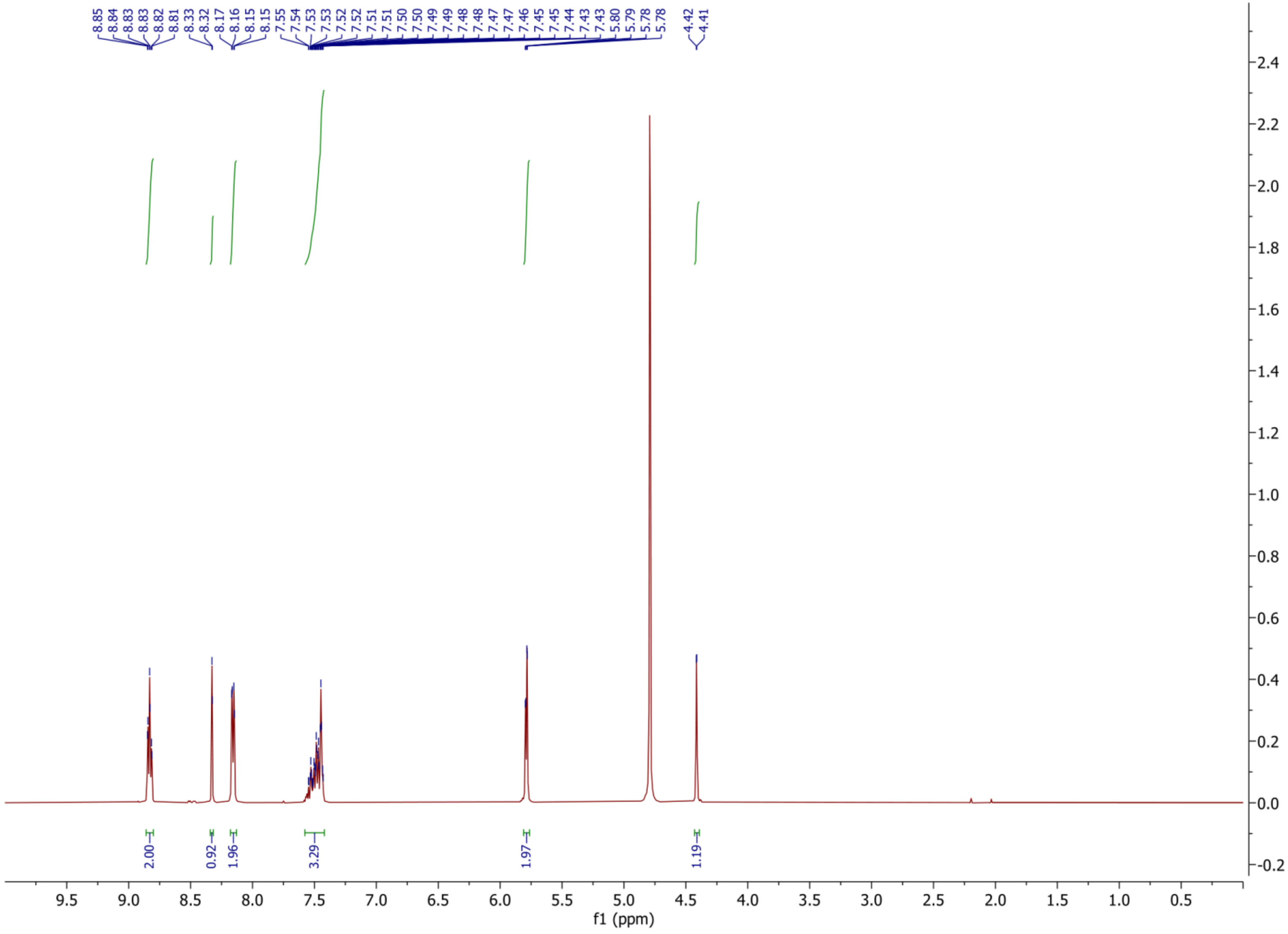

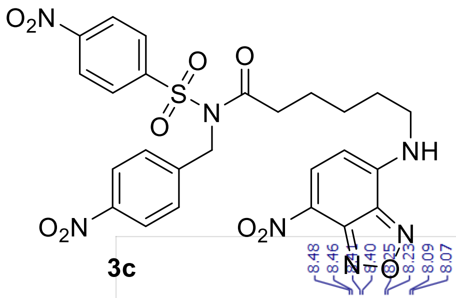

3c

^1H NMR (400 MHz, $CDCl_3$)

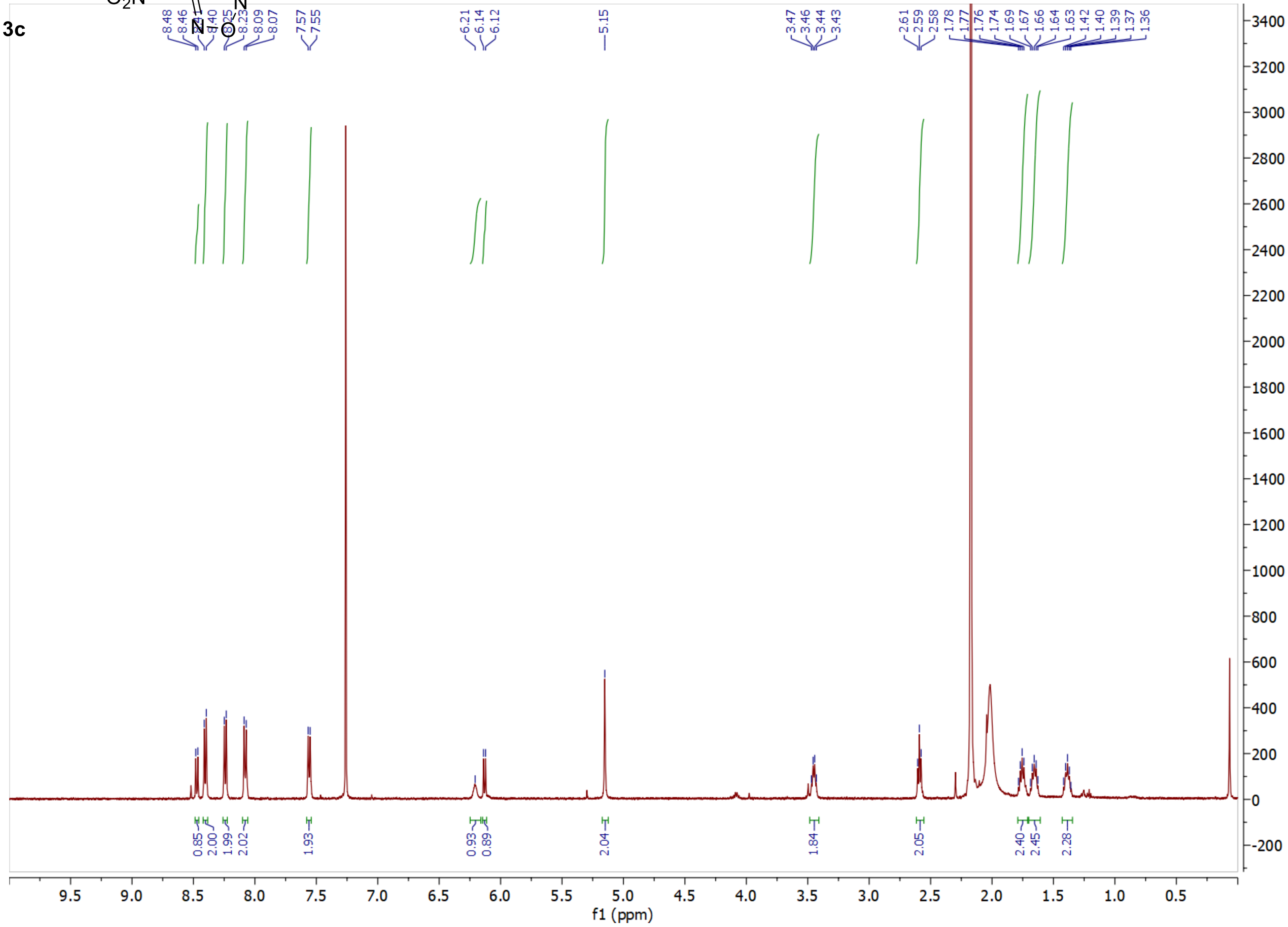

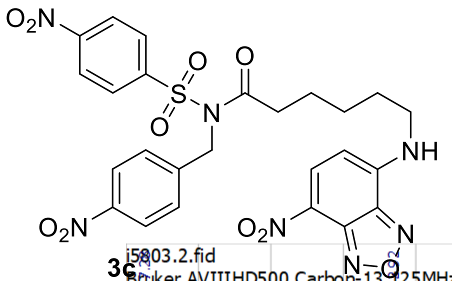

3c

^{13}C NMR (101 MHz, $CDCl_3$)

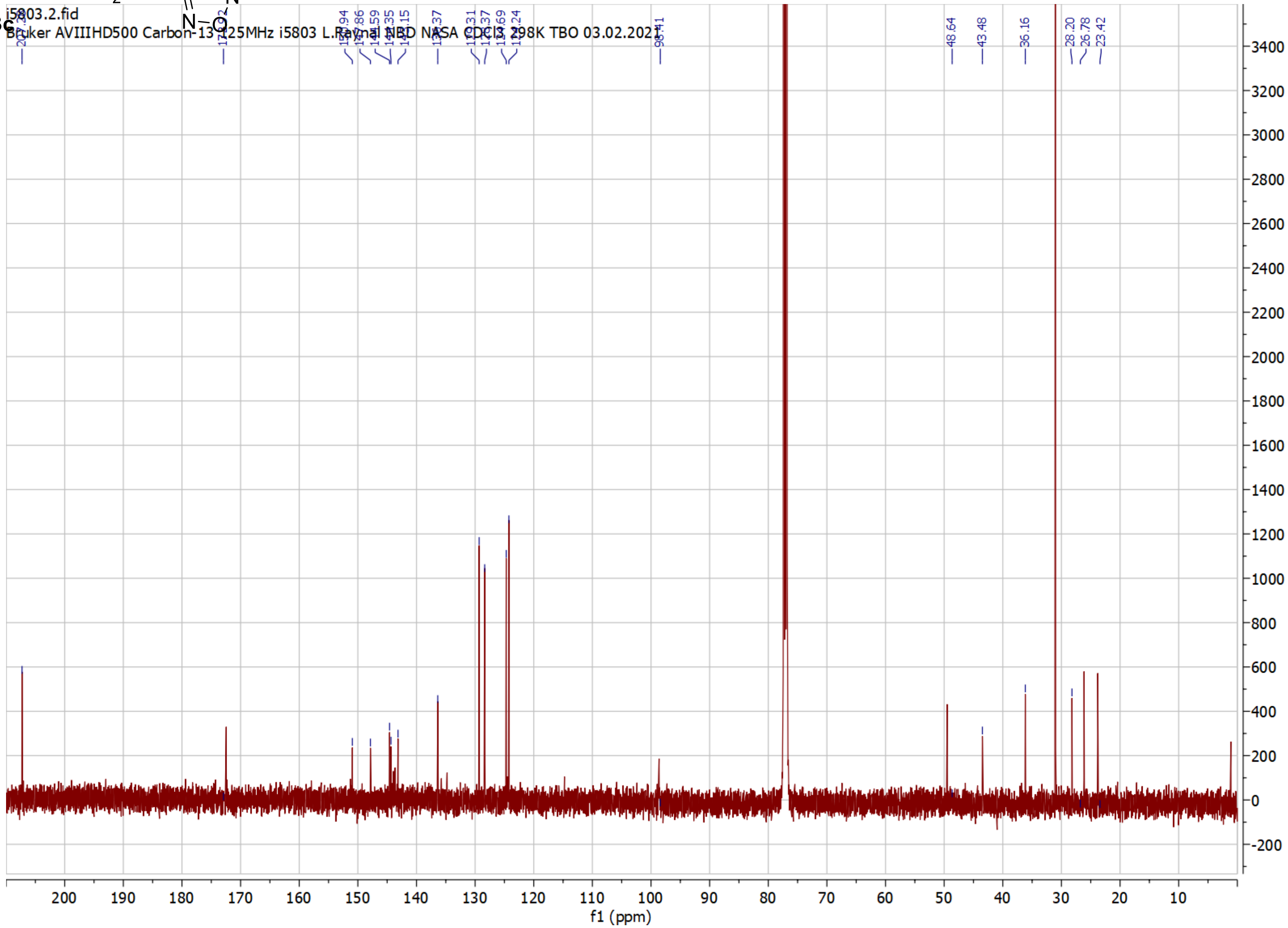

Supplement: Supplementary file 1 [file ao5c07883_si_001.pdf]
